# Genome sequence of the barred knifejaw *Oplegnathus fasciatus* (Temminck & Schlegel, 1844): the first chromosome-level draft genome in the family Oplegnathidae

**DOI:** 10.1093/gigascience/giz013

**Published:** 2019-02-01

**Authors:** Yongshuang Xiao, Zhizhong Xiao, Daoyuan Ma, Jing Liu, Jun Li

**Affiliations:** 1 Institute of Oceanology, Chinese Academy of Sciences, 7 Nanhai Road, Qingdao, 266071, China; 2 Laboratory for Marine Biology and Biotechnology, Qingdao National Laboratory for Marine Science and Technology, 7 Nanhai Road, Qingdao, 266071, China; 3 Center for Ocean Mega-Science, Chinese Academy of Sciences, 7 Nanhai Road, Qingdao, 266071, China

**Keywords:** *Oplegnathus fasciatus*, chromosome-level genome assembly, Hi-C assembly, sex-determining mechanism

## Abstract

**Background:**

The barred knifejaw (*Oplegnathus fasciatus*), a member of the Oplegnathidae family of the Centrarchiformes, is a commercially important rocky reef fish native to East Asia. *Oplegnathus fasciatus* has become an important fishery resource for offshore cage aquaculture and fish stocking of marine ranching in China, Japan, and Korea. Recently, sexual dimorphism in growth with neo-sex chromosome and widespread biotic diseases in *O. fasciatus* have been increasing concern in the industry. However, adequate genome resources for gaining insight into sex-determining mechanisms and establishing genetically resistant breeding systems for *O. fasciatus* are lacking. Here, we analyzed the entire genome of a female *O. fasciatus* fish using long-read sequencing and Hi-C data to generate chromosome-length scaffolds and a highly contiguous genome assembly.

**Findings:**

We assembled the *O. fasciatus* genome with a total of 245.0 Gb of raw reads that were generated using both Pacific Bioscience (PacBio) Sequel and Illumina HiSeq 2000 platforms. The final draft genome assembly was approximately 778.7 Mb, which reached a high level of continuity with a contig N50 of 2.1 Mb. The genome size was consistent with the estimated genome size (777.5 Mb) based on *k*-mer analysis. We combined Hi-C data with a draft genome assembly to generate chromosome-length scaffolds. Twenty-four scaffolds corresponding to the 24 chromosomes were assembled to a final size of 768.8 Mb with a contig N50 of 2.1 Mb and a scaffold N50 of 33.5 Mb using 1,372 contigs. The identified repeat sequences accounted for 33.9% of the entire genome, and 24 003 protein-coding genes with an average of 10.1 exons per gene were annotated using *de novo* methods, with RNA sequencing data and homologies to other teleosts. According to phylogenetic analysis using protein-coding genes, *O. fasciatus* is closely related to *Larimichthys crocea*, with *O. fasciatus* diverging from their common ancestor approximately 70.5–88.5 million years ago.

**Conclusions:**

We generated a high-quality draft genome for *O. fasciatus* using long-read PacBio sequencing technology, which represents the first chromosome-level reference genome for Oplegnathidae species. Assembly of this genome assists research into fish sex-determining mechanisms and can serve as a resource for accelerating genome-assisted improvements in resistant breeding systems.

## Data Description

### Introduction of *O. fasciatus*

**Figure 1:**
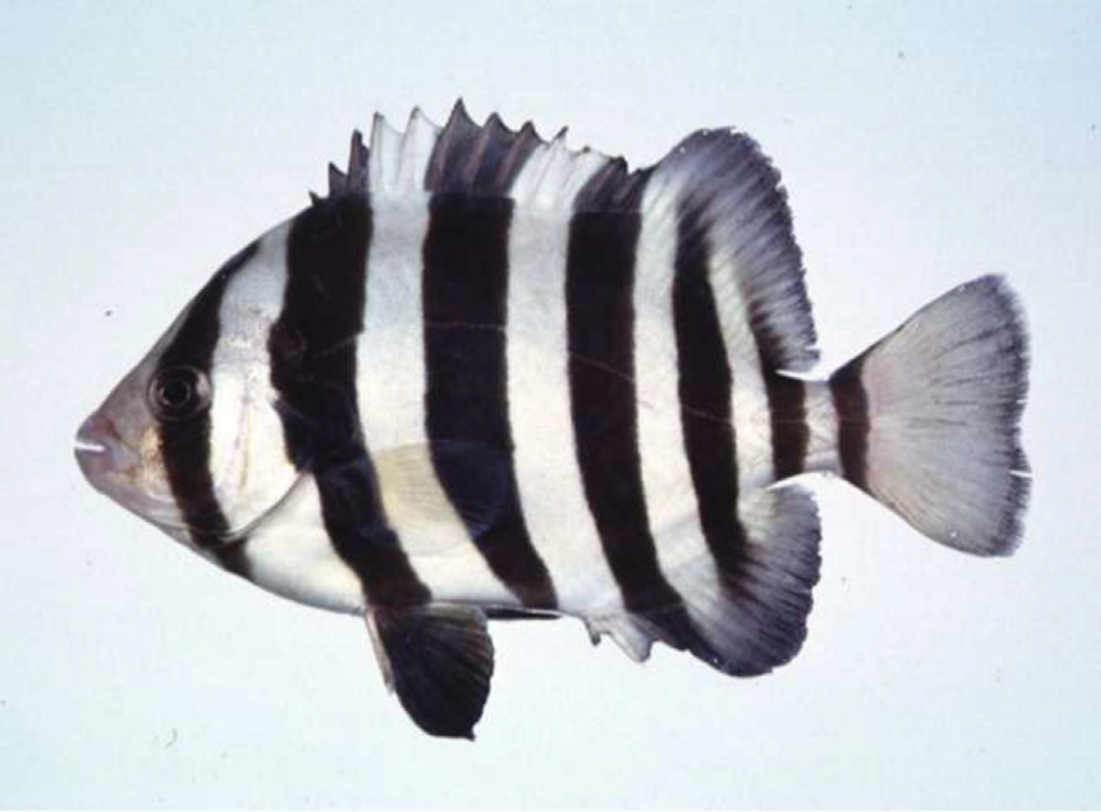
A representative individual of *O. fasciatus*.

The Oplegnathidae family belongs to the order Centrarchiformes, including only one genus *Oplegnathus*, which is comprised of seven species (*Oplegnathus conwayi*, *Oplegnathus fasciatus*, *Oplegnathus insignis*, *Oplegnathus peaolopesi*, *Oplegnathus punctatus*, *Oplegnathus robinsoni*, *and Oplegnathus woodwardi*), two of which (*O. fasciatus* and *O. punctatus*) are commercially valuable in East Asia. The barred knifejaw *O. fasciatus* (NCBI:txid163134, Fishbase ID: 1709) (Temminck and Schlegel, 1844) is one of the two species in the *Oplegnathus* that is commonly found at the depth of 1 to 10 meters in association with rocky reefs [1, 2] and is distributed across a wide range of shallow waters around Korea, Japan, China, and Hawaii [1, 3, 4] (Fig. 1). *Oplegnathus fasciatus* has become an important fishery resource for offshore cage aquaculture and fish stocking of marine ranching in China, Japan, and Korea [5]. It has been reported that the male of *Oplegnathus* possesses a neo-sex chromosome, possibly a sex chromosome Y. The sex chromosome system for *Oplegnathus* is considered to be X_1_ X_1_ X_2_ X_2/_X_1_ X_2_ Y based on karyotype analyses [6, 7]. Furthermore, sexual dimorphism in growth has been detected in the *O. fasciatus*, with male fish exhibiting faster growth than females, possibly due to the sex chromosome system in *Oplegnathus* [8]. *Oplegnathusfasciatus* is vulnerable to viruses (e.g., iridovirus), and genetic degradation caused by inbreeding has led to higher susceptibility to diseases [9, 10]. It is vital to develop genomic resources to gain insight into sex-determining mechanisms and to accelerate the genome-assisted improvements in resistant breeding systems.

To date, a genome sequence with chromosomal assembly of *O. fasciatus* has not been reported. Here, we constructed a high-quality chromosome-level reference genome assembly for *O. fasciatus* using long reads from the Pacific Biosciences (PacBio) DNA sequencing platform and a genome assembly strategy taking advantage of the genome assembly program Canu [11]. This genome assembly of *O. fasciatus* is the first chromosome-level reference genome constructed for the Oplegnathidae family. The completeness and continuity of the genome will provide high-quality genomic resources for studies on sex-determining mechanisms and for accelerating the genome-assisted improvements in resistant breeding systems.

### Genomic DNA extraction and genome size estimation

**Figure 2:**
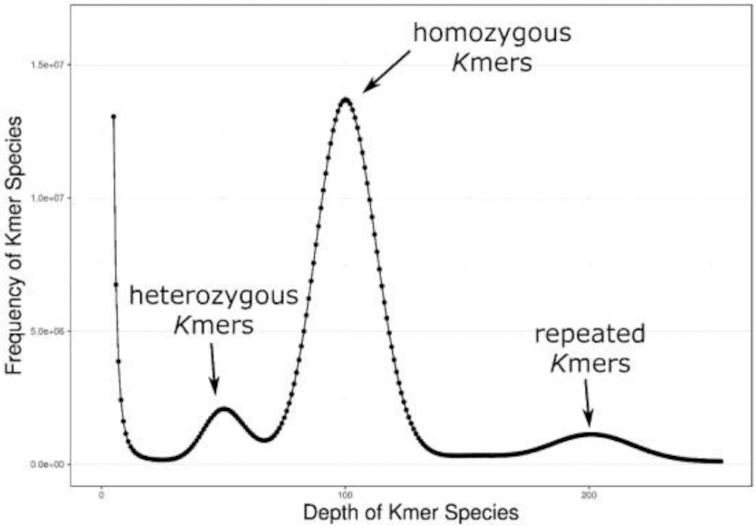
*k*-mer distribution of the *O. fasciatus* genome.

High-quality genomic DNA for sequencing using the Illumina platform (Illumina Inc., San Diego, CA) and PacBio Sequel sequencing (Pacific Biosciences of California, Menlo Park, CA) was extracted from fresh muscle tissue and blood samples from a single female *O. fasciatus*. The fish was collected from the near-shore area of Qingdao City (Yellow Sea), Shandong Province, China. The whole-genome size of *O. fasciatus* was estimated based on Illumina DNA sequencing technology. A short-insert library (300∼350 bp) was constructed and generated a total of ∼90.7 Gb of raw reads using the standard protocol provided by the Illumina HiSeq X Ten platform (Illumina Inc., San Diego, CA). After the removal of low-quality and redundant reads, we obtained ∼80.8 Gb of clean data for *de novo* assembly to estimate the whole-genome size (Supplementary Table S1, Fig. 2). All cleaned reads were subjected to 17-mer frequency distribution analysis [12]. As the total number of *k*-mers was approximately 8.09 × 10^10^ and the peak of *k*-mers was at a depth of 100, the genome size of *O. fasciatus* was calculated to be 777.5 Mb using the following formula with amendment: G = (N*_k_*_-mer_—N_error__*_k_*_-mer_)/D, where G is genome size, N_k-mer_ is the number of *k*-mers, N_error__*_k_*_-mer_ is the number of *k*-mers with the depth of 1, and D is the *k*-mer depth (Fig. 2). Meanwhile, an estimated heterozygosity of 0.29% and a repeat content of 38.46% were detected for *O. fasciatus* in this work. A pilot genome assembly was approximately 744.5 Mb with a contig N50 of 7.2 kb and a scaffold N50 of 84.1 kb using the Illumina data and the assembly program Platanus [13] (Supplementary Table S2). The GC content was 41% (Supplementary Fig. S1). This first attempt at a genome assembly was of low quality, partly due to its high genomic repeat content.

### Genome assembly using PacBio long reads

Two 20 kb genomic DNA libraries were constructed and sequenced using the PacBio Sequel platform, generating 62.9 Gb raw DNA reads. We obtained 4.8 million subreads (62.8 Gb in total) with an N50 read length of ∼22 kb after removing adaptor (Supplementary Table S1).

Canu v1.4 (Canu, RRID:SCR_015880) was first used to assemble the genome with the Corrected-Error-Rate parameter set at 0.040 [11]. As a result, a genome assembly with a total length of 875.9 Mb was constructed for *O. fasciatus*, slightly higher than the genome size estimated by 17-mer analysis based on the Illumina data (Supplementary Table S2). The genome complexity, such as structural variants and heterozygosity, might be possible reasons to explain the relatively large genome size in the assembly. We therefore applied Redundans v0.13c [14] to remove the sequence redundancy and obtain a genome assembly size of 778.0 Mb. We then used the Arrow tool in SMRT Link 5.0 software with the minCoverage parameter set at 15 to implement error correction based on the PacBio long reads data (Table 1). The resulting genome assembly was further polished using Illumina Next-generation sequencing (NGS) data, which were used in the genome survey analysis above. The final draft genome assembly was 778.7 Mb, which reached a high level of continuity with a contig N50 length of 2.1 Mb (Table 1). The contig N50 of *O. fasciatus* was much higher than those of previous fish genome assemblies constructed using NGS DNA sequencing technologies and is comparable to those of recently reported model fish species (Supplementary Table S3). Previous studies illuminated the relationship between read length and genome assembly; therefore, we attributed the continuity of the genome primarily to the application of long reads in the assembly.

**Table 1:** Summary of *Oplegnathus fasciatus* genome assembly and annotation

Genome assembly		
	Draft scaffolds	Chromosome-length scaffolds based on Hi-C
Length of genome (bp)	778,731,089	768,808,243
Number of contigs	1,692	1,372
Contigs N50 (bp)	2,149,025	2,130,780
Number of scaffolds	/	24
Scaffold N50 (bp)	/	33,548,962
Genome coverage (X)	314.6	
Number of contigs (≥100 kb)	693	708
Total length of contigs (≥100 kb)	735,235,962	732,827,446
Mapping rate of contigs (≥100kb)(%)	/	99.67
Genome annotation		
Protein-coding gene number	24,003	
Mean transcript length (kb)	16.1	
Mean exons per gene	10.1	
Mean exon length (bp)	217.7	
Mean intron length (bp)	1,527.4	

### Hi-C library construction and chromosome assembly

Hi-C is a sequencing-based approach for determining chromosome interactions by calculating the contact frequency between pairs of loci, which are strongly dependent upon the one-dimensional distance, in base pairs, between a pair of loci [15, 16]. In this work, we used Hi-C to construct the genome assembly of *O. fasciatus*.

**Figure 3:**
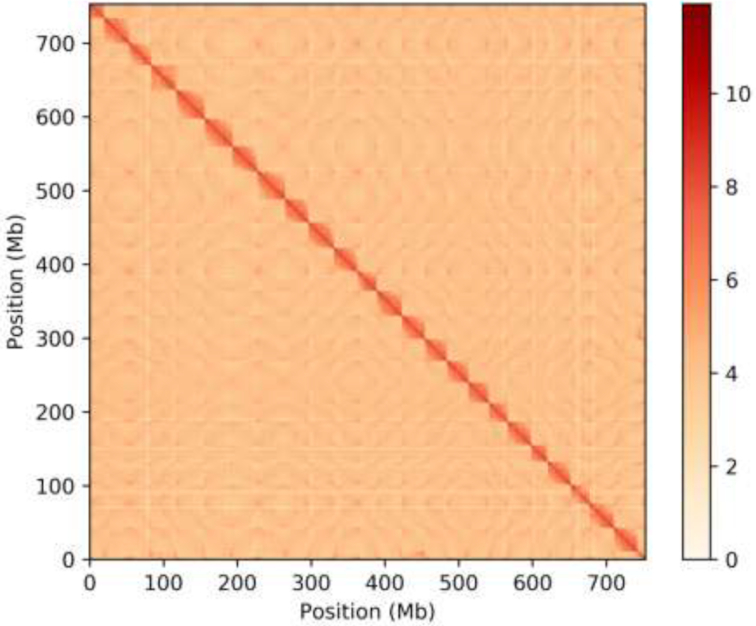
Hi-C interaction heat map for *O. fasciatus* reference genome showing interactions between the 24 chromosomes.

Genomic DNA was extracted for the Hi-C library from a whole-blood sample of *O. fasciatus* as previously described [17]. Cells were fixed with formaldehyde and lysed, and the cross-linked DNA was digested with MboI. Sticky ends were biotin-labeled and proximity ligated to form chimeric junctions and then physically sheared to a size of 300–500 bp [17]. Chimeric fragments representing the original cross-linked, long-distance physical interactions were then processed into paired-end sequencing libraries, and 629 million 150-bp paired-end Illumina reads (91.5 Gb) were produced with Q20 and Q30 of ∼94.0% (Supplementary Tables S1, S4). By mapping the Hi-C data to the PacBio-based assembly using BWA software (BWA, RRID:SCR_010910), we found that sequencing data with mates mapped to a different contig (or scaffold) and data mapped to a different contig (or scaffold) (map Q5≥ 5) were 593.7 Mb (94.4%), 240.5 Mb (40.5%), and 205.1 Mb (34.6%), respectively (Supplementary Table S4). We then employed BWA and Lachesis software to align paired-end reads to filter all base sequences more than 500 bp from each restriction site [18]. According to the conduct of clustering, ordering, and orienting to the assembly contigs (1,692), these sequences were grouped into 24 chromosome clusters and scaffolded using Lachesis software with tuned parameters [19] (Supplementary Table S4, Fig. 3). Finally, we constructed the chromosome interactions map using Juicer software and employed the JucieBox to complete the visual correction of the interaction map. We obtained 1,756 polished contigs by interrupting misassembly from 1,692 contigs. Twenty-four scaffolds were assembled corresponding to the 24 chromosomes of *O. fasciatus* based on the karyotype analyses [6, 7] (Supplementary Table S4, Fig. 3).

A final size of 768.8 Mb accounting for the 98.7% draft genome was assembled, which showed a high level of continuity with a contig N50 of 2.1 Mb and a scaffold N50 of 33.5 Mb using 1,372 contigs. The anchor rate of contigs (>100 kb) to chromosomes was attained up to the 99.7% based on the Hi-C assembly (Table 1). The contig N50 and scaffold N50 of *O. fasciatus* were much higher than those of previous fish genome assemblies constructed using NGS DNA sequencing technologies based on the genome assembly using PacBio long reads and Hi-C assembly (Supplementary Table S3).

### Genome quality evaluation

To assess the completeness of the assembled *O. fasciatus* genome, we subjected the assembled sequences to Benchmarking Universal Single-Copy Orthologs (BUSCO) version 3 evaluation (BUSCO. RRID:SCR_015008) (BUSCO, actinopterygii_odb9) [20]. Overall, 96.6% and 1.5% of the 4584 expected actinopterygii genes were identified in the assembled genome as complete and partial BUSCO profiles, respectively. Approximately 85 genes could be considered missing in our assembly (Supplementary Table S5). Among the expected complete actinopterygii genes, 4,259 and 171 were identified as single copy and duplicated BUSCOs, respectively (Supplementary Table S5). We then used Minimap2 to estimate the completeness and homogeneity of genome assembly based on continuous long read subreads. A high quality of completeness and homogeneity was assessed in the genome assembly, and the mapping rate, coverage rate, and average sequencing depth reached 90.2%, 99.9%, and 80.6, respectively (Supplementary Table S6). Note that the mapping ratio might be related to the repetitive content of the *O. fasciatus* genome, especially for the high repeat content in the sex chromosomes [6]. However, how the repetitive elements in the genome influence the karyotypes of this species needs further investigation.

To further evaluate the accuracy of the *O. fasciatus* genome assembly, we aligned the NGS-based short reads from the whole-genome sequencing data against the reference genome using BWA [21]. We then used GATK (GATK, RRID:SCR_001876) to implement single-nucleotide polymorphism (SNP) calling and filter work, and the results showed that 99.8% and 0.2% of the 1.6 × 10^6^ expected SNP reads were identified in the assembled genome as heterozygous and homologous SNPs, respectively. SNP calling on the final assembly also yielded a heterozygosity rate of 0.20%, supporting the *k*-mer estimate analysis (0.29%) (Supplementary Table S7).

### Repeat sequences within the *O. fasciatus* genome assembly

To identify tandem repeats, we utilized Tandem Repeat Finder to annotate repetitive elements in the *O. fasciatus* genome. RepeatModeler (RepeatModeler, RRID:SCR_015027) (version 1.04) and LTR_FINDER (LTR_Finder, RRID:SCR_015247) [22] were used to construct a *de novo* repeat library with default parameters. Subsequently, we used RepeatMasker (RepeatMasker, RRID:SCR_012954) [23] (version 3.2.9) to map our assembled sequences on the Repbase TE (version 14.04) [24] and the *de novo* repeat library to identify known and novel transposable elements (TEs). In addition, TE-related proteins were annotated by using RepeatProteinMask software (version 3.2.2) [23].

The identified repeat sequences accounted for 33.9% of the *O. fasciatus* genome, including repeat sequences with 23.6% of the genome based on the *de novo* repeat library (Table 2). Approximately 23.4% of the *O. fasciatus* genome was identified as interspersed repeats (most often TEs). Among them, DNA TEs were the most abundant type of repeat sequences, which occupied 11.5% of the whole genome. Long interspersed nuclear elements and long terminal repeats comprised 7.3% and 4.0% of the whole genome, respectively (Table 2, Supplementary Fig. S2).

**Table 2:** Detailed classification of repeat sequences of *Oplegnathus fasciatus*

	Repbase TEs		TE proteins		De novo		Combined TEs	
Type	Length (bp)	% in genome	Length (bp)	% in genome	Length (bp)	% in genome	Length (bp)	% in genome
DNA	39,147,527	5.03	5,390,266	0.69	93,089,344	11.95	124,417,402	15.98
Long interspersed nuclear element	23,983,322	3.08	16,460,762	2.11	57,167,551	7.34	85,761,250	11.01
Short interspersed nuclear element	875,585	0.11	0	0.00	914,559	0.12	1,747,250	0.22
Long terminal repeat	10,163,601	1.31	5,770,483	0.74	31,126,639	4.00	42,465,968	5.45
Satellite	2,028,992	0.26	0	0.00	2,613,480	0.34	4,361,048	0.56
Simple_repeat	1,556,026	0.20	0	0.00	5,179,965	0.67	6,386,303	0.82
Other	6,545	0.00	0	0.00	0	0.00	6,545	0.00
Unknown	331,430	0.04	0	0.00	20,636,768	2.65	20,967,052	2.69
Total	73,544,786	9.44	27,613,880	3.55	183,954,095	23.62	250,611,845	32.18

### RNA preparation and sequencing

We sequenced cDNA libraries prepared from the eggs of *O. fasciatus* that were used for genome annotation using Illumina sequencing technology. RNA quality was determined based on the estimation of the ratio of absorbance at 260 nm/280 nm (OD = 2.0) and the RIN (value = 9.2) by using a Nanodrop ND-1000 spectrophotometer (LabTech, USA) and a 2100 Bioanalyzer (Agilent Technologies, USA), respectively. We used the Clontech SMARTer cDNA synthesis kit to complete reverse transcription. A paired-end library was prepared following the Paired-End Sample Preparation Kit manual (Illumina Inc., San Diego, CA). Finally, a library with an insert length of 300 bp was sequenced by Illumina HiSeq X Ten in 150PE mode (Illumina Inc., San Diego, CA). As a result, we obtained ∼42.2 Gb high-quality transcriptome data from RNA-seq (Supplementary Tables S1, S8).

### Gene annotation

Gene annotation of the *O. fasciatus* genome was performed using *de novo*, homology-based, and transcriptome sequencing-based predictions. We employed Augustus (Augustus, RRID:SCR_008417) (version 2.5.5) [25] and GenScan (GENSCAN, RRID:SCR_012902) (version 1.0) [26] software to predict protein-coding genes in the *O. fasciatus* genome assembly. Protein sequences of closely related fish species including *Larimichthys crocea, Lates calcarifer, Gasterosteus aculeatus, Paralichthys olivaceus, Cynoglossus semilaevis*, and *Gadus morhua* were downloaded from Ensembl [27] and aligned against the *O. fasciatus* genome using TBLASTN (TBLASTN, RRID:SCR_011822) software [28]. Subsequently, Genewise2.2.0 (GeneWise, RRID:SCR_015054) software [29] was employed to predict potential gene structures on all alignments.

We also mapped these NGS transcriptome short reads onto our genome assembly using TopHat1.2 (TopHat, RRID:SCR_013035) software [30], and then we employed Cufflinks (Cufflinks, RRID:SCR_014597) [31] to predict gene structures (Supplementary Table S9). All gene models were then integrated using MAKER (MAKER, RRID:SCR_005309) to obtain a consensus gene set [32]. The final total gene set was composed of 24,003 genes with an average of 10.1 exons per gene in the *O. fasciatus* genome (Table 1). The gene number, gene length distribution, coding sequence length distribution, exon length distribution, and intron length distribution were all comparable with those of other teleost fish species (Supplementary Table S9, Fig. S3).

To obtain further functional annotation of the protein-coding genes in the *O. fasciatus* genome, we employed the local BLASTX (BLASTX, RRID:SCR_001653) and BLASTN (BLASTN, RRID:SCR_001598) programs and the Swiss-prot database with an e-value ≤ 1e-5 [33] to align the non-redundant nucleotide and non-redundant protein, respectively. We also used Blast2GO (Blast2GO, RRID:SCR_005828) software to search the Gene Ontology, and Kyoto Encyclopedia of Genes and Genomes pathway databases [34, 35, 36]. Ultimately, 97.3% (23,364 genes) of the 24,003 genes were annotated by at least one database (Supplementary Table S10). Four types of non-coding RNAs (microRNAs, transfer RNAs, ribosomal RNAs, and small nuclear RNAs) were also annotated using the tRNAscan-SE (tRNAscan-SE, RRID:SCR_010835) and the Rfam database [37, 38] (Supplementary Table S11).

### Gene family identification and phylogenetic tree construction

**Figure 4:**
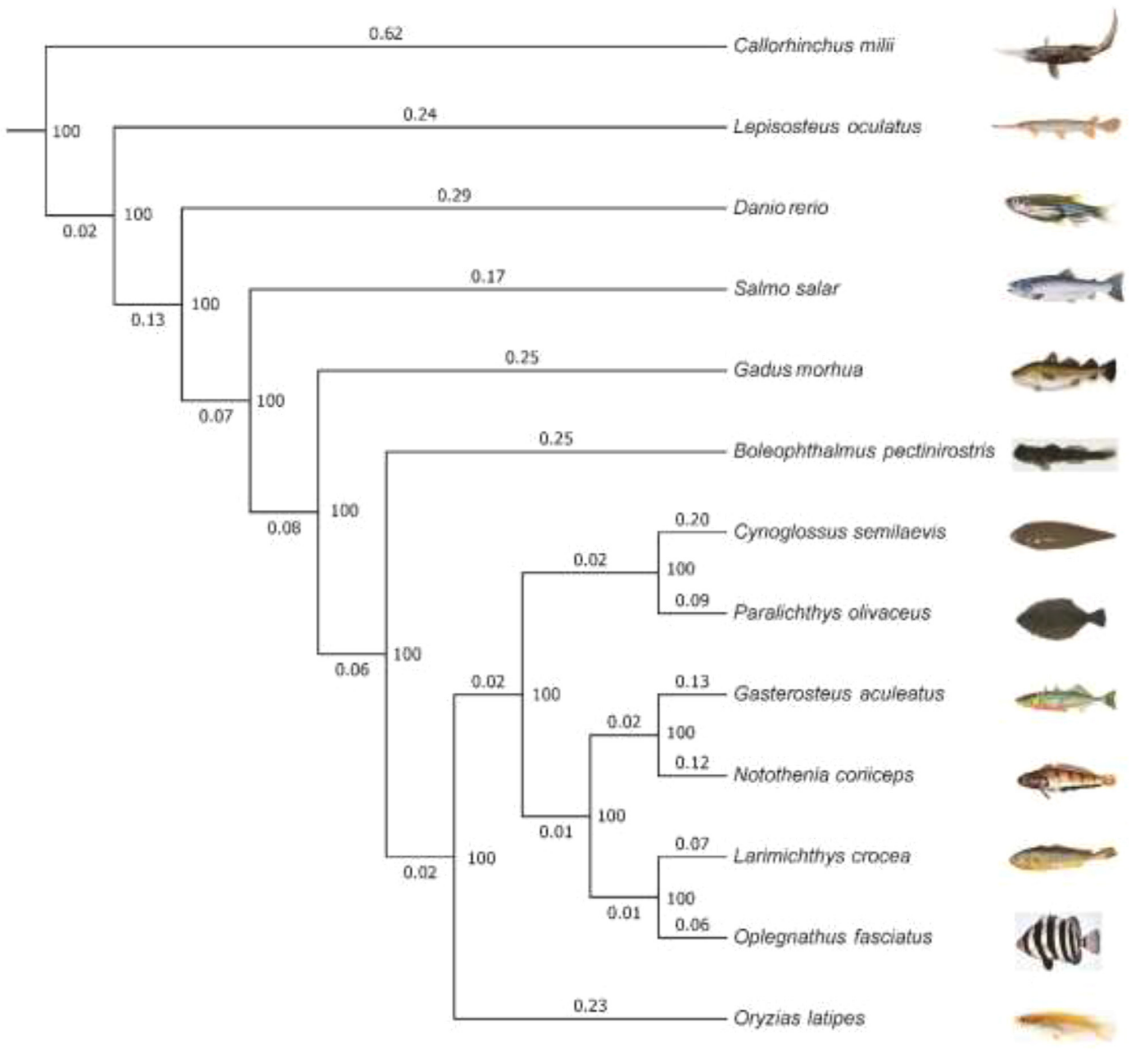
The phylogenetic relationships of *O. fasciatus* with other fishes. The bootstrap values (larger than 1) calculated from 1,000 bootstrap replicates and the branch lengths (smaller than 1) were labeled at and below/above each branch, respectively.

We employed the BLASTP (BLASTP, RRID:SCR_001010) program [39] with an e-value threshold of 1e-5 to identify gene families based on the transcript alignments of each gene from *O. fasciatus* and other fish species, which included *Larimichthys crocea*, *Gadus morhua*, *Paralichthys olivaceus*, *Cynoglossus semilaevis*, *Notothenia coriiceps*, *Boleophthalmus pectinirostris*, *Lepisosteus oculatus*, *Gasterosteus aculeatus*, *Callorhinchus milii*, *Danio rerio*, *Salmo salar*, and *Oryzias latipes*. A total of 21,528 gene families were identified by clustering the homologous gene sequences based on H-scores calculated from Bit-score using Hcluster_sg software (Supplementary Fig. S4). Subsequently, we selected 1,236 single-copy orthogroups from the above-mentioned species to construct the phylogenetic relationship between *O. fasciatus* and other fish species. We used the ClustalW (ClustalW, RRID:SCR_002909) program [40] to extract and align coding sequences of single-copy genes from the 1,158 orthogroups with a length filter (Supplementary Fig. S5). All the alignments were concatenated as a single dataset for each species. Nondegenerated sites extracted from the dataset were then joined into new sequences for each species to construct a phylogenetic tree based on the maximum-likelihood method implemented in the PhyML package [41] (with the -m PROTGAMMAAUTO model). We used the MCMCtree program to estimate divergence times among species based on the approximate likelihood method [42] and molecular clock data from the divergence time between medaka from the TimeTree database [43]. According to the phylogenetic analysis, *O. fasciatus* (Eupercaria: Centrarchiformes) clustered with *Larimichthys crocea* in the order Perciformes (Eupercaria), which was consistent with the new fish species taxonomy [44] (Fig. 4). The divergence time between *O. fasciatus* and the common ancestor with *Larimichthys crocea* was at approximately 70.5–88.5 million years ago.

## Conclusions

We successfully assembled the genome of *O. fasciatus* and reported the first chromosome-level genome sequencing, assembly, and annotation based on long reads from the third-generation PacBio Sequel sequencing platform. The final draft genome assembly is approximately 778.7 Mb, which was slightly higher than the estimated genome size (777.5 Mb) based on *k*-mer analysis. Those contigs were scaffolded to chromosomes using Hi-C data, resulting in a genome with a high level of continuity, with a contig N50 of 2.1 Mb and a scaffold N50 of 33.5 Mb. The chromosome-level genome assembly of *O. fasciatus* is also the first high-quality genome in the Oplegnathidae family. We also predicted 24,003 protein-coding genes from the generated assembly, and 97.3% (23,364 genes) of all protein-coding genes were annotated. We found that the divergence time between *O. fasciatus* and its common ancestor with *Larimichthys crocea* was approximately 70.5–88.5 million years ago. As far as we know, the Y chromosomes has always exhibited many specific sequence characteristics compared to X1 and X2, such as repeat content, and those differences might increase the difficulty of the sequence assembly of chromosomes X1 and X2. The chromosome-level genome assembly together with gene annotation data generated for the female fish in this work will provide a valuable resource for further research on sex-determining mechanisms, especially for obtaining an accurate assembly of the Y chromosome in male fish. These results will also accelerate genome-wide association studies in resistant breeding systems.

## Supplementary Material

GIGA-D-18-00300_Original_Submission.pdf

GIGA-D-18-00300_Revision_1.pdf

GIGA-D-18-00300_Revision_2.pdf

Response_to_Reviewer_Comments_Original_Submission.pdf

Response_to_Reviewer_Comments_Revision_1.pdf

Reviewer_1_Report_Original_Submission -- Christiaan Henkel 10/1/2018 Reviewed

Reviewer_1_Report_Revision_1 -- Christiaan Henkel 1/7/2019 Reviewed

Reviewer_2_Report_Original_Submission -- Alejandro Gutierrez 10/3/2018 Reviewed

Supplemental Files

## Data Availability

Supporting data and materials are available in the *GigaScience* GigaDB database [45], with the raw sequences deposited in the NCBI Sequence Read Archive under the accessions SRP158313 and SRP160016 .

## Additional files

S Fig. 1 The GC content of *O. fasciatus* base on the Illumina platform for genome size survey

S Fig. 2 The content of interspersed repeats of *O. fasciatus* genome assembly. (a) Repbase library (b) *de novo* library

S Fig. 3 The gene number, gene length distribution, coding sequence length distribution, exon length distribution and intron length distribution were all comparable with those in other teleost fish species.

S Fig. 4 Comparing genome assemblies between *O. fasciatus* and other fish species.

S Fig. 5 Orthologous gene families across four fish genomes (*Oplegnathus fasciatus*, *Larimichthys crocea*, *Gadus morhua* and *Salmo salar*)

S Table 1 Summary of sequence data from *O. fasciatus*

S Table 2 Genome assembly statistics for *O. fasciatus*

S Table 3 Comparing genome assemblies between *O. fasciatus* and other fish species. Some data were cited from the reference (Gaorui Gong, Cheng Dan, Shijun Xiao, Wenjie Guo, Peipei Huang, Yang Xiong, Junjie Wu, Yan He, Jicheng Zhang, Xiaohui Li, Nansheng Chen, Jian-Fang Gui, Jie Mei; Chromosomal-level assembly of yellow catfish genome using third-generation DNA sequencing and Hi-C analysis, GigaScience, Volume 7, Issue 11, 1 November 2018, giy120, doi.org/10.1093)

S Table 4 Alignment of clean reads for Hi-C data and Hi-C libraries for chromosome-scale assembly

S Table 5 Genome quality of *Oplegnathus fasciatus* based on the BUSCO assessment

S Table 6 The estimation of the completeness for *O. fasciatus* genome assembly based on CLR (Continuous Long Reads) subreads mapping

S Table 7 The estimation of the accuracy for *O. fasciatus* genome assembly based on SNP calling

S Table 8 Transcriptome data from RNA-seq for *O. fasciatus*

S Table 9 Gene annotation of the *O. fasciatus* genome

S Table 10 Functional annotation of the protein-coding genes in *O. fasciatus* genome

S Table 11 The annotation of non-coding RNAs of *O. fasciatus* genome

## Abbreviation

BUSCO: Benchmarking Universal Single-Copy Orthologs; NGS: Next Generation Sequencing; PacBio: Pacific Bioscience; SNP: single-nucleotide polymorphism; TE: transposable element.
