# Supplementary material for: Genome sequence of the barred knifejaw Oplegnathus fasciatus (Temminck & Schlegel, 1844): the first chromosome-level draft genome in the family Oplegnathidae
Source: Gigascience. 2019 Feb 1;8(3):giz013. doi: 10.1093/gigascience/giz013 (PMC6423371; doi:10.1093/gigascience/giz013)
Supplement: GIGA-D-18-00300_Original_Submission.pdf [file giz013_giga-d-18-00300_original_submission.pdf]

GigaScience

Genome sequence of rock bream, Oplegnathus fasciatus (Temminck & Schlegel, 1884): the first draft genome in family Oplegnathidae

--Manuscript Draft--

**Manuscript Number:**	GIGA-D-18-00300
**Full Title:**	Genome sequence of rock bream, Oplegnathus fasciatus (Temminck & Schlegel, 1884): the first draft genome in family Oplegnathidae
**Article Type:**	Data Note
**Funding Information:**	Shandong Province Key Research and Invention Program (2017GHY15102, 2017GHY15106) — Dr. Yongshuang Xiao<br>Young Scientists Fund (41506170) — Dr. Yongshuang Xiao
**Abstract:**	Background<br>The rock bream (Oplegnathus fasciatus), a member of the Oplegnathidae family of the Perciformes, is a commerically important rocky reef fish native to East Asia. O. fasciatus has become an important fishery resource for offshore cage aquaculture and fish stocking of marine ranching in China, Japan and Korea. Recently, growth of sexual dimorphism with neo-sex chromosome and widespread biotic diseases in O. fasciatus has received increasing concern. However, the adequate genome resources to make insight into sex-determining mechanism and to establish genetically basing resistant breeding systems for O. fasciatus have been lacking. Here, we performed whole genome of female fish for O. fasciatus using long-read sequencing and Hi-C data to generate chromosome-length scaffolds with highly contiguous genome assembly.<br><br>Findings<br>We assembled the O. fasciatus with a total of 245.0 Gb of raw reads, which were generated using both of PacBio Sequel and Illumina Hiseq 2000 platforms. The final draft genome assembly was approximately 778.7 Mb, which reached a remarkable high level of continuity with contig N50 of 2.1 Mb. The genome size was consistent with the estimated genome size (808.9 Mb) based on k-mer analysis. The identified repeat sequences account for 32.2% of the whole genome and 24 003 protein-coding genes with an average of 10.1 exons per gene were annotated using de novo method and with RNA-seq data and homologies to other teleosts. We combined Hi-C data with draft genome assembly to generate chromosome-length scaffolds. Twenty-four scaffolds corresponding to the twenty-four chromosomes were assembled to a final size of 768.8 Mb with contig N50 2.1 Mb and scaffold N50 of 33.5 Mb using 1372 contigs. According to the phylogenetic analysis using protein-coding genes, the O. fasciatus was close related to Larimichthys crocea and O. fasciatus diverged from their ancestor was at about 70.3-87.3 million years ago.<br><br>Conclusions<br>We generated high-quality draft genome and chromosomes assembly for O. fasciatus using long reads generated using PacBio sequencing technologies, which is the first reference genome for Oplegnathidae species. The genome assembly will provide insight into sex-determining mechanism and serve as a resource for accelerating the genome-assisted improvement of resistant breeding systems.
**Corresponding Author:**	Yongshuang Xiao<br><br>CHINA
**Corresponding Author Secondary Information:**	
**Corresponding Author's Institution:**	
**Corresponding Author's Secondary Institution:**	
**First Author:**	Yongshuang Xiao

Powered by Editorial Manager® and ProduXion Manager® from Aries Systems Corporation

**First Author Secondary Information:**	
**Order of Authors:**	Yongshuang Xiao
	Zhizhong Xiao
	Jing Liu
	Daoyuan Ma
	Jun Li
**Order of Authors Secondary Information:**	
**Additional Information:**	
**Question**	**Response**
Are you submitting this manuscript to a special series or article collection?	No
**Experimental design and statistics**<br><br>Full details of the experimental design and statistical methods used should be given in the Methods section, as detailed in our Minimum Standards Reporting Checklist. Information essential to interpreting the data presented should be made available in the figure legends.<br><br>Have you included all the information requested in your manuscript?	Yes
**Resources**<br><br>A description of all resources used, including antibodies, cell lines, animals and software tools, with enough information to allow them to be uniquely identified, should be included in the Methods section. Authors are strongly encouraged to cite Research Resource Identifiers (RRIDs) for antibodies, model organisms and tools, where possible.<br><br>Have you included the information requested as detailed in our Minimum Standards Reporting Checklist?	Yes
**Availability of data and materials**<br><br>All datasets and code on which the	Yes

conclusions of the paper rely must be either included in your submission or deposited in publicly available repositories (where available and ethically appropriate), referencing such data using a unique identifier in the references and in the “Availability of Data and Materials” section of your manuscript.<br><br>Have you have met the above requirement as detailed in our Minimum Standards Reporting Checklist?	

Powered by Editorial Manager® and ProduXion Manager® from Aries Systems Corporation

Manuscript

Click here to view linked References

Click here to access/download;Manuscript;2-MS-Genome sequence of rock bream, Oplegnathus fasciatus.docx

Genome sequence of rock bream, *Oplegnathus fasciatus* (Temminck & Schlegel, 1884): the first draft genome in family Oplegnathidae

Yongshuang Xiao[1,2,3, †], Zhizhong Xiao[1,2,3, †], Jing Liu[2,4*], Daoyuan Ma[1,2,3*], Jun Li[1,2,3*]

[1]CAS Key Laboratory of Experimental Marine Biology, Institute of Oceanology, Chinese Academy of Sciences, 7 Nanhai Road, Qingdao, 266071, China, [2] Laboratory for Marine Biology and Biotechnology, Qingdao National Laboratory for Marine Science and Technology, 7 Nanhai Road, Qingdao, 266071, China, [3]Center for Ocean Mega-Science, Chinese Academy of Sciences, 7 Nanhai Road, Qingdao, 266071, China.

∗Correspondence address: Jing Liu, Institute of Oceanology, Chinese Academy of Sciences, 7 Nanhai Road, Qingdao, 266071, China; Tel: +86-053282898790; E-mail: jliu@qdio.ac.cn; Daoyuan Ma, Mega-Science, Chinese Academy of Sciences, 7 Nanhai Road, Qingdao, 266071, China; Tel: +86-053282898717; E-mail: madaoyuan1@163.com; Jun Li, Institute of Oceanology, Chinese Academy of Sciences, 7 Nanhai Road, Qingdao, 266071, China; Tel: +86-053282898718; E-mail: junli@qdio.ac.cn.

†Contributed equally to this work.

Abstract

Background

The rock bream (*Oplegnathus fasciatus*), a member of the Oplegnathidae family of the Perciformes, is a commerically important rocky reef fish native to East Asia. *O. fasciatus* has become an important fishery resource for offshore cage aquaculture and fish stocking of marine ranching in China, Japan and Korea. Recently, growth of sexual dimorphism with neo-sex chromosome and widespread biotic diseases in *O. fasciatus* has received increasing concern. However, the adequate genome resources to make insight into sex-determining mechanism and to establish genetically basing resistant breeding systems for *O. fasciatus* have been lacking. Here, we performed whole genome of female fish for *O. fasciatus* using long-read sequencing and Hi-C data to generate chromosome-length scaffolds with highly contiguous genome assembly.

Findings

We assembled the *O. fasciatus* with a total of 245.0 Gb of raw reads, which were generated using both of PacBio Sequel and Illumina Hiseq 2000 platforms. The final draft genome assembly was approximately 778.7 Mb, which reached a remarkable high level of continuity with contig N50 of 2.1 Mb. The genome size was consistent with the estimated genome size (808.9 Mb) based on *k*-mer analysis. The identified repeat sequences account for 32.2% of the whole genome and 24 003 protein-coding genes with an average of 10.1 exons per gene were annotated using *de novo* method and with RNA-seq data and homologies to other teleosts. We combined Hi-C data with draft genome assembly to generate chromosome-length scaffolds. Twenty-four scaffolds corresponding to the twenty-four chromosomes were assembled to a final size of 768.8 Mb with contig N50 2.1 Mb and scaffold N50 of 33.5 Mb using 1372 contigs. According to the phylogenetic analysis using protein-coding genes, the *O. fasciatus* was close related to *Larimichthys crocea* and *O. fasciatus* diverged from their ancestor was at about 70.3-87.3 million years ago.

Conclusions

We generated high-quality draft genome and chromosomes assembly for *O. fasciatus*

using long reads generated using PacBio sequencing technologies, which is the first reference genome for Oplegnathidae species. The genome assembly will provide insight into sex-determining mechanism and serve as a resource for accelerating the genome-assisted improvement of resistant breeding systems.

Keywords: *Oplegnathus fasciatus*; genome assembly; Hi-C assembly; sex-determining mechanism

Data description

Introduction of *O. fasciatus*

The family Oplegnathidae belongs to the order Perciformes, including only one genus *Oplegnathus* comprised of two species, *O. fasciatus* and *O. punctatus* that are of commercial values. The rock bream, *O. fasciatus* (Temminck & Schlegel, 1844), is one of the two species in the *Oplegnathus*, which is commonly found at the depth of one to ten meters in association with rocky reefs[1, 2], being distributed in a wide range of shallow waters around Korea, Japan, China and Hawaii[1, 3, 4] (Fig. 1). *O. fasciatus* has become an important fishery resource for offshore cage aquaculture and fish stocking of marine ranching in China, Japan and Korea[5]. It was reported that the male of *Oplegnathus* has a neo-sex chromosome, possibly a sex chromosome Y, and the sex chromosome system for *Oplegnathus* was considered to be $X_1 X_1 X_2 X_2$ / $X_1 X_2 Y$ based on the karyotype analyses[6, 7]. Furthermore, the growth sexual dimorphism was detected in the *O. fasciatus* and the male fish showed a faster growth advantage than the female, may be due to the sex chromosome system of *Oplegnathus*[8]. *O. fasciatus* is vulnerable to viruses (eg. Iridovirus) and genetic degradation caused by inbreeding has led to higher susceptibility to diseases[9, 10]. It is vital to develop genomic resources for making insight into sex-determining mechanism and accelerating the genome-assisted improvement of resistant breeding systems.

So far, the genome sequence and the chromosomes assembly of *O. fasciatus* have not been reported. Here we performed a high-quality reference genome assembly for *O. fasciatus* constructed using long reads by the PacBio DNA sequencing platform, and using a genome assembly strategy by taking advantage of genome assemblyer

Canu[11]. The genome assembly of *O. fasciatus* is the first reference genome constructed for the family Oplegnathidae. The completeness and continuity of the genome will provide high quality genomic resources for studies on sex-determining mechanism and for accelerating the genome-assisted improvement of resistant breeding systems.

Genomic DNA extraction, genome size estimation and Hi-C library construction

High-quality genomic DNA for Illumina platform (Illumina Inc., San Diego, CA, USA) and PacBio Sequel sequencing (Pacific Biosciences of California, Menlo Park, CA, USA) was extracted from fresh muscle tissue and blood sample of a single female *O. fasciatus*. The fish was collected from the near-shore area of Qingdao city (Yellow Sea), Shandong province. A whole-genome using Illumina DNA sequencing technology was applied to estimate *O. fasciatus* genome size. A short-insert library (300~350 bp) was constructed and generated a total of ~90.7 Gb of raw reads using the standard protocol provided by Illumina Hieq X Ten platform (Illumina Inc., San Diego, CA, USA). After removal of low-quality and redundant reads, we obtained about ~80.8 Gb of clean data for *de novo* assembly to estimate the genome size (S Table 1, Fig. 2). All the cleaned reads were subjected to 17-mer frequency distribution analysis[12]. As the total number of *k*-mers was about 8.09×10^{10} and the peak of *k*-mers at a depth of 100, the genome size of *O. fasciatus* was calculated to be 808.9 Mb using the following formula: genome size = *k*-mer number / peak depth (Fig. 2). Meanwhile, the estimated heterozygosity of 0.29% and a repeat content of 38.46% were detected for *O. fasciatus* in this work. A pilot genome assembly was approximately 808.9 Mb with a contig N50 7.2 kb and scaffold N50 84.1kb using the Illumina data and the assembly program Platanus package[13] (S Table 2). The GC content was 41% (S Fig. 1). This genome assembly was of low-quality partly due to its high genomics repeat content.

The genomic DNA for Hi-C library was extracted from the whole-blood cell of *O. fasciatus* as described[14]. The cells were fixed with formaldehyde and lysed, and the cross-linked DNA digested with MboI. Sticky ends were biotin-labeled and proximity

ligated to form chimeric junctions that were enriched for and then physically sheared to a size of 300–500 bp[14]. Chimeric fragments representing the original cross-linked long-distance physical interactions were then processed into paired-end sequencing libraries, and 629 million 150-bp paired-end Illumina reads (91.5 Gb) with Q20 and Q30 of ~94.0% were produced (S Table 1, S Table 3). As a result, the paired data, data with mate mapped to a different contig (or scaffold) and data with mapped to a different contig (or scaffold) (map Q5⩾ 5) were 593.7 Mb (94.4%), 240.5 Mb (40.5%) and 205.1 Mb (34.6%), respectively (S Table 3).

Genome assembly using PacBio long reads

Two 20 kb genomic DNA libraries were constructed and sequenced using PacBio Sequel platform, generating 62.9 Gb raw DNA reads. We obtain 4.8 million subreads (totally 62.8 Gb) with a read N50 length of ~22 kb after removing adaptor (S Table 1).

The Canu v1.4 was firstly used to assemble the genome with the Corrected-Error-Rate parameter set at 0.040[11]. As a result, a total length of 875.9 Mb genome assembly was achieved for *O. fasciatus,* which was consistent with the estimated genome size in 17-mer analysis based on the Illumina data (S Table 2). We applied Redundans v0.13c[15] to remove the sequence redundancy and obtain genome assembly size of 778.0 Mb. We then used the Arrow of Smrtlink 5.0 with the minCoverage parameter set at 15 to implement the error correction based on the PacBio long reads data (Table 1). The resulting genome assembly was further polished using NGS data, which were used in the genome survey analysis above. The final draft genome assembly was 778.7 Mb, which reached a remarkable high level of continuity with contig N50 length of 2.1 Mb (Table 1). The contig N50 of *O. fasciatus* was much higher than those of previous fish genome assemblies constructed using NGS DNA sequencing technologies, and is comparable with those of recently reported model fish species (S Table 4)

Genome quality evaluation

To assess the completeness of the assembled *O. fasciatus* genome, we subjected the sequences to BUSCO version 3 evaluation (BUSCO, actinopterygii_odb9) [16]. Overall, 96.6% and 1.5 % of the 4 584 expected actinopterygii genes were identified in the assembled genome as complete and partial BUSCO profiles, respectively. Approximately 85 genes could be considered missing in our assembly (S Table 5). Among the expected complete actinopterygii genes, both of 4 259 and 171 were identified as single copy and duplicated BUSCOs, respectively (S Table 5). We then used the Minimap2 to estimate the completeness and homogeneity of genome assembly based on the CLR (Continuous Long Reads) subreads. A high quality of completeness and homogeneity was checked for genome assembly, and the mapping rate, coverage rate and average sequencing depth were reached to 90.2%, 99.9% and 80.6, respectively (S Table 6).

To further evaluate the accuracy of *O. fasciatus* genome assembly, we aligned the NGS-based short reads from whole-genome sequencing data against the reference genome using BWA[17]. We then used the GATK to implement the SNP calling and filter work, the result showed 99.8% and 0.2 % of the 1.6 x 10^6 expected SNP reads were identified in the assembled genome as heterozygosis and homology SNPs, respectively. SNP calling on the final assembly also yield a heterozygosity rate of 0.20%, supporting the estimate from *k*-mer analysis (0.29%) (S Table 7).

Repeat sequence within the *O. fasciatus* genome assembly

To identify tandem repeats, we utilized Tandem Repeat Finder to annotate repetitive elements in the *O. fasciatus* genome. RepeatModeler (version 1.04) and LTR_FINDER[18] were used to construct a *de novo* repeat library with default parameters. Subsequently, we used RepreatMasker[19] (version 3.2.9) to map our assembled sequences on the Repbase TE (version 14.04) [20] and the *de novo* repeat library to identify known and novel transposable elements (TEs). In addition, the TE-related proteins were annotated by using RepeatProteinMask software (version 3.2.2) [19].

The total identified repeat sequences accounted for 23.6% of the *O. fasciatus*

genome based on the *de novo* repeat library (Table 2). Approximately 23.41% of the *O. fasciatus* genome was identified as interspersed repeats (most often TEs). Among them, DNA transposable elements were the most abundant type of repeat sequences, which occupied 11.5% of the whole genome. The long interspersed nuclear elements (LINE) and long terminal repeat (LTR) took up 7.3% and 4.0% of the whole genome (Table 2, S Fig. 2).

RNA preparation and sequencing

We sequenced cDNA libraries prepared from the eggs of *O. fasciatus* used for genome annotation using Illumina sequencing technologies. High quality of RNA were detected based on the estimation of the absorbance at 260nm / 280nm (OD = 2.0) and the RIN (value = 9.2) by Nanodrop ND-1000 spectrophotometer (LabTech, USA) and 2100 Bioanalyzer (Agilent Technologies, USA), respectively. We used the Clontech SMARTer cDNA synthesis kit to complete the process of reverse transcription. The paired-end library was prepared following the manual of the Paired-End Sample Preparation Kit (Illumina Inc., San Diego, CA, USA). Finally, the library with an insert length of 300 bp was sequenced by Illumina HiSeq X Ten in 150PE mode (Illumina Inc., San Diego, CA, USA). As a result, we obtained ~42.2 Gb high-quality transcriptome data from RNA-seq (S Table 1, S Table 8)

Gene annotation

Gene annotation of the *O. fasciatus* genome was performed using *de novo*, homology-based and transcriptome sequencing-based prediction. We employed Augustus (version 2.5.5)[21] and GenScan (version 1.0)[22] softwares to predict protein-coding genes of *O. fasciatus* genome assembly. Protein sequences of closely related fish species including *Larimichthys crocea*，*Lates calcarifer*，*Gasterosteus aculeatus*，*Paralichthys olivaceus*，*Cynoglossus semilaevis* and *Gadus morhua* were downloaded from Ensembl[23] and aligned against to *O. fasciatus* genome using TBLASTN software[24]. Subsequently, Genewise2.2.0 software[25] was employed to predict the potential gene structures on all alignments.

We also mapped these NGS transcriptome short reads onto our genome assembly using TopHat1.2 software[26], and then we employed Cufflinks[27] to predict the gene

structures (S Table 9). All gene models were then integrated using MAKER to obtain a consensus gene set[28]. The final total gene set was composed of 24 003 genes, with an average of 10.1 exons per gene in *O. fasciatus* genome (Table 1). The gene number, gene length distribution, CDS length distribution, exon length distribution and intron length distribution were all comparable with those in other teleost fish species (S Table 9, S Fig. 3).

In order to further obtain functional annotation of the protein-coding genes in *O. fasciatus* genome, we employed local BLASTX and BLASTN programs to align upon the non-redundant protein (NR), non-redundant nucleotide (NT) and Swissprot database with an e-value ⩽ 1e-5[29]. We also used Blast2GO software to search the Gene ontology (GO), Kyoto Encyclopedia of Genes and Genomes (KEGG) pathway database[30, 31, 32]. Finally, 97.3% (23 364 genes) of the 24 003 genes were annotated by at least one database (S Table10). Four types of non-coding RNAs (microRNAs, transfer RNAs, ribosomal RNAs, and small nuclear RNAs) were also annotated using tRNAscan-SE and the Rfam database in this study[33, 34] (S Table11).

Hi-C assembly and chromosome interactions

Hi-C was a sequencing-based approach for determining chromosome interactions by calculating the contact frequency between pairs of loci, which depended strongly on the one-dimensional distance, in base pairs, between a pair of loci[35, 36]. We employed BWA and Lachesis softwares to align paired-end reads to the draft genome assembly and filtered all base sequences other than 500bp from each restriction site[37]. According to the conduct of clustering, ordering, and orienting to the assembly contigs (1 692), those were grouped into 24 chromosome clusters and scaffolded using Lachesis software with tuned parameters[38] (Table 3, Fig. 3). Finally, we constructed the chromosome interactions map using Juicer software and employed the JucieBox to complete the visual correction of interactions map. We obtained polished 1 756 contigs by interrupting misassembly from the 1 692 contigs. Twenty-four scaffolds corresponding to the 24 chromosomes of *O. fasciatus* based on the karyotype analyses were assembled[6, 7] (Table 3, Fig. 3). A final size of 768.8 Mb accounting for the 98.7% draft genome was assembled, which remarkable high level

of continuity with contig N50 of 2.1 Mb and scaffold N50 of 33.5 Mb using 1372 contigs. The anchor rate of contigs (> 100 kb) to chromosomes was reached up to the 99.7% based on the Hi-C assembly (Table 4). The contig N50 and scaffold N50 of *O. fasciatus* were much higher than those of previous fish genome assemblies constructed using NGS DNA sequencing technologies based on the genome assembly using PacBio long reads and Hi-C assembly (S Table 4).

Gene family identification and phylogenetic tree construction

We employed the BLASTP program[39] with an e-value threshold of 1e-5 to identify gene family based on the transcripts alignments of each gene from *O. fasciatus* and other fish species, which included *Larimichthys crocea*, *Gadus morhua*, *Paralichthys olivaceus*, *Cynoglossus semilaevis*, *Notothenia coriiceps*, *Boleophthalmus pectinirostris*, *Branchiostoma floridae*, *Gasterosteus aculeatus*, *Callorhinchus milii*, *Danio rerio*, *Salmo salar* and *Oryzias latipes*. 23273 gene families were identified by clustering of homologous gene sequences based on H-scores calculated from Bit-score in Hcluster_sg software (S Fig. 4). Subsequently, we selected 812 single-copy orthogroups from the above-mentioned species to construct the phylogenetic relationship between *O. fasciatus* and the other fish species. We used the Clustal W program[40] to extract and align coding sequences of single-copy gene from the 765 orthogroups with length filter, respectively (S Fig. 5). All the alignments were concatenated as a single data set for each species. Nondegenerated sites extracted from the data set were then joined into new sequence of each species to construct a phylogenetic tree based on the maximum-likelihood method implemented in the PhyML package[41] (with the -m PROTGAMMAAUTO model). We used the MCMCtree program to estimate divergence times among species based on the approximate likelihood method[42] and a molecular clock data from the divergence time between medaka from the TimeTree database[43]. According to the phylogenetic analysis *O. fasciatus* were clustered together with *Larimichthys crocea* belonged to the order Perciformes, which was consistent with the fish species taxonomy. The taxonomy of Notothenioidei should be elevated to the order level from the Perciformes and be paralleled with Gasterosteiformes (Fig. 4). The divergence time

between *O. fasciatus* and the common ancestor with *Larimichthys crocea* was at about 70.3-87.3 Ma.

Conclusion

We successfully assembled the genome of *O. fasciatus* and reported the first whole genome sequencing, assembly and annotation based on long reads from the third-generation PacBio Sequel sequencing platform. The final draft genome assembly is approximately 778.7 Mb, accounting for 96.3% of the estimated genome size (808.9 Mb) based on *k*-mer analysis. The genome assembly of *O. fasciatus* was also the first high-quality genome of all species in Oplegnathidae family, which reached a remarkable high level of continuity with contig N50 of 2.1 Mb and scaffold N50 of 33.5 Mb. The contig N50 was remarkably longer than those of most fish genome assemblies, and was comparable with those of recently reported model fish species. We also predicated 24 003 protein-coding genes from the generated assembly, and 97.3% (23 364 genes) of all protein-coding genes were annotated. Twenty-four scaffolds corresponding to the twenty-four chromosomes were assembled to a final size of 768.8 Mb using 1372 contigs based on the Hi-C assembly. We found the taxonomy of Notothenioidei should be elevated to the order level and the divergence time between *O. fasciatus* and the common ancestor with *Larimichthys crocea* was at about 70.3-87.3 Ma. The genome assembly, together with gene annotation data generated in this work provided a valuable resource for research on sex-determining mechanism and for accelerating the genome-wide association studies on resistant breeding systems.

Ethics Statement

This research was approved by the Animal Care and Use committee of Chinese Academic Science. All participates consent the study under the 'Ethics, consent and permissions' heading. All participants consent to publish the work under the 'Consent to publish' heading.

Availability of supporting data

Supporting data and materials are available in the GigaScience GigaDB database, with the raw sequences deposited in the SRA under the accession number SRP158313.

Competing interests

The authors declare that they have no competing interests.

Funding

This study was supported by a grant from the National Natural Science Foundation of China (No. 41506170, No. 31672672, and No. 31872195), Shandong Province Key Research and Invention Program (2017GHY15102, 2017GHY15106), Qingdao Source Innovation Program (17-1-1-57-jch), Marine Fishery Institute of Zhejiang Province, Key Laboratory of Mariculture and Enhancement of Zhejiang Province (2016KF002). Qingdao National Laboratory for Marine Science and Technology (2015ASKJ02, 2015ASKJ02-03-03), STS project (KFZD-SW-106, ZSSD-019).

Author Contributions

YSX conceived the project. ZZX, DYM collected the samples and extracted the genomic DNA. YSX, JL and JL performed the genome assembly and data analysis. YSX, ZZX, JL, DYM and JL wrote the paper.

Reference

1. Schembri, P.J. *et al*. Occurrence of barred kinfejaw, *Oplegnathuf fasciatus* (Actinopterygii: Perciformes: Oplegnathidae), in Malta (Central Mediterranean) with a discussion on possible modes of entry. *Acta Ichthyol Piscat* 40,101-104 (2010).

2. Mundy, B.C. Checklist of the fishes of the Hawaiian Archipelago. *Bishop Mus Bull Zool* 6, 1-704 (2005).

3. An, H.S. & Hong, S.W. Genetic diversity of rock bream *Oplegnathus fasciatus* in Southern

Korea. *Genes Genom* 30, 451-459 (2008).

4. Xiao, Y.S. *et al.* Pronounced population genetic differentiation in the rock bream *Oplegnathus fasciatus* inferred from mitochondrial DNA sequences. *Mitochondrial DNA A* 27, 2045-2052 (2016).

5. Park, H.S. *et al.* Population Genetic Structure of Rock Bream (*Oplegnathus fasciatus* Temminck & Schlegel, 1884) Revealed by mtDNA COI Sequence in Korea and China. Ocean Sci J 53, 261-274 (2018).

6. Xu, D.D. *et al*. Chromosomal mapping of microsatellite repeats in the rock bream fish *Oplegnathus fasciatus*, with emphasis of their distribution in the neo-Y chromosome. *Mol Cytogenet* 6, 12 (2013).
7. Xue, R. *et al.* Karyotype and Ag-Nors In Male And Female Of *Oplegnathus Punctatus*. *Oceanol Limnol Sin* 47, 626-632 (2016).
8. Xiao, Z.Z. Study on population genetics and culture biology of *Oplegnathus fasciatus*. Doctor thesis p 162-176 (2015).
9. Zhang, B.C. *et al*. Rock bream (*Oplegnathus fasciatus*) viperin is a virus-responsive protein that modulates innate immunity and promotes resistance against megalocytivirus infection. *Dev Comp Immunol* 45, 35-42 (2014).
10. L, H. *et al*. Characterization of an Iridovirus Detected in Rock Bream (Oplegnathus fasciatus ;Temminck and Schlegel). *Chin J Virol* 27, 158-164 (2011).
11. Koren, S. *et al.* Canu: scalable and accurate long-read assembly via adaptive k-mer weighting and repeat separation. *Genome Res* 27, 722 (2017).
12. Marcais, G. & Kingsford, C. A fast, lock-free approach for efficient parallel counting of occurrences of k-mers. *Bioinformatics* 27, 764–70 (2011).
13. Kajitani, R. *et al.* Efficient de novo assembly of highly heterozygous genomes from whole-genome shotgun short reads. *Genome Res* 24, 1384-1395 (2014).
14. Sandborn, A.L. *et al*. Chromatin extrusion explains key features of loop and domain formation in wild-type and engineered genomes. *Proc Natl Acad Sci USA* 112, E6456 (2015).
15. Pryszcz, L. P. & Gabaldón, T. Redundans: an assembly pipeline for highly heterozygous

genomes. *Nucleic Acids Res* 44, e113-e113 (2016).

16. Simão, F. A. *et al*. BUSCO: assessing genome assembly and annotation completeness with single-copy orthologs. *Bioinformatics* 31, 3210 (2015).
17. Li, H. & Durbin, R. Fast and accurate short read alignment with Burrows–Wheeler transform *Bioinformatics* 25, 1754-1760 (2009).
18. Benson, G. Tandem repeats finder: a program to analyze DNA sequences. *Nucleic Acids Res* 27, 573–80 (1999).
19. Tarailo-Graovac, M. & Chen, N. Using RepeatMasker to identify repetitive elements in genomic sequences. In: Editoral board, Baxevanis Andreas D et al. (eds.), Current Protocols in Bioinformatics, Chapter 4:Unit 4 10 (2009).
20. Jurka, J. *et al.* Repbase Update, a database of eukaryotic repetitive elements. Cytogenetic Genome Res 110, 462–67 (2005).
21. Stanke, M. *et al*. AUGUSTUS: a web server for gene finding in eukaryotes. *Nucleic Acids Res* 32(Web Server issue), W309-12 (2004).
22. Cai, Y. *et al.* Computational systems biology methods in molecular biology, chemistry biology, molecular biomedicine, and biopharmacy. *BioMed Res Int* 2014, 746814 (2014).
23. Flicek, P. *et al*. Ensembl 2014. *Nucleic Acids Res* 42, D749-D755 (2014).
24. Gertz, E. M. *et al.* Composition-based statistics and translated nucleotide searches: Improving the TBLASTN module of BLAST. *MBC Biology* 4, 41 (2006).
25. Birney, E. et al. GeneWise and Genomewise. *Genome Res* 14, 988-995 (2004).
26. Trapnell, C. *et al.* TopHat: discovering splice junctions with RNA-Seq. *Bioinformatics* 25, 105-1111 (2009).
27. Ghosh, S. & Chan, C. K. K. Analysis of RNA-Seq data using TopHat and Cufflinks. *Methods Mole Biol* 1374, 339 (2016).
28. Campbell, M. S. *et al*. Genome Annotation and Curation Using MAKER and MAKER-P. *Current Protocols in Bioinformatics* 48, 4.11.11 (2014).
29. Lobo, I. Basic Local Alignment Search Tool (BLAST). *J Mol Biol* 215, 403-410 (2008).
30. Harris, M. A. *et al.* The Gene Ontology (GO) database and informatics resource. *Nucleic Acids Res* (2004).
31. Ogata, H. *et al.* KEGG: Kyoto Encyclopedia of Genes and Genomes. *Nucleic Acids Res* 27,

29-34 (2000).

32. Conesa, A. *et al.* Blast2GO: a universal tool for annotation, visualization and analysis in functional genomics research. *Bioinformatics* 21, 3674 (2005).

33. Lowe, T. M. & Eddy, S. R. tRNAscan-SE: a program for improved detection of transfer RNA genes in genomic sequence. *Nucleic Acids Res* 25, 955-964 (1997).

34. Griffithsjones, S. *et al.* Rfam: an RNA family database. *Nucleic Acids Res* 31, 439 (2003).

35. Lieberman-Aiden, E. *et al*. Comprehensive mapping of long-range interactions reveals folding principles of the human genome. *Science* 326, 289-293 (2009).

36. Rao, S.S.P. *et al.* A 3D map of the human genome at kilobase resolution reveals principles of chromatin looping. *Cell* 159, 1665-1680 (2014).

37. Flot, J.F. *et al*. Contact genomics: scaffolding and phasing (meta) genomes using chromosome be 3D physical signatures. *FEBs Letters* 589, 2966-2974 (2015).

38. Burton, J.N. *et al*. Chromosome-scale scaffolding of de novo genome assemblies based on chromatin interactions. *Nat Biotechnol* 31, 1119-1125 (2013).

39. Lobo, I. Basic Local Alignment Search Tool (BLAST). J Mol Biol 215, 403-410 (2008).

40. Thompson, J. D. *et al.* Multiple sequence alignment using ClustalW and ClustalX. *Curr Protoc Bioinformatics* 2.3. 1-2.3. 22 (2002).

41. Guindon, S. *et al.* PhyML: Fast and Accurate Phylogeny Reconstruction by Maximum Likelihood. *Infect Genet Evol* 9, 384-385 (2009).

42. Yang, Z. & Rannala, B. Bayesian estimation of species divergence times under a molecular clock using multiple fossil calibrations with soft bounds. *Mol Biol Evol* 23, 212-226 (2006).

43. Hedges, S. B. *et al.* Tree of life reveals clock-like speciation and diversification. *Mol Biol Evol* 32, 835-845 (2015).

Table Click here to access/download;Table;2-Tables.docx

Table 1 Summary of *Oplegnathus fasciatus* genome assembly and annotation

Genome assembly	values
Contig N50 size (Mb)	2.1
Contig number	1 692
Scaffold N50 size (Mb)	33.5
Scaffold N50 number	24
Total length (Mb)	778.7
Genome coverage (X)	314.6
Contig number (≥ 1 Mb)	219
Length of contig (≥ 1 Mb) (bp)	565 184 128
The longest contig (bp)	8 891 851
The longest scaffold (bp)	38 619 456
Genome annotation	
Protein-coding gene number	24 003
Mean transcript length (kb)	16.1
Mean exons per gene	10.1
Mean exon length (bp)	217.7
Mean intron length (bp)	1527.4

Table 2 The detailed classification of repeat sequences of *Oplegnathus fasciatus*

Type	Repbase TEs		TE proteins		De novo		Combined TEs	
	Length (bp)	% in genome	Length (bp)	% in genome	Length (bp)	% in genome	Length (bp)	% in genome
DNA	39 147 527	5.03	5 390 266	0.69	93 089 344	11.95	124 417 402	15.98
LINE	23 983 322	3.08	16 460 762	2.11	57 167 551	7.34	85 761 250	11.01
SINE	875 585	0.11	0	0.00	914 559	0.12	1 747 250	0.22
LTR	10 163 601	1.31	5 770 483	0.74	31 126 639	4.00	42 465 968	5.45
Satellite	2 028 992	0.26	0	0.00	2 613 480	0.34	4 361 048	0.56
Simple_repeat	1 556 026	0.20	0	0.00	5 179 965	0.67	6 386 303	0.82
Other	6 545	0.00	0	0.00	0	0.00	6 545	0.00
Unknown	331 430	0.04	0	0.00	20 636 768	2.65	20 967 052	2.69
Total	73 544 786	9.44	27 613 880	3.55	183 954 095	23.62	250 611 845	32.18

Table 3 Hi-C libraries for chromosome-scale assembly of *Oplegnathus fasciatus*

Chromosome	Number of contigs	Length of contigs	Length of chromosome
Chr1	36	19 852 463	19 869 963
Chr2	51	34 905 999	34 930 999
Chr3	43	33 654 321	33 675 321
Chr4	74	35 290 762	35 327 262
Chr5	54	38 592 956	38 619 456
Chr6	72	38 156 734	38 192 234
Chr7	60	35 029 969	35 059 469
Chr8	64	37 546 719	37 578 219
Chr9	45	31 457 603	31 479 603
Chr10	52	35 302 682	35 328 182
Chr11	80	31 971 344	32 010 844
Chr12	46	30 287 574	30 310 074
Chr13	52	33 665 353	33 690 853
Chr14	101	31 190 130	31 240 130

Chr15	48	30 038 946	30 062 446
Chr16	59	28 825 591	28 854 591
Chr17	33	28 220 078	28 236 078
Chr18	50	26 754 155	26 778 655
Chr19	52	34 380 882	34 406 382
Chr20	52	25 675 509	25 701 009
Chr21	64	31 397 692	31 429 192
Chr22	63	30 492 179	30 523 179
Chr23	70	33 514 462	33 548 962
Chr24	51	31 930 140	31 956 140
Total	1 372	768 134 243	768 808 243

Table 4 Genome assembly of *Oplegnathus fasciatus* based on chromosome-length scaffolds

	Draft scaffolds	Chromosome-length scaffolds based on Hi-C
Length of genome (bp)	778 731 089	768 808 243
Number of contigs	1 692	1 372
Contigs N50 (bp)	2 149 025	2 130 780
Number of scaffold	/	24
Scaffold N50 (bp)	/	33 548 962
Number of contigs (≥ 100 kb)	693	708
Total length of contigs (≥ 100 kb)	735 235 962	732 827 446
Mapping rate of contigs (≥ 100 kb) (%)	/	99.67

Figure Legends

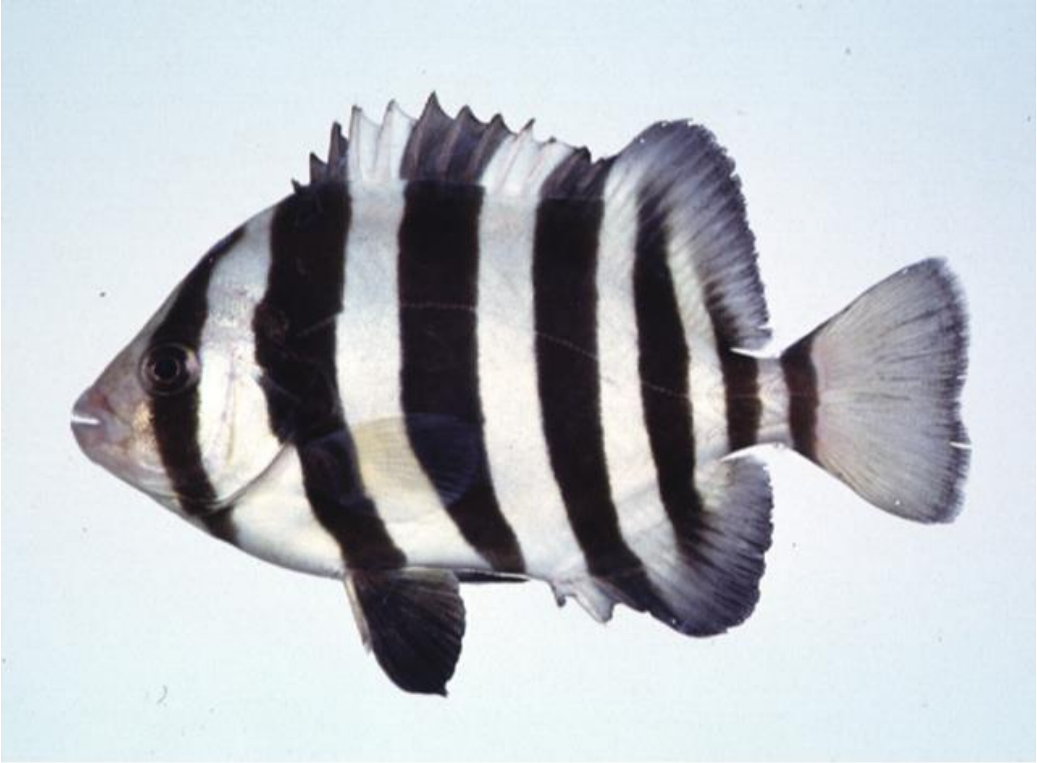

Fig. 1 A representative individual of *O. fasciatus*

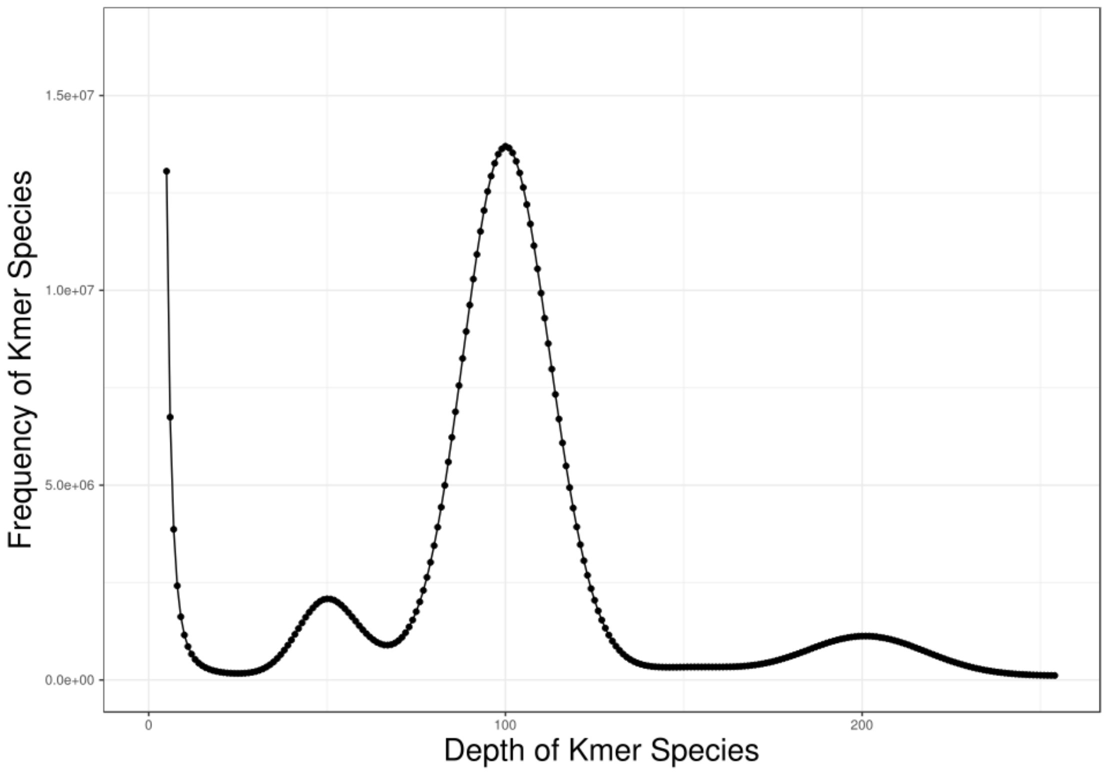

Fig. 2 *k*-mer distribution of the *O. fasciatus* genome

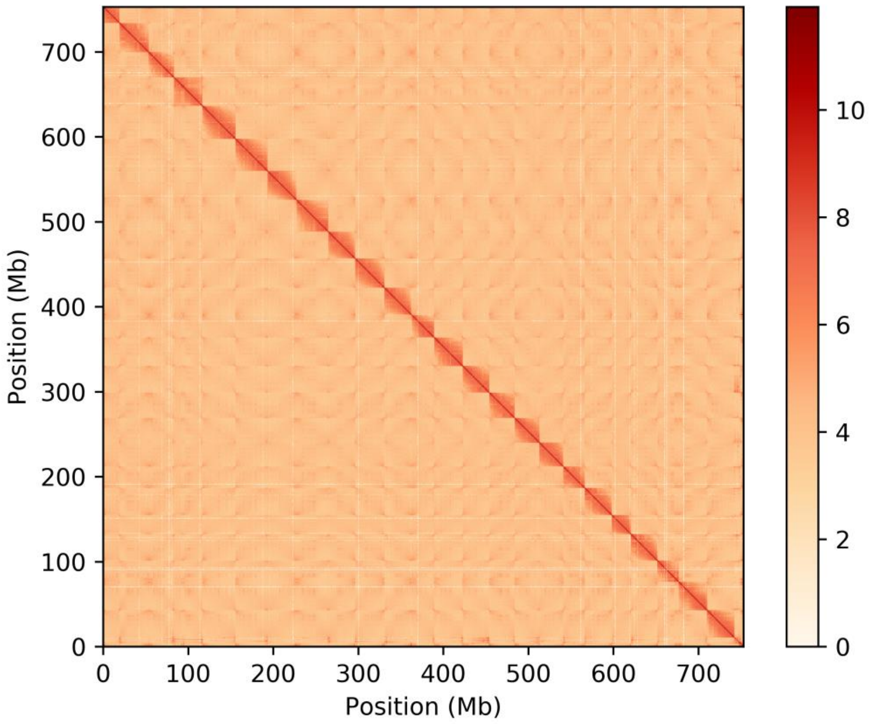

Fig. 3 Hi-C interaction heatmap for *O. fasciatus* reference genome, showing interactions between the 24 chromosomes

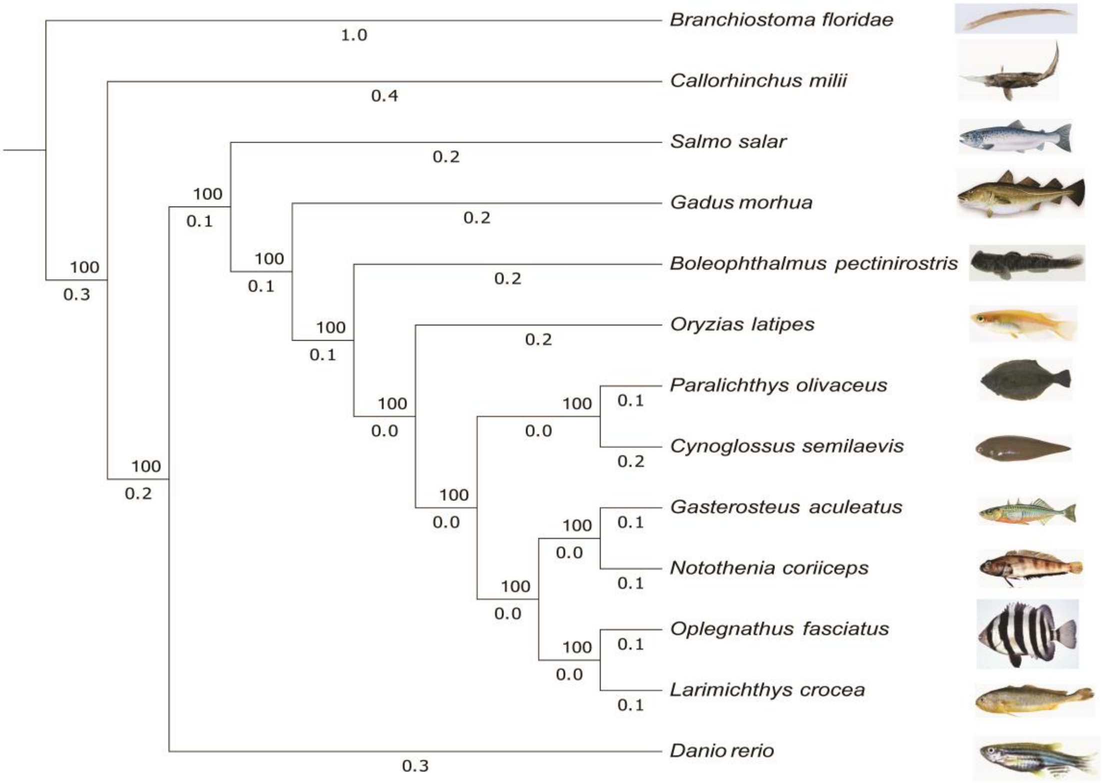

Fig. 4 The phylogenetic relationships of *O. fasciatus* with other fishes

Click here to access/download
Supplementary Material
4-supplementary materials.docx
