# Supplementary material for: Genome sequence of the barred knifejaw Oplegnathus fasciatus (Temminck & Schlegel, 1844): the first chromosome-level draft genome in the family Oplegnathidae
Source: Gigascience. 2019 Feb 1;8(3):giz013. doi: 10.1093/gigascience/giz013 (PMC6423371; doi:10.1093/gigascience/giz013)
Supplement: GIGA-D-18-00300_Revision_1.pdf [file giz013_giga-d-18-00300_revision_1.pdf]

GigaScience

Genome sequence of the barred knifejaw Oplegnathus fasciatus (Temminck & Schlegel, 1884): the first chromosome-level draft genome in the family Oplegnathidae

--Manuscript Draft--

**Manuscript Number:**	GIGA-D-18-00300R1
**Full Title:**	Genome sequence of the barred knifejaw Oplegnathus fasciatus (Temminck & Schlegel, 1884): the first chromosome-level draft genome in the family Oplegnathidae
**Article Type:**	Data Note
**Funding Information:**	Shandong Province Key Research and Invention Program (2017GHY15102, 2017GHY15106) — Dr. Yongshuang Xiao<br>Young Scientists Fund (41506170) — Dr. Yongshuang Xiao
**Abstract:**	see below
**Corresponding Author:**	Yongshuang Xiao<br><br>CHINA
**Corresponding Author Secondary Information:**	
**Corresponding Author's Institution:**	
**Corresponding Author's Secondary Institution:**	

Background
The barred knifejaw (Oplegnathus fasciatus), a member of the Oplegnathidae family of the Centrarchiformes, is a commercially important rocky reef fish native to East Asia. O. fasciatus has become an important fishery resource for offshore cage aquaculture and fish stocking of marine ranching in China, Japan and Korea. Recently, sexual dimorphism in growth with neo-sex chromosome and widespread biotic diseases in O. fasciatus has been received increasing concern. However, adequate genome resources for gaining insight into sex-determining mechanisms and establishing genetically resistant breeding systems for O. fasciatus are lacking. Here, we analysed the entire genome of a female O. fasciatus fish using long-read sequencing and Hi-C data to generate chromosome-length scaffolds and a highly contiguous genome assembly.

Findings
We assembled the O. fasciatus genome with a total of 245.0 Gb of raw reads that were generated using both of PacBio Sequel and Illumina HiSeq 2000 platforms. The final draft genome assembly was approximately 778.7 Mb, which reached a high level of continuity with a contig N50 of 2.1 Mb. The genome size was consistent with the estimated genome size (777.5 Mb) based on k-mer analysis. We combined Hi-C data with a draft genome assembly to generate chromosome-length scaffolds. Twenty-four scaffolds corresponding to the twenty-four chromosomes were assembled to a final size of 768.8 Mb with a contig N50 of 2.1 Mb and a scaffold N50 of 33.5 Mb using 1,372 contigs. The identified repeat sequences accounted for 33.9% of the entire genome, and 24,003 protein-coding genes with an average of 10.1 exons per gene were annotated using de novo methods, with RNA-seq data and homologies to other teleosts. According to phylogenetic analysis using protein-coding genes, O. fasciatus is closely related to Larimichthys crocea, with O. fasciatus diverging from their common ancestor approximately 70.5-88.5 million years ago.

Conclusions
We generated a high-quality draft genome with chromosome assembly for O. fasciatus using long reads by using the PacBio sequencing technologies, which represents the first chromosome-level reference genome for Oplegnathidae species. Assembly of this genome will provide insight into sex-determining mechanisms and serve as a resource for accelerating genome-assisted improvements in resistant breeding systems.

Powered by Editorial Manager® and ProduXion Manager® from Aries Systems Corporation

**First Author:**	Yongshuang Xiao
**First Author Secondary Information:**	
**Order of Authors:**	Yongshuang Xiao
	Zhizhong Xiao
	Jing Liu
	Daoyuan Ma
	Jun Li
**Order of Authors Secondary Information:**	
**Response to Reviewers:**	GIGA-D-18-00300<br>Genome sequence of rock bream, Oplegnathus fasciatus (Temminck & Schlegel, 1884): the first draft genome in family Oplegnathidae<br>Yongshuang Xiao; Zhizhong Xiao; Jing Liu; Daoyuan Ma; Jun Li<br>GigaScience<br><br>Dear Dr. Xiao,<br><br>Your manuscript "Genome sequence of rock bream, Oplegnathus fasciatus (Temminck & Schlegel, 1884): the first draft genome in family Oplegnathidae" (GIGA-D-18-00300) has been assessed by our reviewers. Although it is of interest, we are unable to consider it for publication in its current form. The reviewers have raised a number of points which we believe would improve the manuscript and may allow a revised version to be published in GigaScience. In particular it needs significant editing by a native English speaker as the language needs a lot of work. Please also include details on common names of the species and NCBI taxon/Fishbase IDs, and other identifiers in the paper.<br>Reply:<br>We would like to give sincere thanks to the editor’s suggestions. In order to check the accurate species information, we have checked the taxonomy information from the WORMS (World Register of Marine Species)<br>http://www.marinespecies.org/aphia.php?p=search, Wikipedia<br>https://www.wikipedia.org/, NCBI<br>https://www.ncbi.nlm.nih.gov/Taxonomy/Browser/wwwtax.cgi?id=163133 and Fishes of the World (Fifth Editon) (Joseph S. Nelson, Terry C. Grande and Mark V. Wilson), and all of them supported the taxonomy of the Oplegnathidae. The Oplegnathidae occupied one genus composed of seven species Oplegnathus conwayi (Richardson, 1840), Oplegnathus fasciatus (Temminck & Schlegel, 1844), Oplegnathus insignis (Kner, 1867), Oplegnathus peaolopesi (Smith, 1947), Oplegnathus punctatus (Temminck & Schlegel, 1844), Oplegnathus robinsoni (Regan, 1916), Oplegnathus woodwardi (Waite, 1900).<br><br>Their reports, together with any other comments, are below. Please also take a moment to check our website at https://giga.editorialmanager.com/ for any additional comments that were saved as attachments.<br><br>If you are able to fully address these points, we would encourage you to submit a revised manuscript to GigaScience. Once you have made the necessary corrections, please submit online at:<br><br>https://giga.editorialmanager.com/<br><br>If you have forgotten your username or password please use the "Send Login Details" link to get your login information. For security reasons, your password will be reset.<br><br>Please include a point-by-point within the 'Response to Reviewers' box in the submission system. Please ensure you describe additional experiments that were carried out and include a detailed rebuttal of any criticisms or requested revisions that you disagreed with. Please also ensure that your revised manuscript conforms to the journal style, which can be found in the Instructions for Authors on the journal homepage.

Powered by Editorial Manager® and ProduXion Manager® from Aries Systems Corporation

The due date for submitting the revised version of your article is 08 Jan 2019.

I look forward to receiving your revised manuscript soon.

Best wishes,

Hongling Zhou
GigaScience
www.gigasciencejournal.com

Reviewer reports:

Reviewer #1: This manuscript describes the genome assembly and annotation of O. fasciatus, with little else by way of analysis. The methods used are mostly appropriate, and the assembly appears to be of high quality.

Some issues and suggestions:

1. The assembly contiguity is repeatedly referred to as 'remarkable', this is perhaps an exaggeration. These values are not extraordinary in the age of long-read sequencing. S Table 4 lists other fish assemblies, but includes almost no current-generation ones, flattering the assembly statistics obtained in this study.
Reply:
We would like to give sincere thanks to the reviewer's suggestions. We have thoroughly revised the manuscript for the description of the quality of the genome assembly. And we have deleted the degree word of "remarkable" as follows:
1) We revised the "which reached a remarkable high level of continuity with contig N50 of 2.1 Mb" as "which reached a high level of continuity with a contig N50 of 2.1 Mb" .
2) We revised the "which reached a remarkable high level of continuity with contig N50 length of 2.1 Mb" as "which reached a high level of continuity and a contig N50 of 2.1 Mb" .
3) We revised the "which showed a remarkable high level of continuity with contig" as "which showed a high level of continuity with a contig".
4) We revised the "which reached a remarkable high level of continuity with contig" as "which reached a high level of continuity with a contig".
5) We revised the "The contig N50 was remarkable longer than those of most fish" as "Contig N50 was longer than those of most fish".

Line 336-338: Meanwhile, we have highlighted that the important role of long reads in the contig continuity of genome assembly in the test as follows: "Previous studies illuminated the relationship between read length and genome assembly; therefore, we attributed the continuity of the genome primarily to the application of long reads in the assembly".
Table 4: According to the reviewer's comments, we also added the current-generation of other fish assemblies in the Table 4, which included Lepisosteus oculatus (Genome Size: 945 Mb, Contig N50: 0.07Mb, Scaffold N50: 6.9Mb), Sillago sinica (Genome Size: 534 Mb, Contig N50: 2.6Mb), Lates calcarifer (Genome Size: 586 Mb, Contig N50: 1.07Mb, Scaffold N50: 25.85Mb), Oreochromis niloticus (Genome Size: 868 Mb, Contig N50: 3.3Mb, Scaffold N50: 37Mb).

2. I will admit I am not an expert on Oplegnathidae. However, according to Wikipedia, the genus Oplegnathus contains seven species, and the common name for O. fasciatus is 'striped beakfish' or 'barred knifejaw'. The manuscript claims two species (line 68), and the common name 'rock bream'.
Reply:
We agreed with the reviewer's comment on the taxonomy of the Oplegnathidae. The Oplegnathidae occupied one genus composed of seven species Oplegnathus conwayi (Richardson, 1840), Oplegnathus fasciatus (Temminck & Schlegel, 1844), Oplegnathus insignis (Kner, 1867), Oplegnathus peaolopesi (Smith, 1947), Oplegnathus punctatus (Temminck & Schlegel, 1844), Oplegnathus robinsoni (Regan, 1916), Oplegnathus woodwardi (Waite, 1900). We have checked the taxonomy information from the WORMS (World Register of Marine Species) http://www.marinespecies.org/aphia.php?p=search, Wikipedia

Powered by Editorial Manager® and ProduXion Manager® from Aries Systems Corporation

https://www.wikipedia.org/, NCBI
https://www.ncbi.nlm.nih.gov/Taxonomy/Browser/wwwtax.cgi?id=163133 and Fishes of the World (Fifth Editon) (Joseph S. Nelson, Terry C. Grande and Mark V. Wilson), and all of them supported the taxonomy of the Oplegnathidae.
It's our mistake in the text for the verification of species numbers. We know two (Oplegnathus fasciatus and O. punctatus) of seven species existed in the coastal waters of East Asia.
We also checked the common name of O. fasciatus, the common name of rock bream is incorrect and we revised it as "barred knifejaw" based on the reviewer's comments, NCBI and Wikipedia. We also revised it in the text.
We used the common name "barred knifejaw" instead of "rock bream" in the text.

3. Line 109: 'a repeat content of 38.46%', how was this calculated? It does not follow from figure 2.
Reply: The K-mer distribution from the sequencing data could be used for the genome size, heterozygosity and repeat content ratio estimation, mainly from the relative numbers of homozygous, heterozygous and repeated Kmers, using the statistical model described in the previous study (Liu, B. et al. Estimation of genomic characteristics by analyzing k-mer frequency in de novo genome projects. Quantitative Biology 35, 62-67 (2013)). We have illuminated the peaks raised by homozygous, heterozygous and repeated K-mers in Figure 2.

4. Line 107/111: The k-mer estimate and the intial assembly yield exactly the same genome size (808.9 Mbp). This is highly unlikely, especially if the genome is highly repetitive, as claimed here.
Reply:
We would like to give sincere thanks to reviewer's suggestions. We have carefully checked our sequencing results and found there was a clerical error in the text.
Line 286: According to the estimation of K-mer, the genome size is 786.46Mb, and after eliminating the influence of K-mer error, we get the genome size is 777.5Mb.
Line 289: According to the assembly of platanus, the contig N50 is 7.19kb with total length of 875.4Mb. And then reached to the level of scaffold N50 is 84.126kb with total length of 744.53Mb.
So, it is a clerical error in the text, and we have revised them in the text.

5. Line 123: I assume the contigs and scaffold listed here, to which the HiC data map, are those of the final (PacBio-based) assembly. However, the only assembly that has been described at this point is the highly fragmented initial one. Perhaps you could restructure this so that the HiC sequencing is described after PacBio sequencing.
Reply:
The reviewer is correct. The genome used here for the Hi-C data evaluation is the genome assembled from the PacBio sequencing data. We moved this part after the PacBio sequencing data assembly.

6. Figure 2 shows a clear bump corresponding to duplicated k-mers (at 200). Is this duplication level still relevant for the final assembly? For example, a lot of sequence is removed (line 136) based on redundancy, and a large fraction of PacBio reads do no map to the final assembly (line 158). Is there a relation with the sex chromosome configuration (X1X1X2X2, line 76)?
Reply: We agree with the reviewer's comment on the repeat content of the final assembly. We noticed that the repeat content of final genome were about 33.9%, which was lower than that from the genome survey estimation (38.5%). The high repetitive elements in repeated regions of chromosomes, such as those in the sex chromosome, might result into fragmented assembly. Those repeated sequences might be removed in the redundancy elimination process. We have added the discussion into our revised manuscript as follows:
Line 437-440: "Note that the mapping ratio might be related to the repetitive content of the O. fasciatus genome, especially for the high repeat content in the sex chromosomes6. However, how the repetitive elements in the genome influence the karyotypes of this species needs further investigation."

7. Line 140: That the polishing is performed using Pilon should be mentioned here (it is

mentioned in S table 2). Also, 'using NGS data' is ambiguous, as PacBio also qualifies as NGS. This probably refers to Illumina only.
Reply: Thanks a lot for the reviewer's suggestion. We have added the description of Pilon for the sequence polishing in the manuscript. "Using NGS data" referred to Illumina data only here, we therefore clarify the sentence in the manuscript as follows: "The resulting genome assembly was further polished using Illumnina NGS data"

8. Figure 4 and S Figure 4 analyze O. fasciatus in the context of 'fish species'. While this is technically correct, it is biologically not always the most relevant comparison. Fish species such as ghost shark and lancelet are included, but for example tetrapods (which are more closely related to O. fasciatus than the aforementioned fish) are not. In figure 4, these make for less appropriate outgroups (because of their very distant relationship to the other, teleost, fish species). I would suggest including at least e.g. spotted gar to the analysis to fill this gap (and perhaps omit B. floridae).
Reply: We agree with the reviewer. We have added the spotted gar and deleted B. floridae in our phylogenetic analysis, and re-preformed the gene family construction and phylogenetic analysis. The result of phylogeny including the spotted gar was consistent with reviewer's prospection, which filled the gap from the fish evolution process in our study. We would like to give sincere thanks to reviewer's suggestions. The revised phylogenetic results were illuminated in the Figure 4.

8. Figure 4 needs more information in the legend. What do the numbers mean exactly, and how were they calculated? The conclusion drawn from this figure (line 266) is not appropriate, as the phylogenetic position of Notothenioidei is not relevant to the narrative of this manuscript, and reclassification needs more evidence than this sparse phylogenetic tree.
Reply: Thanks a lot for the reviewer's suggestion. We have added more information in the legends for the Figure 4. The descriptions of the phylogenetic analysis were revised in the manuscript. And we agree with the reviewer's suggestion that the phylogenetic position of Notothenioidei is not relevant to the narrative of our manuscript and we deleted it.

9. One of the motivations for sequencing this genome is understanding the fish' sex determining system. This aim is not revisited in the results or Conclusion. How does the choice of a female individual for genome sequencing affect this goal?
Reply:
Thanks a lot for the reviewer's suggestion. We have added the sentence for the importance of genome in our following genetic studies to understand the sex-determining system of the fish species. The reason we chose a female one for the genome assembly because the female ones do not have heterotropic chromosome, which might facilitate the chromosome assembly of X1 and X2. The quality of X1 and X2 could lay a solid foundation for the chromosome analysis in our following studies. We have added the discussion in the conclusion as follows: "As far as we known, the Y chromosomes has always exhibited many specific sequence characteristics compared to X1 and X2, such as repeat content, and those differences might increase the difficulty of the sequence assembly of chromosomes X1 and X2. The chromosome-level genome assembly together with gene annotation data generated for the female fish in this work will provide a valuable resource for further research on sex-determining mechanisms, especially for obtaining an accurate assembly of the Y chromosome in male fish. These results will also accelerate genome-wide association studies in resistant breeding systems."

Typos:
L 102 Hieq -> HiSeq
L 172 RepreatMasker -> RepeatMasker
Reply: Thanks for the reminding from the reviewer. We have revised it in the text.

Reviewer #2: In this manuscript Xiao et al. reports the genome assembly of the rock bream (O. fasciatus) a species of increasing economic importance in Asia. This species exhibits sex dimorphism in growth and also a sex determination system based on multiple sex chromosomes X1X1X2X2/X1X2Y, which makes it interesting species to study. The draft genome of the rock bream will be a valuable resource to facilitate

	future research aimed at improving relevant traits and understanding of determination systems.<br>The authors used an adequate amount of sequence data from three different sources (Illumina short reads, PacBio and Hi-C), which allowed them to generate a robust genome assembly. Furthermore, the authors annotated the genome using multiple strategies. Finally, they carried out some phylogenetic analyses including other fish species. The methods followed to obtain the assembly are good in general, and well described.<br><br>L33-L35 Please re-phrase, maybe say "sexual dimorphism in growth"<br>Reply: Thanks a lot for the reviewer's suggestion<br>Line 37-38 According to the reviewer's comments, we revised the "growth of sexual dimorphism with neo-sex chromosome and widespread biotic" as "sexual dimorphism in growth with neo-sex chromosome and widespread biotic".<br>L37 ...basing -> based<br>Reply: Thanks a lot for the reviewer's suggestion, we have revised the manuscript in the text. We revised the "basing" as "based".<br><br>L43 "…We assembled the O.fasciatus" <genome?><br>Reply: Thanks a lot for the reviewer's suggestion, we have revised the manuscript in the text. We added the "genome" after the "O.fasciatus"<br><br>L77 Again please re-phrase "the growth sexual dimorphism"<br>Reply: Thanks a lot for the reviewer's suggestion, we have revised the manuscript in the text. We revised the "the growth sexual dimorphism" as "sexual dimorphism in growth".<br>L99-L100 "A whole genome using Illumina DNA seq…" re-phrase<br>Reply: According to the reviewer's comments, we have revised the manuscript i in the text. We revised the "A whole-genome using Illumina DNA sequencing technology was applied to estimate O. fasciatus genome size." as "The whole-genome size of O. fasciatus was estimated based on the Illumina DNA sequencing technology".<br><br>L115 Was the blood extracted from the same fish used for pacBio and Illumina?<br>Reply: Thanks a lot for the reviewer's question. In order to avoid the genetic-background influence of individual difference, expecially for the HI-C result, the blood was extracted from the same female fish of O. fasciatus used for pacBio and Illumina.<br><br>L162 "the results showed <that> 99.8%.."<br>Reply: Thanks a lot for the reviewer's suggestion, we have revised the manuscript in the text. We added the "that" after the "showed" as "the result showed that 99.8%......"<br><br>L172 Typo Repreatmasker<br>Reply: We have revised the manuscript in the text. We revised the "Repreatmasker" as "RepeatMasker".<br><br>L252 Is not clear how you came up to those 812 orhtogroups, and the same for L256<br>Reply: 21,528 gene families were constructed from the gene family clustering. However, most of the gene families contained more than one gene for species in our studies. To eliminate uncertain effects for the phylogenetic analysis from duplicated genes, we only selected gene families that contain one and only one genes for each species. In our case we obtained 1236 gene families (1236 genes) for the phylogenetic analysis. After removing short gene (length shorter than 100 amino acid (about 300bp)), we obtained 1158 genes for the final anlaysis.<br><br>L266 I don't think the authors should claim that the Notothenioidei should be elevated to the order level, but I would accept that their results suggest or show evidence of this.<br>Reply: Thanks a lot for the reviewer's suggestion. We have revised our conclusion from the phylogenetic analysis. Indeed, we cannot claim the phylogenetic position of Notothenioidei from our data, but our result could provide useful knowledge for the related studies. We think that the phylogenetic position of Notothenioidei is not relevant to the narrative of our manuscript and we deleted it.<br><br>General Comments:<br>There are many issues with the English throughout the manuscript and these must be

Powered by Editorial Manager® and ProduXion Manager® from Aries Systems Corporation

	addressed before considering for publication. I strongly encourage the authors to proof-read the manuscript before re-submitting.<br>Reply: Thanks for the editor's suggestion. We have revised the English throughout the manuscript with the service of AJE (American Journal Experts). We hoped that the English now could meet the standard for the GigaScience. The revision places as follows:<br>Line 1 we revised "Genome sequence of barred knifejaw,…" as "Genome sequence of the barred knifejaw,…".<br>Line 3 we revised "the first draft genome in family Oplegnathidae" as "the first chromosome-level draft genome in the family Oplegnathidae".<br>Line 33 we revised "The barred knifejaw (Oplegnathus fasciatus),…" as "The barred knifejaw Oplegnathus fasciatus,…".<br>Line 34 we revised "commerically" as "commercially".<br>Line 38 we revised "has received" as "has been received".<br>Line 39-40 we revised the sentence "However, the adequate genome resources to make insight into sex-determining mechanism and to establish genetically based resistant breeding systems for O. fasciatus have been lacking. " as "However, adequate genome resources for gaining insight into sex-determining mechanisms and establishing genetically based resistant breeding systems for O. fasciatus are lacking.".<br>Line 41-43 we revised the sentence "we performed whole genome of female fish for O. fasciatus using long-read sequencing and Hi-C data to generate chromosome-length scaffolds with highly contiguous genome assembly." as "we analysed the entire genome of a female O. fasciatus fish using long-read sequencing and Hi-C data to generate chromosome-length scaffolds and a highly contiguous genome assembly."<br>Line 45 we revised ", which" as "that".<br>Line 46 we revised "both of" as "both the". And we also revised the "Hiseq" as "HiSeq".<br>Line 48 we added "a" in front of "contig N50".<br>Line 49-53 we revised the sentence as "We combined Hi-C data with a draft genome assembly to generate chromosome-length scaffolds. Twenty-four scaffolds corresponding to the twenty-four chromosomes were assembled to a final size of 768.8 Mb with a contig N50 of 2.1 Mb and a scaffold N50 of 33.5 Mb using 1,372 contigs." .<br>Line 53 we revised "account" as "accounted".<br>Line 55 we revised "annotated using de novo method and" as "annotated using de novo methods, ".<br>Line 55 we revised "homologies" as "homology". We also revised "with draft" as "with a draft".<br>Line 56 we deleted both of "the" and "the".<br>Line 57-58 we revised the sentence "was close related to Larimichthys crocea and O. fasciatus diverged from their ancestor was at about 70.3-87.3 million years ago." as "is closely related to Larimichthys crocea, with O. fasciatus diverging from their common ancestor approximately 70.3-87.3 million years ago".<br>Line 60 we revised the sentence "We generated high-quality draft genome and chromosomes assembly" as "We generated a high-quality draft genome with chromosome assembly".<br>Line 146we revised "is" as "represents".<br>Line 147-149 we revised the sentence "The genome assembly will provide insight into sex-determining mechanism and serve as a resource for accelerating the genome-assisted improvement of resistant breeding systems." as "Assembly of this genome will provide insight into sex-determining mechanisms and serve as a resource for accelerating genome-assisted improvements in resistant breeding systems.".<br>Line 154 we revised "The family Oplegnathidae belongs" as "The Oplegnathidae family".<br>Line 155 we revised "including only one genus Oplegnathus comprised of" as "including only one genus Oplegnathus, which is comprised of".<br>Line 156 we revised "two (O. fasciatus and O. punctatus) of which" as "two of which (O. fasciatus and O. punctatus)".<br>Line 157 we revised "commercial values in East Aisa" as "commercially valuable in East Asia".<br>Line 158 we deleted "," in both sides of "O. fasciatus (Temminck & Schlegel, 1844)".<br>Line 158 we revised "the two" as "these two".<br>Line 159 we revised "meters" as "metres".<br>Line 160 we revised "being distributed in" as "and distributed across".<br>Line 163-164 we revised "It was reported that the male of Oplegnathus has a neo-sex chromosome" as "It has been reported that the male Oplegnathus possesses a neo-

	sex chromosome".<br>Line 164 we revised ", and the" as ". The".<br>Line 165 we revised "was" as "is".<br>Line 166 we deleted "the" in front of "karyotype analyses".<br>Line 166 we revised the "was" as "has been".<br>Line 167 we revised "and the male fish showed a faster growth advantage than the female" as ", with male fish exhibiting faster growth than females". We also revised "may" as " possibly".<br>Line 168 we revised "of" as "in".<br>Line 171 we revised "for making" as "to gain". We also revised "accelerating" as "to accelerate".<br>Line 172 we revised "improvement of" as "improvements in".<br>Line 173 we revised "So far, the genome sequence with the chromosomes assembly" as "So far, a genome sequence with the chromosomal assembly".<br>Line 263 we revised "Here we performed" as "Here, we constructed".<br>Line 264 we deleted "constructed".<br>Line 265 we revised "using" as "used".<br>Line 266 we revised "assemblyer Canu" as "assembly program Canu". We also revised "the" as "this".<br>Line 267 we revised "the family Oplegnathidae" as "the Oplegnathidae family".<br>Line 270 we revised "improvement of" as "improvements in".<br>Line273 we added "sequencing using" in front of "the Illumina platform".<br>Line 276 we revised "sample of" as "samples from".<br>Line 277 we deleted "the".<br>Line 280 we added "the" in front of "Illumina HiSeq X Ten platform".<br>Line 281 we added "the" before "removal of low-quality and redundant reads".<br>Line 282 we revised "about" as "approximately".<br>Line 283 we deleted "the" in front of "cleaned reads".<br>Line 284 we revised "about" as "approximately".<br>Line 285 we added "was" in front of "at a depth of 100".<br>Line 287 we revised "the" as "an".<br>Line 290 we revised "contig N50 7.2 kb and scaffold N50 84.1kb" as "contig N50 of 7.2 kb and a scaffold N50 of 84.1kb".<br>Line 292 we added "," in front of "partly due to". We also revised "genomics" as "genomic".<br>Line 317 we added "the" in front of "PacBio".<br>Line 318 we revised "obtain" as "obtained".<br>Line 319 we revised "totally 62.8 Gb" as "62.8 Gb in total". We also revised "a read N50" as "an N50 read".<br>Line 321 we revised "The Canu" as "Canu".<br>Line 322-323 we revised "As a result, a total length of 875.9 Mb genome assembly was achieved for O. fasciatus" as "As a result, a genome assembly with a total length of 875.9 Mb was constructed for O. fasciatus".<br>Line 323 we deleted "which was".<br>Line 324 we revised "the estimated genome size in 17-mer analysis" as "the genome size estimated by 17-mer analysis".<br>Line 325 we revised "relative" as "relatively".<br>Line 325-326 we revised "the complexity of genome such as heterozygosity" as "the complexity of this genome to factors such as heterozygosity".<br>Line 327 we revised "and obtain genome" as "to obtain a".<br>Line 328 we revised "the Arrow of Smrtlink 5.0" as "the Arrow tool in SMRT Link 5.0 software".<br>Line 329 we deleted "the" in front of "the error correction".<br>Line 335 we revised "technologies, and is comparable with" as "technologies and is comparable to".<br>Line 341-342 we revised "depended strongly on" as "are strongly dependent upon".<br>Line 395-396 we revised the sentence "The genomic DNA for Hi-C library was extracted from the whole-blood cell of O. fasciatus as described" as "Genomic DNA was extracted for the Hi-C library from a whole-blood sample of O. fasciatus as described".<br>Line 397 we revised "digested" as "was digested".<br>Line 397 we revised "biotin-labeled" as "biotin-labelled".<br>Line 401 we added "were produced" in front of "with Q20 and".<br>Line 402 we added "the" in front of "Hi-C data".

Powered by Editorial Manager® and ProduXion Manager® from Aries Systems Corporation

Line 407 we revised “other” as “more”.
Line 409 we revised “those” as “these sequences”.
Line 411 we revised “interactions map” as “the interaction map”.
Line 413 we revised “contigs” as “polished contigs”.
Line 414 we added “were assembled” in front of “corresponding to”.
Line 419 we revised “reached” as “attained”.
Line 427 we added “assembled” in front of “sequences”.
Line 431 we deleted “both of”.
Line 433 we deleted “the” in front of “Minimap2”.
Line 434 we deleted “the” in front of “CLR”.
Line 435 we revised “checked for” as “assessed in the”.
Line 436-437 we revised “sequencing depth were reached to 90.2%, 99.9% and 80.6” as “sequencing depth reached 90.2%, 99.9% and 80.6”.
Line 441-442 we added “the” in front of “O. fasciatus” and “whole-genome” respectively.
Line 443 we deleted “the” in the front of “GATK”. We also deleted “the” in front of “SNP”.
Line 444 we revised “the result” as “and the results”.
Line 445 we revised “heterozygosis and homology” as “heterozygous and homologous”.
Line 446 we revised “yield” as “yielded”.
Line 447 we revised “the estimate from k-mer” as “the k-mer estimate analysis”.
Line 449 we revised “Repeat sequence” as “Repeat sequences”.
Line 502 we deleted “the” in front of “TE-related proteins”.
Line 504 we revised “account” as “accounted”.
Line 505 we revised “included” as “including”.
Line 509 we revised “The long interspersed nuclear elements (LINE) and long terminal repeat (LTR)” as “Long interspersed nuclear elements (LINEs) and long terminal repeats (LTRs)”.
Line 510 we revised “took up 7.3% and 4.0% of the whole genome” as “comprised 7.3% and 4.0% of the whole genome, respectively”.
Line 512 we added “that were” in front of “used for”.
Line 513-514 we revised “High quality of RNA were detected” as “RNA quality was determined”.
Line 514 we added “ratio of ” in front of “absorbance”.
Line 515 we added “using a” in front of “Nanodrop ND-1000”.
Line 516 we added “a” in front of “2100 Bioanalyzer”.
Line 517 we deleted “the process of” in front of “reverse transcription”.
Line 518 we revised “The” as “A”. We also deleted “the manual of” in front of “the Paired-End Sample”.
Line 519 we revised “the library” as “a library”.
Line 525 we revised “prediction” as “predictions”.
Line 527 we revised “of” as “in the”.
Line 530 we revised “to” as “the”.
Line 583 we deleted “the”.
Line 586 we revised “then we” as “we then”. We also deleted “the” in front of “gene”.
Line 589 we added “the” in front of “O. fasciatus genome”.
Line 591 we revised “in” as “of”.
Line 593-596 we revised the sentence “In order to further obtain functional annotation of the protein-coding genes in O. fasciatus genome, we employed local BLASTX and BLASTN programs to align upon the non-redundant protein (NR), non-redundant nucleotide (NT) and Swissprot database with an e-value ≤ 1e-5” as “To obtain further functional annotation of the protein-coding genes in the O. fasciatus genome, we employed the local BLASTX and BLASTN programs and the Swiss-Prot database with an e-value ≤ 1e-5 to align the non-redundant nucleotides (NT) and the non-redundant proteins (NR), respectively”.
Line 597 we revised “Kyoto Encyclopedia of Genes” as “and Kyoto Encyclopaedia of Genes”.
Line 598 we revised “Finally” as “Ultimately”.
Line 601 we added “the” in front of “tRNAscan-SE”.
Line 609 we revised “gene family” as “gene families”.
Line 610 we revised “of” as “the”.
Line 611 we revised “in” as “using”.
Line 774 we revised “relationship” as “relationships”.

Powered by Editorial Manager® and ProduXion Manager® from Aries Systems Corporation

	Line 775 we revised “single-copy gene” as “single-copy genes”.<br>Line 776 we revised “length filter” as “a length filter”. We also deleted “, respectively”.<br>Line 778 we revised “sequence of each species” as “sequences for each species”.<br>Line 782 we deleted “a” in front of “molecular clock”.<br>Line 784-785 we revised “were clustered together with Larimichthys crocea belonged to” as “clustered with Larimichthys crocea in”.<br>Line 787 we revised “about” as “approximately”.<br>Line 788 we revised “Conclusion” as “Conclusions”.<br>Line 791-793 we revised the sentence as “The final draft genome assembly is approximately 778.7 Mb, which was slightly higher than the estimated genome size (777.5 Mb) based on k-mer analysis”.<br>Line 793-795 we revised the sentence as “Those contigs were scaffolded to chromosomes using Hi-C data, resulting a genome with a high level of continuity with a contig N50 of 2.1 Mb and a scaffold N50 of 33.5 Mb.”.<br>Line 799-800 We revised the sentence “We found the divergence time between O. fasciatus and the common ancestor with Larimichthys crocea was at about 70.3-87.3 Ma” as “We found that the divergence time between O. fasciatus and its the common ancestor with Larimichthys crocea was approximately 70.3-87.3 Ma”.<br><br>I wonder why the authors chose to sequence a female fish, while the male fish could have had provided the full sequence of the Y chromosome which could've brought insights into sex determination, the identification of sex specific regions, etc. I mention this because you stress that the genome assembly is useful for the understanding of these mechanisms this but then there's no mention of this important topic in the discussion.<br>Reply:<br>Thanks for the editor’s concerns. We indeed have a plan for the genome assembly for a male one, after this female genome work. The reason we choose a female one because of the heterotropic chromosome in males. As far as we known, Y chromosomes exhibited lots of specific sequence characters, such as repeat content, comparing to X1 and X2, and those differences might increase the difficulty for the sequence assembly of chromosome X1 and X2. Based on this genome, the male genome assembly will be carried out in the following work, with the aim to get the accurate assembly of Y chromosome.<br>We have added the discussion in the conclusion in line 364-386 as follows: “As far as we known, the Y chromosomes has always exhibited many specific sequence characteristics compared to X1 and X2, such as repeat content, and those differences might increase the difficulty of the sequence assembly of chromosomes X1 and X2. The chromosome-level genome assembly together with gene annotation data generated for the female fish in this work will provide a valuable resource for further research on sex-determining mechanisms, especially for obtaining an accurate assembly of the Y chromosome in male fish. These results will also accelerate genome-wide association studies in resistant breeding systems.”<br><br>Reviewer3:<br>Further to my previous email, another referee noted that Oplegnathidae is no longer a part of the Perciformes, according to the Betancur-R et al. 2017 phylogenetic classification of fishes, who placed it in the Centrarchiformes. Please also include this detail in the introduction.<br>Reply:<br>Thanks for the editor’s suggestions. We have carefully checked the two papers (Betancur-R. R, Broughton RE, Wiley EO, Carpenter K, López JA, Li C, Holcroft NI, Arcila D, Sanciangco M, Cureton II JC, Zhang F, Buser T, Campbell MA, Ballesteros JA, Roa-Varon A, Willis S, Borden WC, Rowley T, Reneau PC, Hough DJ, Lu G, Grande T, Arratia G, Ortí G. The Tree of Life and a New Classification of Bony Fishes. PLOS Currents Tree of Life. 2013 and Ricardo Betancur-R, Edward O. Wiley, Gloria Arratia, Arturo Acero, Nicolas Bailly, Masaki Miya,Guillaume Lecointre and Guillermo Ortí. Phylogenetic classification of bony fishes. 2017) and the book (Fishes of the World (Fifth Editon) (Joseph S. Nelson, Terry C. Grande and Mark V. Wilson)).<br>We agreed with the reviewer’s suggestion and we also agreed with the molecular taxonomy results. We have revised the information in the abstract, introduction and Gene family identification and phylogenetic tree construction sections of the text. We have referenced the paper in the discussion section,
**Additional Information:**	

Powered by Editorial Manager® and ProduXion Manager® from Aries Systems Corporation

Question	Response
Are you submitting this manuscript to a special series or article collection?	No
**Experimental design and statistics**<br><br>Full details of the experimental design and statistical methods used should be given in the Methods section, as detailed in our Minimum Standards Reporting Checklist. Information essential to interpreting the data presented should be made available in the figure legends.<br><br>Have you included all the information requested in your manuscript?	Yes
**Resources**<br><br>A description of all resources used, including antibodies, cell lines, animals and software tools, with enough information to allow them to be uniquely identified, should be included in the Methods section. Authors are strongly encouraged to cite Research Resource Identifiers (RRIDs) for antibodies, model organisms and tools, where possible.<br><br>Have you included the information requested as detailed in our Minimum Standards Reporting Checklist?	Yes
**Availability of data and materials**<br><br>All datasets and code on which the conclusions of the paper rely must be either included in your submission or deposited in publicly available repositories (where available and ethically appropriate), referencing such data using a unique identifier in the references and in the "Availability of Data and Materials" section of your manuscript.	Yes

Powered by Editorial Manager® and ProduXion Manager® from Aries Systems Corporation

Have you have met the above requirement as detailed in our Minimum Standards Reporting Checklist?	

Manuscript

Click here to view linked References

Click here to access/download;Manuscript;New-Manuscript.docx

Genome sequence of the barred knifejaw *Oplegnathus fasciatus* (Temminck & Schlegel, 1884): the first chromosome-level draft genome in the family Oplegnathidae

Yongshuang Xiao[1,2,3*] †, Zhizhong Xiao[1,2,3,] †, Daoyuan Ma[1,2,3], Jing Liu[2,3*], Jun Li[1,2,3*]

[1]CAS Key Laboratory of Experimental Marine Biology, Institute of Oceanology, Chinese Academy of Sciences, 7 Nanhai Road, Qingdao, 266071, China, [2] Laboratory for Marine Biology and Biotechnology, Qingdao National Laboratory for Marine Science and Technology, 7 Nanhai Road, Qingdao, 266071, China, [3]Center for Ocean Mega-Science, Chinese Academy of Sciences, 7 Nanhai Road, Qingdao, 266071, China

*Correspondence address: Yongshuang Xiao, Mega-Science, Chinese Academy of Sciences, 7 Nanhai Road, Qingdao, 266071, China; Tel: +86-053282896729; E-mail: dahaishuang1982@163.com; Jing Liu, Institute of Oceanology, Chinese Academy of Sciences, 7 Nanhai Road, Qingdao, 266071, China; Tel: +86-053282898790; E-mail: jliu@qdio.ac.cn; Jun Li, Institute of Oceanology, Chinese Academy of Sciences, 7 Nanhai Road, Qingdao, 266071, China; Tel: +86-053282898718; E-mail: junli@qdio.ac.cn.

†Contributed equally to this work.

Abstract

Background

The barred knifejaw (*Oplegnathus fasciatus*), a member of the Oplegnathidae family of the Centrarchiformes, is a commercially important rocky reef fish native to East Asia. *O. fasciatus* has become an important fishery resource for offshore cage aquaculture and fish stocking of marine ranching in China, Japan and Korea. Recently, sexual dimorphism in growth with neo-sex chromosome and widespread biotic diseases in *O. fasciatus* has been received increasing concern. However, adequate genome resources for gaining insight into sex-determining mechanisms and establishing genetically resistant breeding systems for *O. fasciatus* are lacking. Here, we analysed the entire genome of a female *O. fasciatus* fish using long-read sequencing and Hi-C data to generate chromosome-length scaffolds and a highly contiguous genome assembly.

Findings

We assembled the *O. fasciatus* genome with a total of 245.0 Gb of raw reads that were generated using both of PacBio Sequel and Illumina HiSeq 2000 platforms. The final draft genome assembly was approximately 778.7 Mb, which reached a high level of continuity with a contig N50 of 2.1 Mb. The genome size was consistent with the estimated genome size (777.5 Mb) based on *k*-mer analysis. We combined Hi-C data with a draft genome assembly to generate chromosome-length scaffolds. Twenty-four scaffolds corresponding to the twenty-four chromosomes were assembled to a final size of 768.8 Mb with a contig N50 of 2.1 Mb and a scaffold N50 of 33.5 Mb using 1,372 contigs. The identified repeat sequences accounted for 33.9% of the entire genome, and 24,003 protein-coding genes with an average of 10.1 exons per gene were annotated using *de novo* methods, with RNA-seq data and homologies to other teleosts. According to phylogenetic analysis using protein-coding genes, *O. fasciatus* is closely related to *Larimichthys crocea*, with *O. fasciatus* diverging from their common ancestor approximately 70.5-88.5 million years ago.

Conclusions

We generated a high-quality draft genome with chromosome assembly for *O. fasciatus* using long reads by using the PacBio sequencing technologies, which represents the first chromosome-level reference genome for Oplegnathidae species. Assembly of this genome will provide insight into sex-determining mechanisms and serve as a resource for accelerating genome-assisted improvements in resistant breeding systems.

Keywords: *Oplegnathus fasciatus*; chromosome-level genome assembly; Hi-C assembly; sex-determining mechanism

Data description

Introduction of *O. fasciatus*

The Oplegnathidae family belongs to the order Centrarchiformes, including only one genus *Oplegnathus,* which is comprised of seven species (*O. conwayi*, *O. fasciatus*, *O. insignis*, *O. peaolopesi*, *O. punctatus*, *O. robinsoni*, *O. woodwardi*), two of which (*O. fasciatus* and *O. punctatus*) are commercially valuable in East Asia. The barred knifejaw *O. fasciatus* (Temminck & Schlegel, 1844) is one of these two species in the *Oplegnathus,* which is commonly found at the depth of one to ten metres in association with rocky reefs[1, 2], and distributed across a wide range of shallow waters around Korea, Japan, China and Hawaii[1, 3, 4] (Fig. 1). *O. fasciatus* has become an important fishery resource for offshore cage aquaculture and fish stocking of marine ranching in China, Japan and Korea[5]. It has been reported that the male of *Oplegnathus* possesses a neo-sex chromosome, possibly a sex chromosome Y. The sex chromosome system for *Oplegnathus* is considered to be $X_1 X_1 X_2 X_2$ / $X_1 X_2 Y$ based on karyotype analyses[6, 7]. Furthermore, sexual dimorphism in growth has been detected in the *O. fasciatus*, with male fish exhibiting faster growth than females, possibly be due to the sex chromosome system in *Oplegnathus*[8]. *O. fasciatus* is vulnerable to viruses (e.g., Iridovirus) and genetic degradation caused by inbreeding has led to higher susceptibility to diseases[9, 10]. It is vital to develop genomic resources to gain insight into sex-determining mechanisms and to accelerate the

genome-assisted improvements in resistant breeding systems.

So far, a genome sequence with the chromosomal assembly of *O. fasciatus* has not been reported. Here, we constructed a high-quality chromosome-level reference genome assembly for *O. fasciatus* using long reads by using the PacBio DNA sequencing platform and used a genome assembly strategy by taking advantage of genome assembly program Canu[11]. This genome assembly of *O. fasciatus* is the first chromosome-level reference genome constructed for the Oplegnathidae family. The completeness and continuity of the genome will provide high quality genomic resources for studies on sex-determining mechanisms and for accelerating the genome-assisted improvements in resistant breeding systems.

Genomic DNA extraction, genome size estimation

High-quality genomic DNA for sequencing using the Illumina platform (Illumina Inc., San Diego, CA, USA) and PacBio Sequel sequencing (Pacific Biosciences of California, Menlo Park, CA, USA) was extracted from fresh muscle tissue and blood samples from a single female *O. fasciatus*. The fish was collected from the near-shore area of Qingdao city (Yellow Sea), Shandong province. The whole-genome size of *O. fasciatus* was estimated based on Illumina DNA sequencing technology. A short-insert library (300~350 bp) was constructed and generated a total of ~90.7 Gb of raw reads using the standard protocol provided by the Illumina HiSeq X Ten platform (Illumina Inc., San Diego, CA, USA). After the removal of low-quality and redundant reads, we obtained approximately ~80.8 Gb of clean data for *de novo* assembly to estimate the whole-genome size (S Table 1, Fig. 2). All cleaned reads were subjected to 17-mer frequency distribution analysis[12]. As the total number of *k*-mers was approximately 8.09×10^{10} and the peak of *k*-mers was at a depth of 100, the genome size of *O. fasciatus* was calculated to be 777.5 Mb using the following formula with amendment: genome size = *k*-mer number / peak depth (Fig. 2). Meanwhile, an estimated heterozygosity of 0.29% and a repeat content of 38.46% were detected for *O. fasciatus* in this work. A pilot genome assembly was approximately 744.5 Mb with a contig N50 of 7.2 kb and a scaffold N50 of 84.1kb using the Illumina data and the

assembly program Platanus package[13] (S Table 2). The GC content was 41% (S Fig. 1). This genome assembly was of low-quality, partly due to its high genomic repeat content.

Genome assembly using PacBio long reads

Two 20 kb genomic DNA libraries were constructed and sequenced using the PacBio Sequel platform, generating 62.9 Gb raw DNA reads. We obtained 4.8 million subreads (62.8 Gb in total) with an N50 read length of ~22 kb after removing adaptor (S Table 1).

Canu v1.4 was firstly used to assemble the genome with the Corrected-Error-Rate parameter set at 0.040[11]. As a result, a genome assembly with a total length of 875.9 Mb was constructed for *O. fasciatus*, slightly higher than the genome size estimated by 17-mer analysis based on the Illumina data (S Table 2). We attributed the relatively large genome size of the assembly to the complexity of this genome to factors such as heterozygosity. We therefore applied Redundans v0.13c[14] to remove the sequence redundancy to obtain a genome assembly size of 778.0 Mb. We then used the Arrow tool in SMRT Link 5.0 software with the minCoverage parameter set at 15 to implement error correction based on the PacBio long reads data (Table 1). The resulting genome assembly was further polished using Illumina NGS data, which were used in the genome survey analysis above. The final draft genome assembly was 778.7 Mb, which reached a high level of continuity with a contig N50 length of 2.1 Mb (Table 1). The contig N50 of *O. fasciatus* was much higher than those of previous fish genome assemblies constructed using NGS DNA sequencing technologies and is comparable to those of recently reported model fish species (S table 3). Previous studies illuminated the relationship between read length and genome assembly; therefore, we attributed the continuity of the genome primarily to the application of long reads in the assembly.

Hi-C library construction and chromosome assembly

Hi-C is a sequencing-based approach for determining chromosome interactions by calculating the contact frequency between pairs of loci, which are strongly dependent

upon the one-dimensional distance, in base pairs, between a pair of loci[15, 16]. In this work, we used Hi-C to construct the genome assembly of *O. fasciatus*.

Genomic DNA was extracted for the Hi-C library from a whole-blood sample of *O. fasciatus* as described[17]. Cells were fixed with formaldehyde and lysed, and the cross-linked DNA was digested with MboI. Sticky ends were biotin-labelled and proximity ligated to form chimeric junctions and then physically sheared to a size of 300–500 bp[17]. Chimeric fragments representing the original cross-linked, long-distance physical interactions were then processed into paired-end sequencing libraries, and 629 million 150-bp paired-end Illumina reads (91.5 Gb) were produced with Q20 and Q30 of ~94.0% (S Table 1, S Table 4). By mapping the Hi-C data to the PacBio-based assembly using BWA software, we found that sequencing data with mates mapped to a different contig (or scaffold) and data mapped to a different contig (or scaffold) (map Q5⩾ 5) were 593.7 Mb (94.4%), 240.5 Mb (40.5%) and 205.1 Mb (34.6%), respectively (S Table 4). We then further employed BWA and Lachesis software to align paired-end reads to filter all base sequences than 500bp from each restriction site[18]. According to the conduct of clustering, ordering, and orienting to the assembly contigs (1,692), these sequences were grouped into 24 chromosome clusters and scaffolded using Lachesis software with tuned parameters[19] (Table 2, Fig. 3). Finally, we constructed the chromosome interactions map using Juicer software and employed the JucieBox to complete the visual correction of the interaction map. We obtained polished 1,756 polished contigs by interrupting misassembly from 1,692 contigs. Twenty-four scaffolds were assembled corresponding to the 24 chromosomes of *O. fasciatus* based on the karyotype analyses [6, 7] (Table 2, Fig. 3).

A final size of 768.8 Mb accounting for the 98.7% draft genome was assembled, which showed a high level of continuity with a contig N50 of 2.1 Mb and a scaffold N50 of 33.5 Mb using 1,372 contigs. The anchor rate of contigs (> 100 kb) to chromosomes was attained up to the 99.7% based on the Hi-C assembly (Table 3). The contig N50 and scaffold N50 of *O. fasciatus* were much higher than those of previous fish genome assemblies constructed using NGS DNA sequencing technologies based on the genome assembly using PacBio long reads and Hi-C

assembly (S table 3).

Genome quality evaluation

To assess the completeness of the assembled *O. fasciatus* genome, we subjected the assembled sequences to BUSCO version 3 evaluation (BUSCO, actinopterygii_odb9) [20]. Overall, 96.6% and 1.5% of the 4,584 expected actinopterygii genes were identified in the assembled genome as complete and partial BUSCO profiles, respectively. Approximately 85 genes could be considered missing in our assembly (S table 5). Among the expected complete actinopterygii genes, 4,259 and 171 were identified as single copy and duplicated BUSCOs, respectively (S table 5). We then used Minimap2 to estimate the completeness and homogeneity of genome assembly based on CLR (Continuous Long Reads) subreads. A high quality of completeness and homogeneity was assessed in the genome assembly, and the mapping rate, coverage rate and average sequencing depth reached 90.2%, 99.9% and 80.6, respectively (S table 6). Note that the mapping ratio might be related to the repetitive content of the *O. fasciatus* genome, especially for the high repeat content in the sex chromosomes[6]. However, how the repetitive elements in the genome influence the karyotypes of this species needs further investigation.

To further evaluate the accuracy of the *O. fasciatus* genome assembly, we aligned the NGS-based short reads from the whole-genome sequencing data against the reference genome using BWA[21]. We then used GATK to implement SNP calling and filter work, and the results showed that 99.8% and 0.2% of the 1.6 x 10^6 expected SNP reads were identified in the assembled genome as heterozygous and homologous SNPs, respectively. SNP calling on the final assembly also yielded a heterozygosity rate of 0.20%, supporting the *k*-mer estimate analysis (0.29%) (S table 7).

Repeat sequences within the *O. fasciatus* genome assembly

To identify tandem repeats, we utilized Tandem Repeat Finder to annotate repetitive elements in the *O. fasciatus* genome. RepeatModeler (version 1.04) and LTR_FINDER[22] were used to construct a *de novo* repeat library with default

parameters. Subsequently, we used RepeatMasker[23] (version 3.2.9) to map our assembled sequences on the Repbase TE (version 14.04) [24] and the *de novo* repeat library to identify known and novel transposable elements (TEs). In addition, TE-related proteins were annotated by using RepeatProteinMask software (version 3.2.2) [23].

The identified repeat sequences accounted for 33.9% of the *O. fasciatus* genome including repeat sequences with 23.6% of the genome based on the *de novo* repeat library (Table 4). Approximately 23.4% of the *O. fasciatus* genome was identified as interspersed repeats (most often TEs). Among them, DNA transposable elements were the most abundant type of repeat sequences, which occupied 11.5% of the whole genome. Long interspersed nuclear elements (LINEs) and long terminal repeats (LTRs) comprised 7.3% and 4.0% of the whole genome, respectively (Table 4, S Fig. 2).

RNA preparation and sequencing

We sequenced cDNA libraries prepared from the eggs of *O. fasciatus* that were used for genome annotation using Illumina sequencing technologies. RNA quality was determined based on the estimation of the ratio of absorbance at 260nm/280nm (OD = 2.0) and the RIN (value = 9.2) by using a Nanodrop ND-1000 spectrophotometer (LabTech, USA) and a 2100 Bioanalyzer (Agilent Technologies, USA), respectively. We used the Clontech SMARTer cDNA synthesis kit to complete reverse transcription. A paired-end library was prepared following the Paired-End Sample Preparation Kit manual (Illumina Inc., San Diego, CA, USA). Finally, a library with an insert length of 300 bp was sequenced by Illumina HiSeq X Ten in 150PE mode (Illumina Inc., San Diego, CA, USA). As a result, we obtained ~42.2 Gb high-quality transcriptome data from RNA-seq (S Table 1, S table 8).

Gene annotation

Gene annotation of the *O. fasciatus* genome was performed using *de novo*, homology-based and transcriptome sequencing-based predictions. We employed Augustus (version 2.5.5)[25] and GenScan (version 1.0)[26] software to predict protein-coding genes in the *O. fasciatus* genome assembly. Protein sequences of

closely related fish species including *Larimichthys crocea*，*Lates calcarifer*，*Gasterosteus aculeatus*，*Paralichthys olivaceus*，*Cynoglossus semilaevis* and *Gadus morhua* were downloaded from Ensembl[27] and aligned against the *O. fasciatus* genome using TBLASTN software[28]. Subsequently, Genewise2.2.0 software[29] was employed to predict potential gene structures on all alignments.

We also mapped these NGS transcriptome short reads onto our genome assembly using TopHat1.2 software[30], and then we employed Cufflinks[31] to predict gene structures (S table 9). All gene models were then integrated using MAKER to obtain a consensus gene set[32]. The final total gene set was composed of 24,003 genes with an average of 10.1 exons per gene in the *O. fasciatus* genome (Table 1). The gene number, gene length distribution, CDS length distribution, exon length distribution and intron length distribution were all comparable with those of other teleost fish species (S table 9, S Fig. 3).

To obtain further functional annotation of the protein-coding genes in the *O. fasciatus* genome, we employed the local BLASTX and BLASTN programs and the Swiss-prot database with an e-value ⩽ 1e-5[33] to align the non-redundant nucleotide (NT) and non-redundant protein (NR), respectively. We also used Blast2GO software to search the Gene ontology (GO), and Kyoto Encyclopaedia of Genes and Genomes (KEGG) pathway databases[34, 35, 36]. Ultimately, 97.3% (23,364 genes) of the 24,003 genes were annotated by at least one database (S Table 10). Four types of non-coding RNAs (microRNAs, transfer RNAs, ribosomal RNAs, and small nuclear RNAs) were also annotated using the tRNAscan-SE and the Rfam database in this study[37, 38] (S Table 11).

Gene family identification and phylogenetic tree construction

We employed the BLASTP program[39] with an e-value threshold of 1e-5 to identify gene families based on the transcript alignments of each gene from *O. fasciatus* and other fish species, which included *Larimichthys crocea*, *Gadus morhua*, *Paralichthys olivaceus*, *Cynoglossus semilaevis*, *Notothenia coriiceps*, *Boleophthalmus pectinirostris*, *Lepisosteus oculatus*, *Gasterosteus aculeatus*, *Callorhinchus milii*, *Danio rerio*, *Salmo salar* and *Oryzias latipes*. 21,528 gene families were identified by

clustering the homologous gene sequences based on H-scores calculated from Bit-score using Hcluster_sg software (S Fig. 4). Subsequently, we selected 1,236 single-copy orthogroups from the above-mentioned species to construct the phylogenetic relationship between *O. fasciatus* and other fish species. We used the ClustalW program[40] to extract and align coding sequences of single-copy genes from the 1,158 orthogroups with a length filter (S Fig. 5). All the alignments were concatenated as a single data set for each species. Nondegenerated sites extracted from the data set were then joined into new sequences for each species to construct a phylogenetic tree based on the maximum-likelihood method implemented in the PhyML package[41] (with the -m PROTGAMMAAUTO model). We used the MCMCtree program to estimate divergence times among species based on the approximate likelihood method[42] and a molecular clock data from the divergence time between medaka from the TimeTree database[43]. According to the phylogenetic analysis, *O. fasciatus* (Eupercaria: Centrarchiformes) clustered with *Larimichthys crocea* in the order Perciformes (Eupercaria), which was consistent with the new fish species taxonomy[44] (Fig. 4). The divergence time between *O. fasciatus* and the common ancestor with *Larimichthys crocea* was at approximately 70.5-88.5 Ma.

Conclusions

We successfully assembled the genome of *O. fasciatus* and reported the first chromosome-level genome sequencing, assembly and annotation based on long reads from the third-generation PacBio Sequel sequencing platform. The final draft genome assembly is approximately 778.7 Mb, which was slightly higher than the estimated genome size (777.5 Mb) based on *k*-mer analysis. Those contigs were scaffolded to chromosomes using Hi-C data, resulting a genome with a high level of continuity with a contig N50 of 2.1 Mb and a scaffold N50 of 33.5 Mb. The chromosome-level genome assembly of *O. fasciatus* was the first high-quality genome in the Oplegnathidae family. We also predicated 24,003 protein-coding genes from the generated assembly, and 97.3% (23,364 genes) of all protein-coding genes were annotated. We found that the divergence time between *O. fasciatus* and its the common ancestor with *Larimichthys crocea* was approximately 70.5-88.5 Ma. As far

as we known, the Y chromosomes has always exhibited many specific sequence characteristics compared to X1 and X2, such as repeat content, and those differences might increase the difficulty of the sequence assembly of chromosomes X1 and X2. The chromosome-level genome assembly together with gene annotation data generated for the female fish in this work will provide a valuable resource for further research on sex-determining mechanisms, especially for obtaining an accurate assembly of the Y chromosome in male fish. These results will also accelerate genome-wide association studies in resistant breeding systems.

Ethics Statement

This research was approved by the Animal Care and Use committee of Chinese Academic Science. All participates consent the study under the 'Ethics, consent and permissions' heading. All participants consent to publish the work under the 'Consent to publish' heading.

Availability of supporting data

Supporting data and materials are available in the GigaScience GigaDB database, with the raw sequences deposited in the SRA under the accession number SRP158313 and SRP160016.

Competing interests

The authors declare that they have no competing interests.

Funding

This study was supported by a grant from the National Natural Science Foundation of China (No. 41506170, No. 31672672, and No. 31872195), Shandong Province Key Research and Invention Program (2017GHY15102, 2017GHY15106), Qingdao Source Innovation Program (17-1-1-57-jch), STS (2017, 2018), Marine Fishery Institute of Zhejiang Province, Key Laboratory of Mariculture and Enhancement of Zhejiang Province (2016KF002). Qingdao National Laboratory for Marine Science

and Technology (2015ASKJ02, 2015ASKJ02-03-03), China Agriculture Research System (CARS-47), STS project (KFZD-SW-106, ZSSD-019).

Author Contributions

YSX conceived the project. ZZX, DYM collected the samples and extracted the genomic DNA. YSX, JL and JL performed the genome assembly and data analysis. YSX, ZZX, JL, DYM and JL wrote the paper.

Reference

1. Schembri, P.J. *et al*. Occurrence of barred kinfejaw, *Oplegnathuf fasciatus* (Actinopterygii: Perciformes: Oplegnathidae), in Malta (Central Mediterranean) with a discussion on possible modes of entry. *Acta Ichthyol Piscat* 40,101-104 (2010).

2. Mundy, B.C. Checklist of the fishes of the Hawaiian Archipelago. *Bishop Mus Bull Zool* 6, 1-704 (2005).

3. An, H.S. & Hong, S.W. Genetic diversity of rock bream *Oplegnathus fasciatus* in Southern Korea. *Genes Genom* 30, 451-459 (2008).

4. Xiao, Y.S. *et al*. Pronounced population genetic differentiation in the rock bream *Oplegnathus fasciatus* inferred from mitochondrial DNA sequences. *Mitochondrial DNA A* 27, 2045-2052 (2016).

5. Park, H.S. *et al*. Population Genetic Structure of Rock Bream (*Oplegnathus fasciatus* Temminck & Schlegel, 1884) Revealed by mtDNA COI Sequence in Korea and China. Ocean Sci J 53, 261-274 (2018).

6. Xu, D.D. *et al*. Chromosomal mapping of microsatellite repeats in the rock bream fish *Oplegnathus fasciatus*, with emphasis of their distribution in the neo-Y chromosome. *Mol Cytogenet* 6, 12 (2013).

7. Xue, R. *et al.* Karyotype and Ag-Nors In Male And Female Of *Oplegnathus Punctatus*.

Oceanol Limnol Sin 47, 626-632 (2016).

8. Xiao, Z.Z. Study on population genetics and culture biology of *Oplegnathus fasciatus*. Doctor thesis p 162-176 (2015).
9. Zhang, B.C. *et al*. Rock bream (*Oplegnathus fasciatus*) viperin is a virus-responsive protein that modulates innate immunity and promotes resistance against megalocytivirus infection. *Dev Comp Immunol* 45, 35-42 (2014).
10. L, H. *et al*. Characterization of an Iridovirus Detected in Rock Bream (Oplegnathus fasciatus ;Temminck and Schlegel). *Chin J Virol* 27, 158-164 (2011).
11. Koren, S. *et al.* Canu: scalable and accurate long-read assembly via adaptive k-mer weighting and repeat separation. *Genome Res* 27, 722 (2017).
12. Marcais, G. & Kingsford, C. A fast, lock-free approach for efficient parallel counting of occurrences of k-mers. *Bioinformatics* 27, 764–70 (2011).
13. Kajitani, R. *et al.* Efficient de novo assembly of highly heterozygous genomes from whole-genome shotgun short reads. *Genome Res* 24, 1384-1395 (2014).
14. Pryszcz, L. P. & Gabaldón, T. Redundans: an assembly pipeline for highly heterozygous genomes. *Nucleic Acids Res* 44, e113-e113 (2016).
15. Simão, F. A. *et al*. BUSCO: assessing genome assembly and annotation completeness with single-copy orthologs. *Bioinformatics* 31, 3210 (2015).
16. Li, H. & Durbin, R. Fast and accurate short read alignment with Burrows–Wheeler transform. *Bioinformatics* 25, 1754-1760 (2009).
17. Benson, G. Tandem repeats finder: a program to analyze DNA sequences. *Nucleic Acids Res* 27, 573–80 (1999).
18. Tarailo-Graovac, M. & Chen, N. Using RepeatMasker to identify repetitive elements in genomic sequences. In: Editoral board, Baxevanis Andreas D et al. (eds.), Current Protocols in Bioinformatics, Chapter 4:Unit 4 10 (2009).
19. Jurka, J. *et al.* Repbase Update, a database of eukaryotic repetitive elements. Cytogenetic Genome Res 110, 462–67 (2005).
20. Stanke, M. *et al*. AUGUSTUS: a web server for gene finding in eukaryotes. *Nucleic Acids Res* 32(Web Server issue), W309-12 (2004).
21. Cai, Y. *et al.* Computational systems biology methods in molecular biology, chemistry

biology, molecular biomedicine, and biopharmacy. *BioMed Res Int* 2014, 746814 (2014).

22. Flicek, P. *et al*. Ensembl 2014. *Nucleic Acids Res* 42, D749-D755 (2014).
23. Gertz, E. M. *et al.* Composition-based statistics and translated nucleotide searches: Improving the TBLASTN module of BLAST. *MBC Biology* 4, 41 (2006).
24. Birney, E. et al. GeneWise and Genomewise. *Genome Res* 14, 988-995 (2004).
25. Trapnell, C. *et al.* TopHat: discovering splice junctions with RNA-Seq. *Bioinformatics* 25, 105-1111 (2009).
26. Ghosh, S. & Chan, C. K. K. Analysis of RNA-Seq data using TopHat and Cufflinks. *Methods Mole Biol* 1374, 339 (2016).
27. Campbell, M. S. *et al*. Genome Annotation and Curation Using MAKER and MAKER-P. *Current Protocols in Bioinformatics* 48, 4.11.11 (2014).
28. Lobo, I. Basic Local Alignment Search Tool (BLAST). *J Mol Biol* 215, 403-410 (2008).
29. Harris, M. A. *et al.* The Gene Ontology (GO) database and informatics resource. *Nucleic Acids Res* (2004).
30. Ogata, H. *et al.* KEGG: Kyoto Encyclopedia of Genes and Genomes. *Nucleic Acids Res* 27, 29-34 (2000).
31. Conesa, A. *et al.* Blast2GO: a universal tool for annotation, visualization and analysis in functional genomics research. *Bioinformatics* 21, 3674 (2005).
32. Lowe, T. M. & Eddy, S. R. tRNAscan-SE: a program for improved detection of transfer RNA genes in genomic sequence. *Nucleic Acids Res* 25, 955-964 (1997).
33. Griffithsjones, S. *et al.* Rfam: an RNA family database. *Nucleic Acids Res* 31, 439 (2003).
34. Lieberman-Aiden, E. *et al*. Comprehensive mapping of long-range interactions reveals folding principles of the human genome. *Science* 326, 289-293 (2009).
35. Rao, S.S.P. *et al.* A 3D map of the human genome at kilobase resolution reveals principles of chromatin looping. *Cell* 159, 1665-1680 (2014).
36. Sandborn, A.L. *et al*. Chromatin extrusion explains key features of loop and domain formation in wild-type and engineered genomes. *Proc Natl Acad Sci USA* 112, E6456 (2015).
37. Flot, J.F. *et al*. Contact genomics: scaffolding and phasing (meta) genomes using chromosome be 3D physical signatures. *FEBs Letters* 589, 2966-2974 (2015).
38. Burton, J.N. *et al*. Chromosome-scale scaffolding of de novo genome assemblies based on

chromatin interactions. *Nat Biotechnol* 31, 1119-1125 (2013).

39. Lobo, I. Basic Local Alignment Search Tool (BLAST). J Mol Biol 215, 403-410 (2008).

40. Thompson, J. D. *et al.* Multiple sequence alignment using ClustalW and ClustalX. *Curr Protoc Bioinformatics* 2.3. 1-2.3. 22 (2002).

41. Guindon, S. *et al.* PhyML: Fast and Accurate Phylogeny Reconstruction by Maximum Likelihood. *Infect Genet Evol* 9, 384-385 (2009).

42. Yang, Z. & Rannala, B. Bayesian estimation of species divergence times under a molecular clock using multiple fossil calibrations with soft bounds. *Mol Biol Evol* 23, 212-226 (2006).

43. Hedges, S. B. *et al.* Tree of life reveals clock-like speciation and diversification. *Mol Biol Evol* 32, 835-845 (2015).

44. Ricardo, B. R. *et al*. Phylogenetic classification of bony fishes. BMC Evol Biol 17, 162 (2017).

Manuscript-tracked changes
Click here to access/download;Manuscript;Manuscript-tracked changes.pdf
Click here to view linked References

Genome sequence of the barred knifejaw Oplegnathus fasciatus (Temminck & Schlegel, 1884): the first chromosome-level draft genome in the family Oplegnathidae

删除的内容: rock bream

删除的内容: ,

Yongshuang Xiao[1,2,3*]†, Zhizhong Xiao[1,2,3,]†, Daoyuan Ma[1,2,3], Jing Liu[2,3*], Jun Li[1,2,3*]

[1]CAS Key Laboratory of Experimental Marine Biology, Institute of Oceanology, Chinese Academy of Sciences, 7 Nanhai Road, Qingdao, 266071, China, [2] Laboratory for Marine Biology and Biotechnology, Qingdao National Laboratory for Marine Science and Technology, 7 Nanhai Road, Qingdao, 266071, China, [3]Center for Ocean Mega-Science, Chinese Academy of Sciences, 7 Nanhai Road, Qingdao, 266071, China

*Correspondence address: Yongshuang Xiao, Mega-Science, Chinese Academy of Sciences, 7 Nanhai Road, Qingdao, 266071, China; Tel: +86-053282896729; E-mail: dahaishuang1982@163.com; Jing Liu, Institute of Oceanology, Chinese Academy of Sciences, 7 Nanhai Road, Qingdao, 266071, China; Tel: +86-053282898790; E-mail: jliu@qdio.ac.cn; Jun Li, Institute of Oceanology, Chinese Academy of Sciences, 7 Nanhai Road, Qingdao, 266071, China; Tel: +86-053282898718; E-mail: junli@qdio.ac.cn.

†Contributed equally to this work.

Abstract

Background

The barred knifejaw (*Oplegnathus fasciatus*), a member of the Oplegnathidae family of the Centrarchiformes, is a commercially important rocky reef fish native to East Asia. *O. fasciatus* has become an important fishery resource for offshore cage aquaculture and fish stocking of marine ranching in China, Japan and Korea. Recently, sexual dimorphism in growth with neo-sex chromosome and widespread biotic diseases in *O. fasciatus* has been received increasing concern. However, adequate genome resources for gaining insight into sex-determining mechanisms and establishing genetically resistant breeding systems for *O. fasciatus* are lacking. Here, we analysed the entire genome of a female *O. fasciatus* fish using long-read sequencing and Hi-C data to generate chromosome-length scaffolds and a highly contiguous genome assembly.

Findings

We assembled the *O. fasciatus* genome with a total of 245.0 Gb of raw reads that were generated using both of PacBio Sequel and Illumina HiSeq 2000 platforms. The final draft genome assembly was approximately 778.7 Mb, which reached a high level of continuity with a contig N50 of 2.1 Mb. The genome size was consistent with the estimated genome size (777.5 Mb) based on *k*-mer analysis. We combined Hi-C data with a draft genome assembly to generate chromosome-length scaffolds. Twenty-four scaffolds corresponding to the twenty-four chromosomes were assembled to a final size of 768.8 Mb with a contig N50 of 2.1 Mb and a scaffold N50 of 33.5 Mb using 1,372 contigs. The identified repeat sequences accounted for 33.9% of the entire genome, and 24,003 protein-coding genes with an average of 10.1 exons per gene were annotated using *de novo* methods, with RNA-seq data and homologies to other teleosts. According to phylogenetic analysis using protein-coding genes, *O. fasciatus* is closely related to *Larimichthys crocea*, with *O. fasciatus* diverging from their common ancestor approximately 70.5-88.5 million years ago.

Conclusions

We generated a high-quality draft genome with chromosome assembly for *O.*

删除的内容: .
删除的内容: rock bream
删除的内容: Perciformes… is a commerically
删除的内容: growth of …exual dimorphism in growth with
删除的内容: to
删除的内容: basing
删除的内容: based
删除的内容: have been…re lacking. Here, we performed
删除的内容: fish for …. *fasciatus* fish using long-read
删除的内容: , which
删除的内容: Hiseq
删除的内容: remarkable …igh level of continuity with a
删除的内容: 32.2
删除的内容: whole …ntire genome, and 24 …4,003
删除的内容: the …hylogenetic analysis using protein-coding
带格式的: 非突出显示
删除的内容: 87
带格式的: 非突出显示
删除的内容: 3
删除的内容: and…with chromosomes

fasciatus using long reads generated using PacBio sequencing technologies, which represents the first chromosome-level reference genome for Oplegnathidae species. Assembly of this genome will provide insight into sex-determining mechanisms and serve as a resource for accelerating genome-assisted improvements in resistant breeding systems.

Keywords: *Oplegnathus fasciatus*; chromosome-level genome assembly; Hi-C assembly; sex-determining mechanism

Data description

Introduction of *O. fasciatus*

The Oplegnathidae family belongs to the order Centrarchiformes, including only one genus *Oplegnathus*, which is comprised of seven species (*O. conwayi, O. fasciatus, O. insignis, O. peaolopesi, O. punctatus, O. robinsoni, O. woodwardi*), two of which (*O. fasciatus* and *O. punctatus*) are commercially valuable in East Asia. The barred knifejaw *O. fasciatus* (Temminck & Schlegel, 1844) is one of these two species in the *Oplegnathus*, which is commonly found at the depth of one to ten metres in association with rocky reefs[1, 2], and distributed across a wide range of shallow waters around Korea, Japan, China and Hawaii[1, 3, 4] (Fig. 1). *O. fasciatus* has become an important fishery resource for offshore cage aquaculture and fish stocking of marine ranching in China, Japan and Korea[5]. It has been reported that the male of *Oplegnathus* possesses a neo-sex chromosome, possibly a sex chromosome Y. The sex chromosome system for *Oplegnathus* is considered to be $X_1 X_1 X_2 X_2$ / $X_1 X_2$ Y based on karyotype analyses[6, 7]. Furthermore, sexual dimorphism in growth has been detected in the *O. fasciatus*, with male fish exhibiting faster growth than females, possibly be due to the sex chromosome system in *Oplegnathus*[8]. *O. fasciatus* is vulnerable to viruses (e.g., Iridovirus) and genetic degradation caused by inbreeding has led to higher susceptibility to diseases[9, 10]. It is vital to develop genomic resources to gain insight into sex-determining mechanisms and to accelerate the genome-assisted improvements in resistant breeding systems.

So far, a genome sequence with the chromosomal assembly of *O. fasciatus* has

删除的内容: is
删除的内容: The genome assembly
删除的内容: the …enome-assisted improvements of
删除的内容: The family Oplegnathidae belongs…to the order
删除的内容: two
已下移 [2]: (*O. fasciatus* and *O. punctatus*)
已移动(插入) [2]
删除的内容:
删除的内容: that …re of
删除的内容: values …aluable in East Asis
删除的内容: rock bream
删除的内容: ,…*O. fasciatus* (Temminck & Schlegel,
删除的内容: has …ossesses a neo-sex chromosome, possibly
删除的内容: was …s considered to be $X_1 X_1 X_2 X_2$ / X_1 [illegible]
删除的内容: the growth
删除的内容: was …as been detected in the *O. fasciatus*
删除的内容: the … genome sequence and …with the
删除的内容: have

not been reported. Here, we constructed a high-quality chromosome-level reference genome assembly for *O. fasciatus* using long reads by using the PacBio DNA sequencing platform and used a genome assembly strategy by taking advantage of genome assembly program Canu[11]. This genome assembly of *O. fasciatus* is the first chromosome-level reference genome constructed for the Oplegnathidae family. The completeness and continuity of the genome will provide high quality genomic resources for studies on sex-determining mechanisms and for accelerating the genome-assisted improvements in resistant breeding systems.

Genomic DNA extraction, genome size estimation

High-quality genomic DNA for sequencing using the Illumina platform (Illumina Inc., San Diego, CA, USA) and PacBio Sequel sequencing (Pacific Biosciences of California, Menlo Park, CA, USA) was extracted from fresh muscle tissue and blood samples from a single female *O. fasciatus*. The fish was collected from the near-shore area of Qingdao city (Yellow Sea), Shandong province. The whole-genome size of *O. fasciatus* was estimated based on Illumina DNA sequencing technology. A short-insert library (300~350 bp) was constructed and generated a total of ~90.7 Gb of raw reads using the standard protocol provided by the Illumina HiSeq X Ten platform (Illumina Inc., San Diego, CA, USA). After the removal of low-quality and redundant reads, we obtained approximately ~80.8 Gb of clean data for *de novo* assembly to estimate the whole-genome size (S Table 1, Fig. 2). All cleaned reads were subjected to 17-mer frequency distribution analysis[12]. As the total number of *k*-mers was approximately 8.09 x 10^{10} and the peak of *k*-mers was at a depth of 100, the genome size of *O. fasciatus* was calculated to be 777.5 Mb using the following formula with amendment: genome size = *k*-mer number / peak depth (Fig. 2). Meanwhile, an estimated heterozygosity of 0.29% and a repeat content of 38.46% were detected for *O. fasciatus* in this work. A pilot genome assembly was approximately 744.5 Mb with a contig N50 of 7.2 kb and a scaffold N50 of 84.1kb using the Illumina data and the assembly program Platanus package[13] (S Table 2). The GC content was 41% (S Fig. 1). This genome assembly was of low-quality, partly due to its high genomic repeat

删除的内容: performed
删除的内容: constructed
删除的内容: ,
删除的内容: using
删除的内容: assemblyer
删除的内容: The
删除的内容: Oplegnathidae
删除的内容: of
删除的内容: **and Hi-C library construction**
删除的内容: of
带格式的: 字体: 倾斜
删除的内容: A whole-genome using Illumina DNA sequencing technology was applied to estimate *O. fasciatus* genome size
删除的内容: about
删除的内容: the
删除的内容: about
删除的内容: 808.9
删除的内容: the
删除的内容: 808.9
删除的内容: 777.5
删除的内容: s

content.

Genome assembly using PacBio long reads

Two 20 kb genomic DNA libraries were constructed and sequenced using the PacBio Sequel platform, generating 62.9 Gb raw DNA reads. We obtained 4.8 million subreads (62.8 Gb in total) with an N50 read length of ~22 kb after removing adaptor (S Table 1).

Canu v1.4 was firstly used to assemble the genome with the Corrected-Error-Rate parameter set at 0.040[11]. As a result, a genome assembly with a total length of 875.9 Mb was constructed for *O. fasciatus*, slightly higher than the genome size estimated by 17-mer analysis based on the Illumina data (S Table 2). We attributed the relatively large genome size of the assembly to the complexity of this genome to factors such as heterozygosity. We therefore applied Redundans v0.13c[14] to remove the sequence redundancy to obtain a genome assembly size of 778.0 Mb. We then used the Arrow tool in SMRT Link 5.0 software with the minCoverage parameter set at 15 to implement error correction based on the PacBio long reads data (Table 1). The resulting genome assembly was further polished using Illumina NGS data, which were used in the genome survey analysis above. The final draft genome assembly was 778.7 Mb, which reached a high level of continuity with a contig N50 length of 2.1 Mb (Table 1). The contig N50 of *O. fasciatus* was much higher than those of previous fish genome assemblies constructed using NGS DNA sequencing technologies and is comparable to those of recently reported model fish species (S table 3). Previous studies illuminated the relationship between read length and genome assembly; therefore, we attributed the continuity of the genome primarily to the application of long reads in the assembly.

Hi-C library construction and chromosome assembly

Hi-C is a sequencing-based approach for determining chromosome interactions by calculating the contact frequency between pairs of loci, which are strongly dependent upon the one-dimensional distance, in base pairs, between a pair of loci[15, 16]. In this work, we used Hi-C to construct the genome assembly of *O. fasciatus*.

删除的内容: The genomic DNA for Hi-C library was extracted from the whole-blood cell of *O. fasciatus* as described[14]. The cells were fixed with formaldehyde and lysed, and the cross-linked DNA digested with MboI. Sticky ends were biotin-labeled and proximity ligated to form chimeric junctions that were enriched for and then physically sheared to a size of 300–500 bp[14]. Chimeric fragments representing the original cross-linked long-distance physical interactions were then processed into paired-end sequencing libraries, and 629 million 150-bp paired-end Illumina reads (91.5 Gb) with Q20 and Q30 of ~94.0% were produced (S Table 1, S Table 3). As a result, the paired data, data with mate mapped to a different contig (or scaffold) and data with mapped to a different contig (or scaffold) (map Q5≥ 5) were 593.7 Mb (94.4%), 240.5 Mb (40.5%) and 205.1 Mb (34.6%), respectively (S Table 3). .

带格式的: 字体颜色: 红色

带格式的: 字体颜色: 红色

带格式的: 字体颜色: 红色

带格式的: 字体颜色: 红色

删除的内容: totally 62.8 Gb

删除的内容: a read N50

删除的内容: The

删除的内容: genome assembly

删除的内容: achieved

删除的内容: which was

删除的内容: consistent with

删除的内容: genome size in

删除的内容: [15]

删除的内容: and

删除的内容: the Arrow of Smrtlink 5.0

删除的内容: the

删除的内容: remarkable

删除的内容: with

删除的内容: ,

删除的内容: with

删除的内容: S Table 4

Genomic DNA was extracted for the Hi-C library from a whole-blood sample of *O. fasciatus* as described[17]. Cells were fixed with formaldehyde and lysed, and the cross-linked DNA was digested with MboI. Sticky ends were biotin-labelled and proximity ligated to form chimeric junctions and then physically sheared to a size of 300–500 bp[17]. Chimeric fragments representing the original cross-linked, long-distance physical interactions were then processed into paired-end sequencing libraries, and 629 million 150-bp paired-end Illumina reads (91.5 Gb) were produced with Q20 and Q30 of ~94.0% (S Table 1, S Table 4). By mapping the Hi-C data to the PacBio-based assembly using BWA software, we found that sequencing data with mates mapped to a different contig (or scaffold) and data mapped to a different contig (or scaffold) (map Q5⩾ 5) were 593.7 Mb (94.4%), 240.5 Mb (40.5%) and 205.1 Mb (34.6%), respectively (S Table 4). We then further employed BWA and Lachesis software to align paired-end reads to filter all base sequences more than 500bp from each restriction site[18]. According to the conduct of clustering, ordering, and orienting to the assembly contigs (1 692), these sequences were grouped into 24 chromosome clusters and scaffolded using Lachesis software with tuned parameters[19] (Table 2, Fig. 3). Finally, we constructed the chromosome interactions map using Juicer software and employed the JucieBox to complete the visual correction of the interaction map. We obtained 1 756 polished contigs by interrupting misassembly from 1 692 contigs. Twenty-four scaffolds were assembled corresponding to the 24 chromosomes of *O. fasciatus* based on the karyotype analyses [6, 7] (Table 2, Fig. 3).

带格式的：非突出显示

带格式的：非突出显示

A final size of 768.8 Mb accounting for the 98.7% draft genome was assembled, which showed a high level of continuity with a contig N50 of 2.1 Mb and a scaffold N50 of 33.5 Mb using 1372 contigs. The anchor rate of contigs (> 100 kb) to chromosomes was attained up to the 99.7% based on the Hi-C assembly (Table 4). The contig N50 and scaffold N50 of *O. fasciatus* were much higher than those of previous fish genome assemblies constructed using NGS DNA sequencing technologies based on the genome assembly using PacBio long reads and Hi-C assembly (S table 3).

Genome quality evaluation

To assess the completeness of the assembled *O. fasciatus* genome, we subjected the assembled sequences to BUSCO version 3 evaluation (BUSCO, actinopterygii_odb9) [20]. Overall, 96.6% and 1.5% of the 4 584 expected actinopterygii genes were identified in the assembled genome as complete and partial BUSCO profiles, respectively. Approximately 85 genes could be considered missing in our assembly (S table 5). Among the expected complete actinopterygii genes, 4 259 and 171 were identified as single copy and duplicated BUSCOs, respectively (S table 5). We then used Minimap2 to estimate the completeness and homogeneity of genome assembly based on CLR (Continuous Long Reads) subreads. A high quality of completeness and homogeneity was assessed in the genome assembly, and the mapping rate, coverage rate and average sequencing depth reached 90.2%, 99.9% and 80.6, respectively (S table 6). Note that the mapping ratio might be related to the repetitive content of the *O. fasciatus* genome, especially for the high repeat content in the sex chromosomes[6]. However, how the repetitive elements in the genome influence the karyotypes of this species needs further investigation.

To further evaluate the accuracy of the *O. fasciatus* genome assembly, we aligned the NGS-based short reads from the whole-genome sequencing data against the reference genome using BWA[21]. We then used GATK to implement SNP calling and filter work, and the results showed that 99.8% and 0.2% of the 1.6 x 10^6 expected SNP reads were identified in the assembled genome as heterozygous and homologous SNPs, respectively. SNP calling on the final assembly also yielded a heterozygosity rate of 0.20%, supporting the *k*-mer estimate analysis (0.29%) (S table 7).

Repeat sequences within the *O. fasciatus* genome assembly

To identify tandem repeats, we utilized Tandem Repeat Finder to annotate repetitive elements in the *O. fasciatus* genome. RepeatModeler (version 1.04) and LTR_FINDER[22] were used to construct a *de novo* repeat library with default parameters. Subsequently, we used RepeatMasker[23] (version 3.2.9) to map our assembled sequences on the Repbase TE (version 14.04) [24] and the *de novo* repeat

删除的内容: [16]
删除的内容:
删除的内容: S Table 5
删除的内容: both of
删除的内容: S Table 5
删除的内容: the
删除的内容: the
删除的内容: checked for
删除的内容: were
删除的内容: to
删除的内容: S Table 6
带格式的：上标
删除的内容: BWA[17]
删除的内容: the
删除的内容: the
删除的内容:
删除的内容: heterozygosis and homology
删除的内容: the estimate from *k*-mer
删除的内容: S Table 7
删除的内容: [18]
删除的内容: r
删除的内容: [1]
删除的内容: [9]
删除的内容: [20]

library to identify known and novel transposable elements (TEs). In addition, TE-related proteins were annotated by using RepeatProteinMask software (version 3.2.2)[23].

The identified repeat sequences accounted for 33.9% of the *O. fasciatus* genome including repeat sequences with 23.6% of the genome based on the *de novo* repeat library (Table 4). Approximately 23.4% of the *O. fasciatus* genome was identified as interspersed repeats (most often TEs). Among them, DNA transposable elements were the most abundant type of repeat sequences, which occupied 11.5% of the whole genome. Long interspersed nuclear elements (LINEs) and long terminal repeats (LTRs) comprised 7.3% and 4.0% of the whole genome, respectively (Table 4, S Fig. 2).

RNA preparation and sequencing

We sequenced cDNA libraries prepared from the eggs of *O. fasciatus* that were used for genome annotation using Illumina sequencing technologies. RNA quality was determined based on the estimation of the ratio of absorbance at 260nm/280nm (OD = 2.0) and the RIN (value = 9.2) by using a Nanodrop ND-1000 spectrophotometer (LabTech, USA) and a 2100 Bioanalyzer (Agilent Technologies, USA), respectively. We used the Clontech SMARTer cDNA synthesis kit to complete reverse transcription. A paired-end library was prepared following the Paired-End Sample Preparation Kit manual (Illumina Inc., San Diego, CA, USA). Finally, a library with an insert length of 300 bp was sequenced by Illumina HiSeq X Ten in 150PE mode (Illumina Inc., San Diego, CA, USA). As a result, we obtained ~42.2 Gb high-quality transcriptome data from RNA-seq (S Table 1, S table 8).

Gene annotation

Gene annotation of the *O. fasciatus* genome was performed using *de novo*, homology-based and transcriptome sequencing-based predictions. We employed Augustus (version 2.5.5)[25] and GenScan (version 1.0)[26] software to predict protein-coding genes in the *O. fasciatus* genome assembly. Protein sequences of closely related fish species including *Larimichthys crocea*, *Lates calcarifer*, *Gasterosteus aculeatus*, *Paralichthys olivaceus*, *Cynoglossus semilaevis* and *Gadus morhua* were downloaded from Ensembl[27] and aligned against the *O. fasciatus*

删除的内容: the
删除的内容: [1]
删除的内容: [9]
删除的内容: ed
删除的内容: The total identified
删除的内容: accounted for
删除的内容: *O. fasciatus*
删除的内容: 2
删除的内容: 1
删除的内容: The l
删除的内容: took up
删除的内容: 2
删除的内容: High quality of RNA were detected
删除的内容:
删除的内容:
删除的内容: the process of
删除的内容: The
删除的内容: the manual of
删除的内容: the
删除的内容: S Table 8
删除的内容: [21]
删除的内容: [22]
删除的内容: s
删除的内容: of
删除的内容: [23]
删除的内容: to

genome using TBLASTN software[28]. Subsequently, Genewise2.2.0 software[29] was employed to predict potential gene structures on all alignments.

We also mapped these NGS transcriptome short reads onto our genome assembly using TopHat1.2 software[30], and we then employed Cufflinks[31] to predict gene structures (S table 9). All gene models were then integrated using MAKER to obtain a consensus gene set[32]. The final total gene set was composed of 24 003 genes with an average of 10.1 exons per gene in the *O. fasciatus* genome (Table 1). The gene number, gene length distribution, CDS length distribution, exon length distribution and intron length distribution were all comparable with those of other teleost fish species (S table 9, S Fig. 3).

To obtain further functional annotation of the protein-coding genes in the *O. fasciatus* genome, we employed the local BLASTX and BLASTN programs and the Swiss-prot database with an e-value ≤ 1e-5[33] to align the non-redundant nucleotide (NT) and non-redundant protein (NR), respectively. We also used Blast2GO software to search the Gene ontology (GO), and Kyoto Encyclopaedia of Genes and Genomes (KEGG) pathway databases[34, 35, 36]. Ultimately, 97.3% (23 364 genes) of the 24 003 genes were annotated by at least one database (S Table 10). Four types of non-coding RNAs (microRNAs, transfer RNAs, ribosomal RNAs, and small nuclear RNAs) were also annotated using the tRNAscan-SE and the Rfam database in this study[37, 38] (S Table 11).

Gene family identification and phylogenetic tree construction

We employed the BLASTP program[39] with an e-value threshold of 1e-5 to identify gene families based on the transcript alignments of each gene from *O. fasciatus* and other fish species, which included *Larimichthys crocea*, *Gadus morhua*, *Paralichthys olivaceus*, *Cynoglossus semilaevis*, *Notothenia coriiceps*, *Boleophthalmus pectinirostris*, *Lepisosteus oculatus*, *Gasterosteus aculeatus*, *Callorhinchus milii*, *Danio rerio*, *Salmo salar* and *Oryzias latipes*. 21,528 gene families were identified by clustering the homologous gene sequences based on H-scores calculated from Bit-score using Hcluster_sg software (S Fig. 4). Subsequently, we selected 1,236 single-copy orthogroups from the above-mentioned species to construct the

删除的内容: [24…8]. Subsequently, Genewise2.2.0 software[25…9] ...

删除的内容: [26…0], and we then we …mployed Cufflinks[27…1] ...

删除的内容: in…of other teleost fish species (S Table 9 ...

删除的内容: In order t…o o…btain further ...

删除的内容: **Hi-C assembly and chromosome interactions** Hi-C was a sequencing-based approach for determining chromosome interactions by calculating the contact frequency between pairs of loci, which depended strongly on the one-dimensional distance, in base pairs, between a pair of loci[35, 36]. We employed BWA and Lachesis softwares to align paired-end reads to the draft genome assembly and filtered all base sequences other than 500bp from each restriction site[37]. According to the conduct of clustering, ordering, and orienting to the assembly contigs (1 692), those were grouped into 24 chromosome clusters and scaffolded using Lachesis software with tuned parameters[38] (Table 3, Fig. 3). Finally, we constructed the chromosome interactions map using Juicer software and employed the JucieBox to complete the visual correction of interactions map. We obtained polished 1 756 contigs by interrupting misassembly from the 1 692 contigs. Twenty-four scaffolds corresponding to the 24 chromosomes of *O. fasciatus* based on the karyotype analyses were assembled[6, 7] (Table 3, Fig. 3). A final size of 768.8 Mb accounting for the 98.7% draft genome was assembled, which remarkable high level of continuity with contig N50 of 2.1 Mb and scaffold N50 of 33.5 Mb using 1372 contigs. The anchor rate of contigs (> 100 kb) to chromosomes was reached up to the 99.7% based on the Hi-C assembly (Table 4). The contig N50 and scaffold N50 of *O. fasciatus* were much higher than those of previous fish genome assemblies constructed using NGS DNA sequencing technologies based on the genome assembly using PacBio long reads and Hi-C assembly (S Table 4). .

删除的内容: family…based on the transcripts…alignments ...

带格式的 ...

删除的内容: 23273

删除的内容: 23,270

删除的内容: of …he homologous gene sequences based on ...

phylogenetic relationships between *O. fasciatus* and other fish species. We used the ClustalW program[40] to extract and align coding sequences of single-copy genes from the 1,158 orthogroups with a length filter (S Fig. 5). All the alignments were concatenated as a single data set for each species. Nondegenerated sites extracted from the data set were then joined into new sequences for each species to construct a phylogenetic tree based on the maximum-likelihood method implemented in the PhyML package[41] (with the -m PROTGAMMAAUTO model). We used the MCMCtree program to estimate divergence times among species based on the approximate likelihood method[42] and molecular clock data from the divergence time between medaka from the TimeTree database[43]. According to the phylogenetic analysis *O. fasciatus* (Eupercaria: Centrarchiformes) clustered with *Larimichthys crocea* in the order Perciformes (Eupercaria), which was consistent with the new fish species taxonomy[44] (Fig. 4). The divergence time between *O. fasciatus* and the common ancestor with *Larimichthys crocea* was approximately 70.5-88.5 Ma.

Conclusions

We successfully assembled the genome of *O. fasciatus* and reported the first chromosome-level genome sequencing, assembly and annotation based on long reads from the third-generation PacBio Sequel sequencing platform. The final draft genome assembly is approximately 778.7 Mb, which was slightly higher than the estimated genome size (777.5 Mb) based on *k*-mer analysis. Those contigs were scaffolded to chromosomes using Hi-C data, resulting a genome with a high level of continuity with a contig N50 of 2.1 Mb and a scaffold N50 of 33.5 Mb. The chromosome-level genome assembly of *O. fasciatus* was the first high-quality genome in the Oplegnathidae family. We also predicated 24 003 protein-coding genes from the generated assembly, and 97.3% (23 364 genes) of all protein-coding genes were annotated. We found that the divergence time between *O. fasciatus* and its the common ancestor with *Larimichthys crocea* was approximately 70.5-88.5 Ma. As far as we known, the Y chromosomes has always exhibited many specific sequence characteristics compared to X1 and X2, such as repeat content, and those differences might increase the difficulty of the sequence assembly of chromosomes X1 and X2.

删除的内容: the

删除的内容:

删除的内容: 765

删除的内容: , respectively

删除的内容: of

删除的内容: a

删除的内容: were

删除的内容: together

删除的内容: belonged to

删除的内容: . The taxonomy of Notothenioidei should be elevated to the order level from the Perciformes and be paralleled with Gasterosteiformes

删除的内容: at about

删除的内容: 70.3-87.3

删除的内容: whole

删除的内容: accounting for 96.3% of the estimated genome size

删除的内容: 808.9

删除的内容: also

删除的内容: of all species

删除的内容: , which reached a remarkable high level of continuity with contig N50 of 2.1 Mb and scaffold N50 of 33.5 Mb

删除的内容: The contig N50 was remarkably longer than those of most fish genome assemblies, and was comparable with those of recently reported model fish species.

删除的内容: 24

删除的内容: Twenty-four scaffolds corresponding to the twenty-four chromosomes were firstly assembled to a final size of 768.8 Mb using 1372 contigs based on the Hi-C assembly.

删除的内容: the taxonomy of Notothenioidei should be elevated to the order level and

删除的内容: at about

删除的内容: 70.3-87.3

The chromosome-level genome assembly together with gene annotation data generated for the female fish in this work will provide a valuable resource for further research on sex-determining mechanisms, especially for obtaining an accurate assembly of the Y chromosome in male fish. These results will also accelerate genome-wide association studies in resistant breeding systems.

删除的内容: The

删除的内容: ,

删除的内容: ed

删除的内容: and

删除的内容: for

删除的内容: ing

删除的内容: the

删除的内容: on

Ethics Statement

This research was approved by the Animal Care and Use committee of Chinese Academic Science. All participates consent the study under the 'Ethics, consent and permissions' heading. All participants consent to publish the work under the 'Consent to publish' heading.

Availability of supporting data

Supporting data and materials are available in the GigaScience GigaDB database, with the raw sequences deposited in the SRA under the accession number SRP158313.

Competing interests

The authors declare that they have no competing interests.

Funding

This study was supported by a grant from the National Natural Science Foundation of China (No. 41506170, No. 31672672, and No. 31872195), Shandong Province Key Research and Invention Program (2017GHY15102, 2017GHY15106), Qingdao Source Innovation Program (17-1-1-57-jch), STS (2017, 2018), Marine Fishery Institute of Zhejiang Province, Key Laboratory of Mariculture and Enhancement of Zhejiang Province (2016KF002). Qingdao National Laboratory for Marine Science and Technology (2015ASKJ02, 2015ASKJ02-03-03), China Agriculture Research System (CARS-47), STS project (KFZD-SW-106, ZSSD-019).

Author Contributions

YSX conceived the project. ZZX, DYM collected the samples and extracted the genomic DNA. YSX, JL and JL performed the genome assembly and data analysis. YSX, ZZX, JL, DYM and JL wrote the paper.

Reference

1. Schembri, P.J. *et al*. Occurrence of barred kinfejaw, *Oplegnathuf fasciatus* (Actinopterygii: Perciformes: Oplegnathidae), in Malta (Central Mediterranean) with a discussion on possible modes of entry. *Acta Ichthyol Piscat* 40,101-104 (2010).

2. Mundy, B.C. Checklist of the fishes of the Hawaiian Archipelago. *Bishop Mus Bull Zool* 6, 1-704 (2005).

3. An, H.S. & Hong, S.W. Genetic diversity of rock bream *Oplegnathus fasciatus* in Southern Korea. *Genes Genom* 30, 451-459 (2008).

4. Xiao, Y.S. *et al.* Pronounced population genetic differentiation in the rock bream *Oplegnathus fasciatus* inferred from mitochondrial DNA sequences. *Mitochondrial DNA A* 27, 2045-2052 (2016).

5. Park, H.S. *et al*. Population Genetic Structure of Rock Bream (*Oplegnathus fasciatus* Temminck & Schlegel, 1884) Revealed by mtDNA COI Sequence in Korea and China. Ocean Sci J 53, 261-274 (2018).

6. Xu, D.D. *et al*. Chromosomal mapping of microsatellite repeats in the rock bream fish *Oplegnathus fasciatus*, with emphasis of their distribution in the neo-Y chromosome. *Mol Cytogenet* 6, 12 (2013).
7. Xue, R. *et al.* Karyotype and Ag-Nors In Male And Female Of *Oplegnathus Punctatus*. *Oceanol Limnol Sin* 47, 626-632 (2016).
8. Xiao, Z.Z. Study on population genetics and culture biology of *Oplegnathus fasciatus*. Doctor thesis p 162-176 (2015).

9. Zhang, B.C. *et al*. Rock bream (*Oplegnathus fasciatus*) viperin is a virus-responsive protein that modulates innate immunity and promotes resistance against megalocytivirus infection. *Dev Comp Immunol* 45, 35-42 (2014).

10. L, H. *et al*. Characterization of an Iridovirus Detected in Rock Bream (Oplegnathus fasciatus ;Temminck and Schlegel). *Chin J Virol* 27, 158-164 (2011).

11. Koren, S. *et al.* Canu: scalable and accurate long-read assembly via adaptive k-mer weighting and repeat separation. *Genome Res* 27, 722 (2017).

12. Marcais, G. & Kingsford, C. A fast, lock-free approach for efficient parallel counting of occurrences of k-mers. *Bioinformatics* 27, 764–70 (2011).

13. Kajitani, R. *et al.* Efficient de novo assembly of highly heterozygous genomes from whole-genome shotgun short reads. *Genome Res* 24, 1384-1395 (2014).

14.

已下移 **[1]:** Sandborn, A.L. *et al*. Chromatin extrusion explains key features of loop and domain formation in wild-type and engineered genomes. *Proc Natl Acad Sci USA* 112, E6456 (2015). .

15. Pryszcz, L. P. & Gabaldón, T. Redundans: an assembly pipeline for highly heterozygous genomes. *Nucleic Acids Res* 44, e113-e113 (2016).

16. Simão, F. A. *et al*. BUSCO: assessing genome assembly and annotation completeness with single-copy orthologs. *Bioinformatics* 31, 3210 (2015).

17. Li, H. & Durbin, R. Fast and accurate short read alignment with Burrows–Wheeler transform. *Bioinformatics* 25, 1754-1760 (2009).

18. Benson, G. Tandem repeats finder: a program to analyze DNA sequences. *Nucleic Acids Res* 27, 573–80 (1999).

19. Tarailo-Graovac, M. & Chen, N. Using RepeatMasker to identify repetitive elements in genomic sequences. In: Editoral board, Baxevanis Andreas D et al. (eds.), Current Protocols in Bioinformatics, Chapter 4:Unit 4 10 (2009).

20. Jurka, J. *et al.* Repbase Update, a database of eukaryotic repetitive elements. Cytogenetic Genome Res 110, 462–67 (2005).

21. Stanke, M. *et al*. AUGUSTUS: a web server for gene finding in eukaryotes. *Nucleic Acids Res* 32(Web Server issue), W309-12 (2004).

22. Cai, Y. *et al.* Computational systems biology methods in molecular biology, chemistry biology, molecular biomedicine, and biopharmacy. *BioMed Res Int* 2014, 746814 (2014).

23. Flicek, P. *et al*. Ensembl 2014. *Nucleic Acids Res* 42, D749-D755 (2014).

24. Gertz, E. M. *et al.* Composition-based statistics and translated nucleotide searches: Improving the TBLASTN module of BLAST. *MBC Biology* 4, 41 (2006).

25. Birney, E. et al. GeneWise and Genomewise. *Genome Res* 14, 988-995 (2004).

26. Trapnell, C. *et al.* TopHat: discovering splice junctions with RNA-Seq. *Bioinformatics* 25, 105-1111 (2009).

27. Ghosh, S. & Chan, C. K. K. Analysis of RNA-Seq data using TopHat and Cufflinks. *Methods Mole Biol* 1374, 339 (2016).

28. Campbell, M. S. *et al*. Genome Annotation and Curation Using MAKER and MAKER-P. *Current Protocols in Bioinformatics* 48, 4.11.11 (2014).

29. Lobo, I. Basic Local Alignment Search Tool (BLAST). *J Mol Biol* 215, 403-410 (2008).

30. Harris, M. A. *et al.* The Gene Ontology (GO) database and informatics resource. *Nucleic Acids Res* (2004).

31. Ogata, H. *et al.* KEGG: Kyoto Encyclopedia of Genes and Genomes. *Nucleic Acids Res* 27, 29-34 (2000).

32. Conesa, A. *et al.* Blast2GO: a universal tool for annotation, visualization and analysis in functional genomics research. *Bioinformatics* 21, 3674 (2005).

33. Lowe, T. M. & Eddy, S. R. tRNAscan-SE: a program for improved detection of transfer RNA genes in genomic sequence. *Nucleic Acids Res* 25, 955-964 (1997).

34. Griffithsjones, S. *et al.* Rfam: an RNA family database. *Nucleic Acids Res* 31, 439 (2003).

35. Lieberman-Aiden, E. *et al*. Comprehensive mapping of long-range interactions reveals folding principles of the human genome. *Science* 326, 289-293 (2009).

36. Rao, S.S.P. *et al.* A 3D map of the human genome at kilobase resolution reveals principles of chromatin looping. *Cell* 159, 1665-1680 (2014).

37. Sandborn, A.L. *et al.* Chromatin extrusion explains key features of loop and domain formation in wild-type and engineered genomes. *Proc Natl Acad Sci USA* 112, E6456 (2015).

已移动(插入) [1]

38. Flot, J.F. *et al*. Contact genomics: scaffolding and phasing (meta) genomes using chromosome be 3D physical signatures. *FEBs Letters* 589, 2966-2974 (2015).

39. Burton, J.N. *et al*. Chromosome-scale scaffolding of de novo genome assemblies based on chromatin interactions. *Nat Biotechnol* 31, 1119-1125 (2013).

40. Lobo, I. Basic Local Alignment Search Tool (BLAST). J Mol Biol 215, 403-410 (2008).

41. Thompson, J. D. *et al.* Multiple sequence alignment using ClustalW and ClustalX. *Curr Protoc Bioinformatics* 2.3. 1-2.3. 22 (2002).

42. Guindon, S. *et al.* PhyML: Fast and Accurate Phylogeny Reconstruction by Maximum Likelihood. *Infect Genet Evol* 9, 384-385 (2009).

43. Yang, Z. & Rannala, B. Bayesian estimation of species divergence times under a molecular clock using multiple fossil calibrations with soft bounds. *Mol Biol Evol* 23, 212-226 (2006).

44. Hedges, S. B. *et al.* Tree of life reveals clock-like speciation and diversification. *Mol Biol Evol* 32, 835-845 (2015).

45. Ricardo, B. R. *et al.* Phylogenetic classification of bony fishes. BMC Evol Biol 17, 162 (2017).

带格式的: 字体: 倾斜

Click here to access/download;Table;2new-Tables.docx

Table 1 Summary of *Oplegnathus fasciatus* genome assembly and annotation

Genome assembly	values
Contig N50 size (Mb)	2.1
Contig number	1,692
Scaffold N50 size (Mb)	33.5
Scaffold N50 number	24
Total length (Mb)	778.7
Genome coverage (X)	314.6
Contig number (≥ 1 Mb)	219
Length of contig (≥ 1 Mb) (bp)	565,184,128
The longest contig (bp)	8,891,851
The longest scaffold (bp)	38,619,456
Genome annotation	
Protein-coding gene number	24,003
Mean transcript length (kb)	16.1
Mean exons per gene	10.1
Mean exon length (bp)	217.7
Mean intron length (bp)	1527.4

Table 2 Hi-C libraries for chromosome-scale assembly of *Oplegnathus fasciatus*

Chromosome	Number of contigs	Length of contigs	Length of chromosome
Chr1	36	19,852,463	19,869,963
Chr2	51	34,905,999	34,930,999
Chr3	43	33,654,321	33,675,321
Chr4	74	35,290,762	35,327,262
Chr5	54	38,592,956	38,619,456
Chr6	72	38,156,734	38,192,234
Chr7	60	35,029,969	35,059,469
Chr8	64	37,546,719	37,578,219
Chr9	45	31,457,603	31,479,603
Chr10	52	35,302,682	35,328,182
Chr11	80	31,971,344	32,010,844
Chr12	46	30,287,574	30,310,074
Chr13	52	33,665,353	33,690,853

Chr14	101	31,190,130	31,240,130
Chr15	48	30,038,946	30,062,446
Chr16	59	28,825,591	28,854,591
Chr17	33	28,220,078	28,236,078
Chr18	50	26,754,155	26,778,655
Chr19	52	34,380,882	34,406,382
Chr20	52	25,675,509	25,701,009
Chr21	64	31,397,692	31,429,192
Chr22	63	30,492,179	30,523,179
Chr23	70	33,514,462	33,548,962
Chr24	51	31,930,140	31,955,140
Unanchored information	384	10,596,846	-
Total	1,372	768,134,243	768,808,243

Table 3 Genome assembly of *Oplegnathus fasciatus* based on chromosome-length scaffolds

	Draft scaffolds	Chromosome-length scaffolds based on Hi-C
Length of genome (bp)	778,731,089	768,808,243
Number of contigs	1,692	1,372
Contigs N50 (bp)	2,149,025	2,130,780
Number of scaffold	/	24
Scaffold N50 (bp)	/	33,548,962
Number of contigs (≥ 100 kb)	693	708
Total length of contigs (≥ 100 kb)	735,235,962	732,827,446
Mapping rate of contigs (≥ 100 kb) (%)	/	99.67

Table 4 The detailed classification of repeat sequences of *Oplegnathus fasciatus*

Type	Repbase TEs		TE proteins		De novo		Combined TEs	
	Length (bp)	% in genome	Length (bp)	% in genome	Length (bp)	% in genome	Length (bp)	% in genome
DNA	39,147,527	5.03	5,390,266	0.69	93,089,344	11.95	124,417,402	15.98
LINE	23,983,322	3.08	16,460,762	2.11	57,167,551	7.34	85,761,250	11.01
SINE	875,585	0.11	0	0.00	914,559	0.12	1,747,250	0.22
LTR	10,163,601	1.31	5,770,483	0.74	31,126,639	4.00	42,465,968	5.45
Satellite	2,028,992	0.26	0	0.00	2,613,480	0.34	4,361,048	0.56
Simple_repeat	1,556,026	0.20	0	0.00	5,179,965	0.67	6,386,303	0.82
Other	6,545	0.00	0	0.00	0	0.00	6,545	0.00
Unknown	331,430	0.04	0	0.00	20,636,768	2.65	20,967,052	2.69
Total	73,544,786	9.44	27,613,880	3.55	183,954,095	23.62	250,611,845	32.18

Figure
Click here to access/download;Figure;3new-Figure Legends.docx

Figure Legends

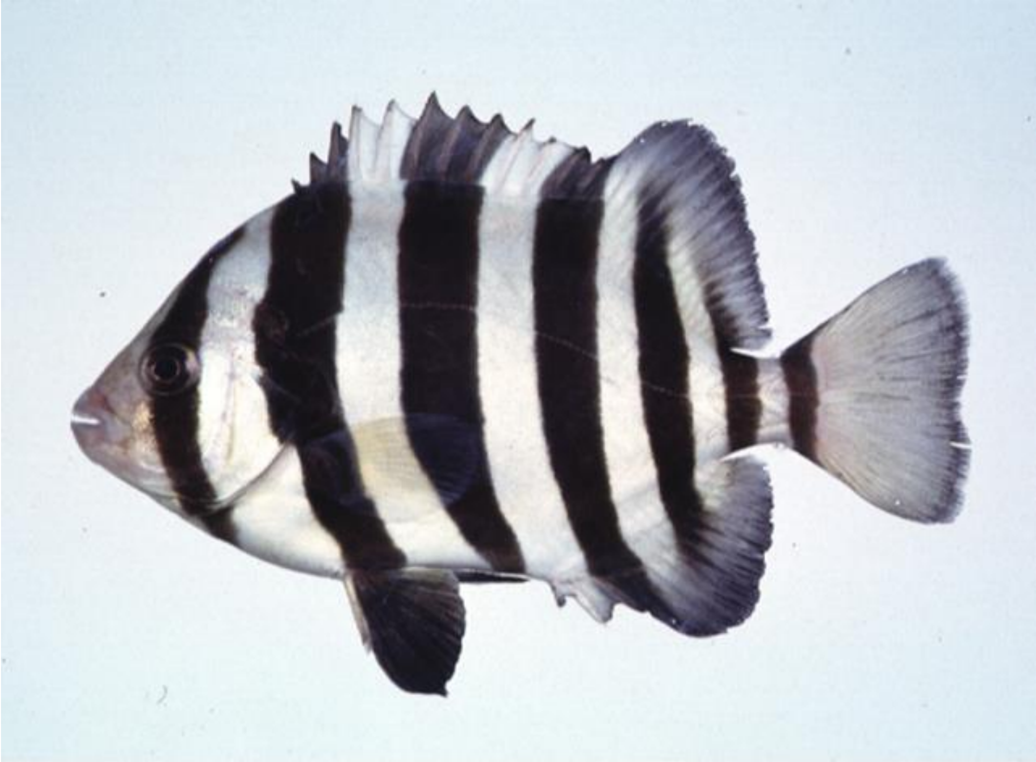

Fig. 1 A representative individual of *O. fasciatus*

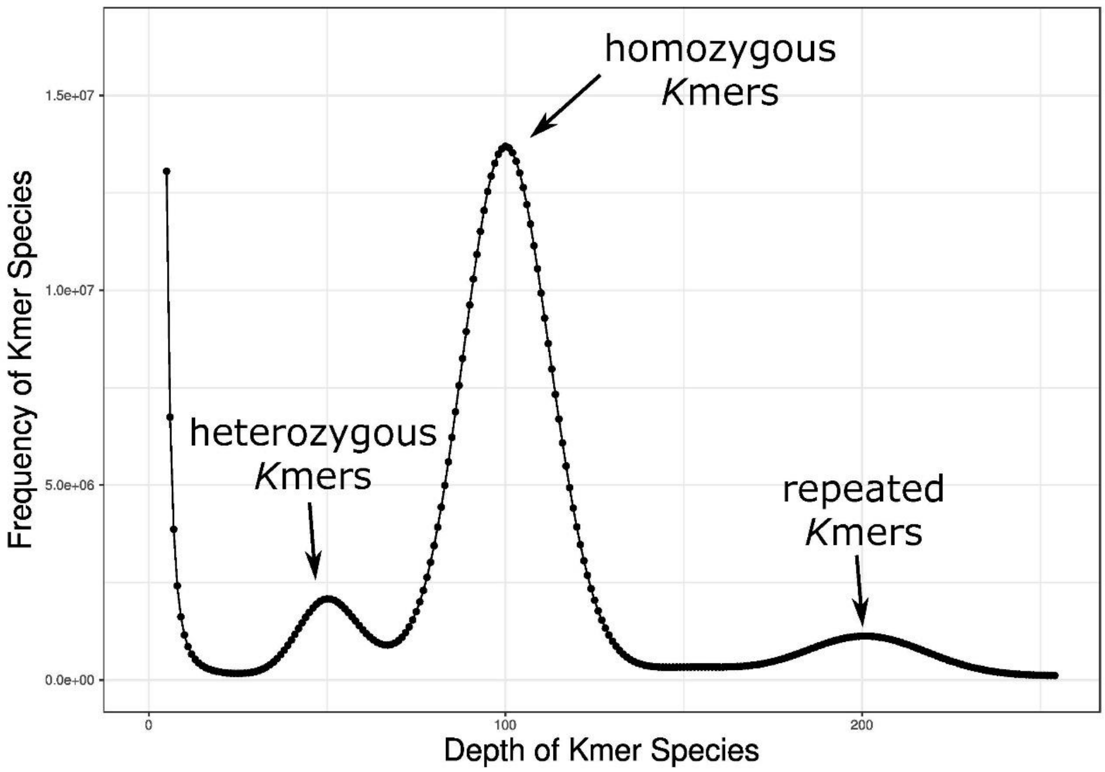

Fig. 2 *k*-mer distribution of the *O. fasciatus* genome

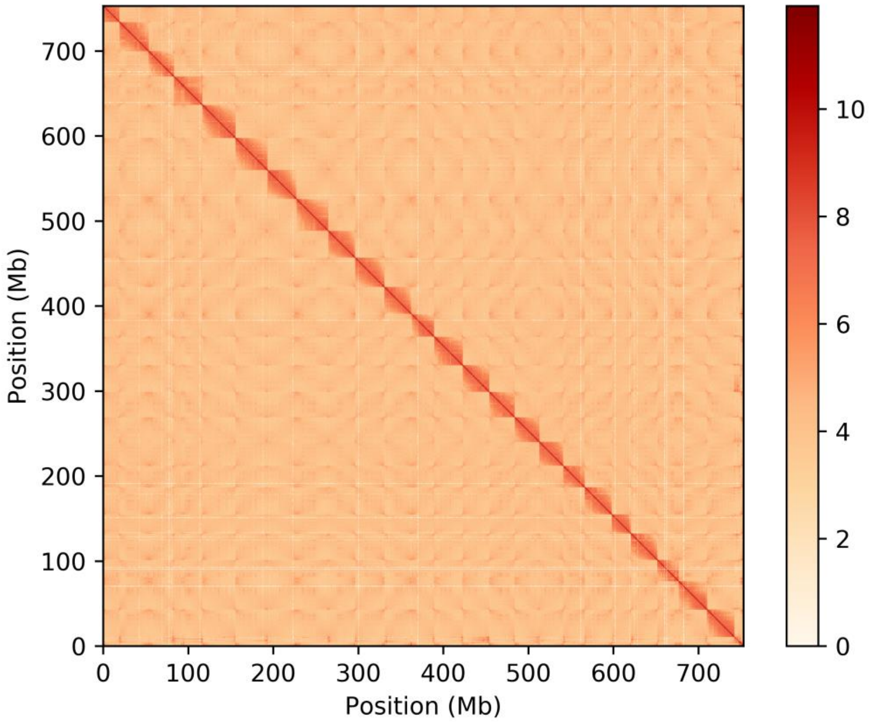

Fig. 3 Hi-C interaction heatmap for *O. fasciatus* reference genome, showing interactions between the 24 chromosomes

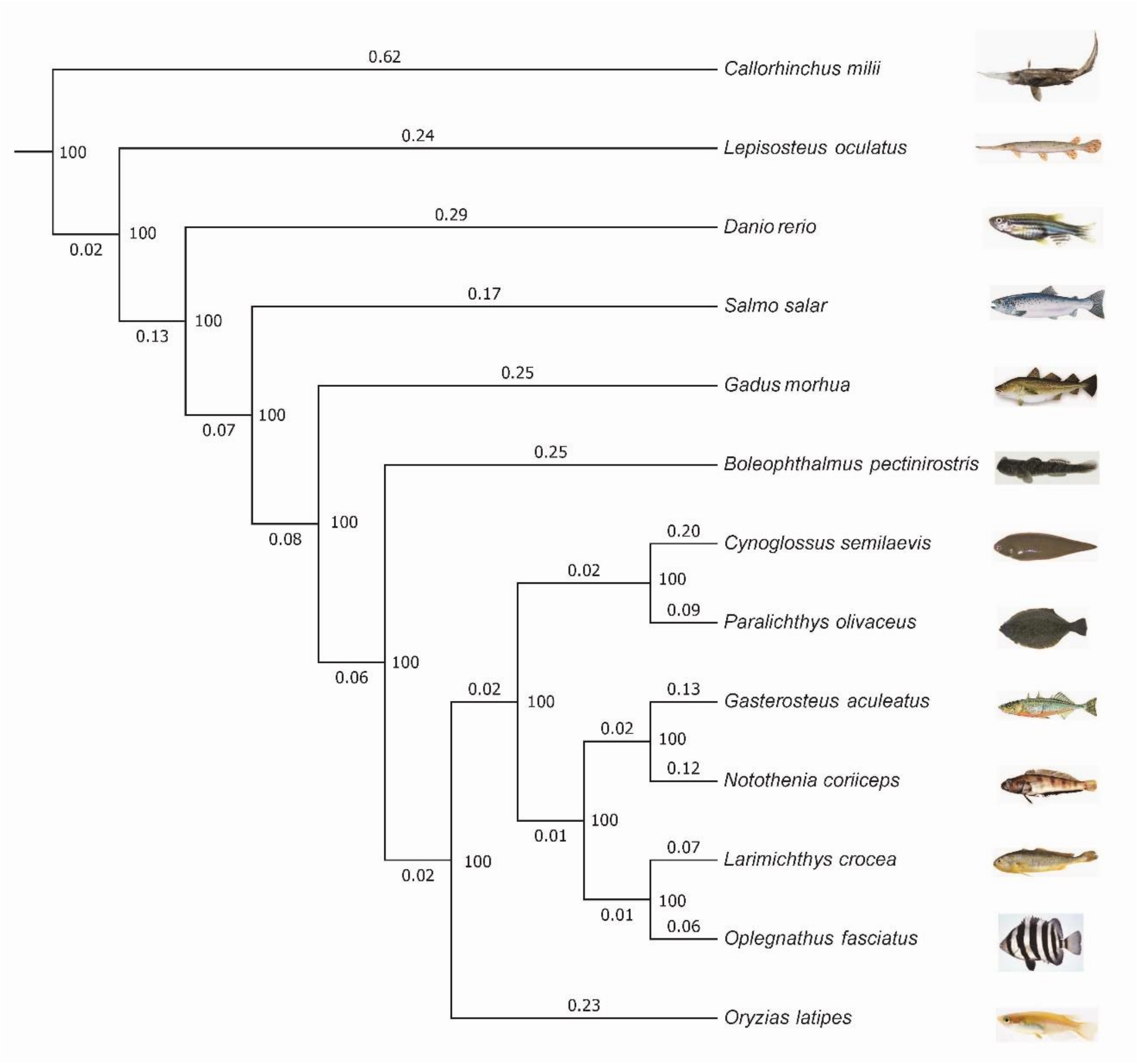

Fig. 4 The phylogenetic relationships of *O. fasciatus* with other fishes. The bootstrap values (larger than 1) calculated from 1000 bootstrap replicates and the branch lengths (samller than 1) were labelled at and below/above each branch, respectively

Click here to access/download
Supplementary Material
4new-supplementary materials.docx

Click here to access/download
Supplementary Material
New-Response to Reviewers with point-by-point.doc
