# Supplementary material for: Genome sequence of the barred knifejaw Oplegnathus fasciatus (Temminck & Schlegel, 1844): the first chromosome-level draft genome in the family Oplegnathidae
Source: Gigascience. 2019 Feb 1;8(3):giz013. doi: 10.1093/gigascience/giz013 (PMC6423371; doi:10.1093/gigascience/giz013)
Supplement: Supplemental Files [file giz013_supplemental_files.zip › 4new-new-supplementary materials.docx]

S Fig. 1 The GC content of *O. fasciatus* base on the Illumina platform for genome size survey


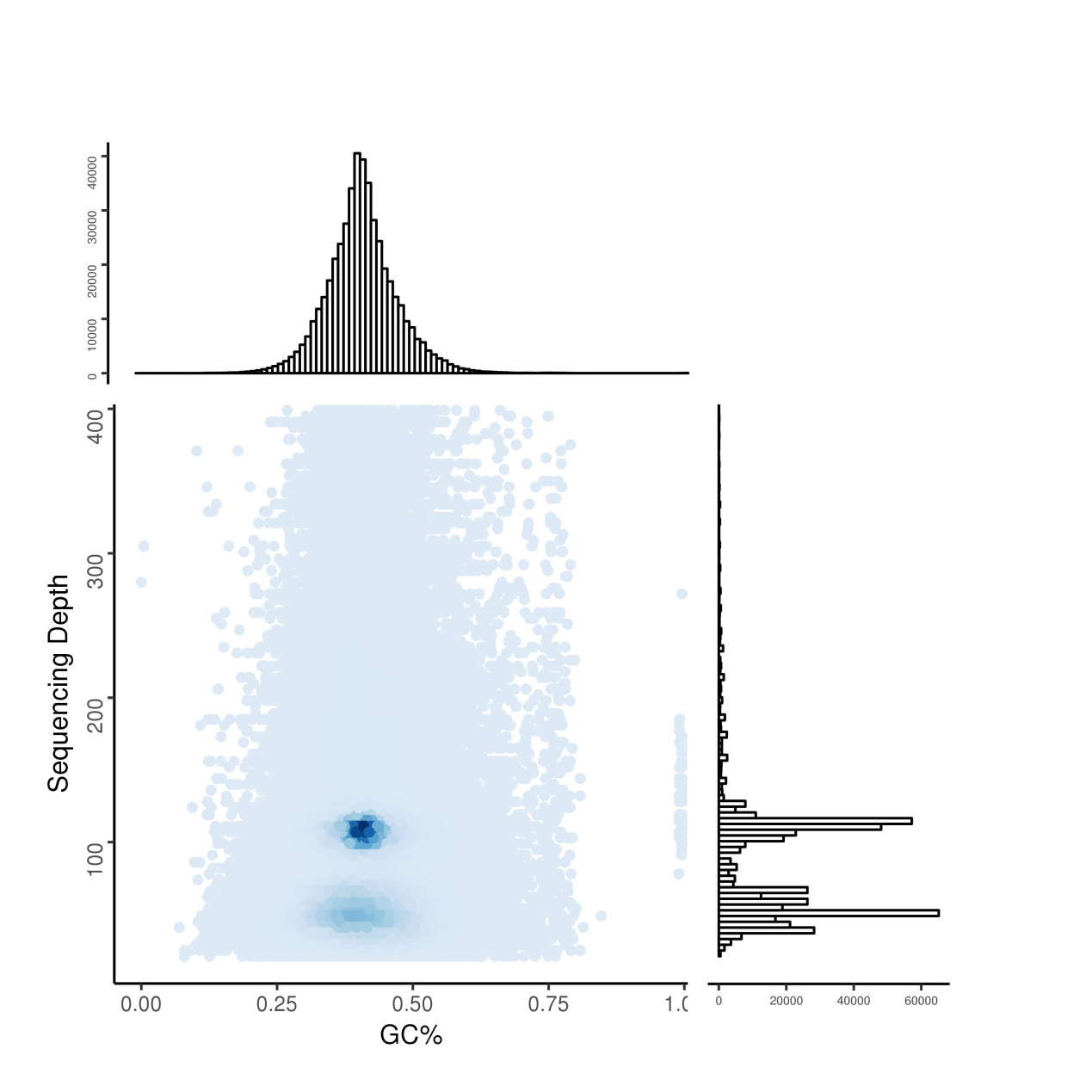


S Fig. 2 The content of interspersed repeats of *O. fasciatus* genome assembly. (a) Repbase library (b) *de novo* library


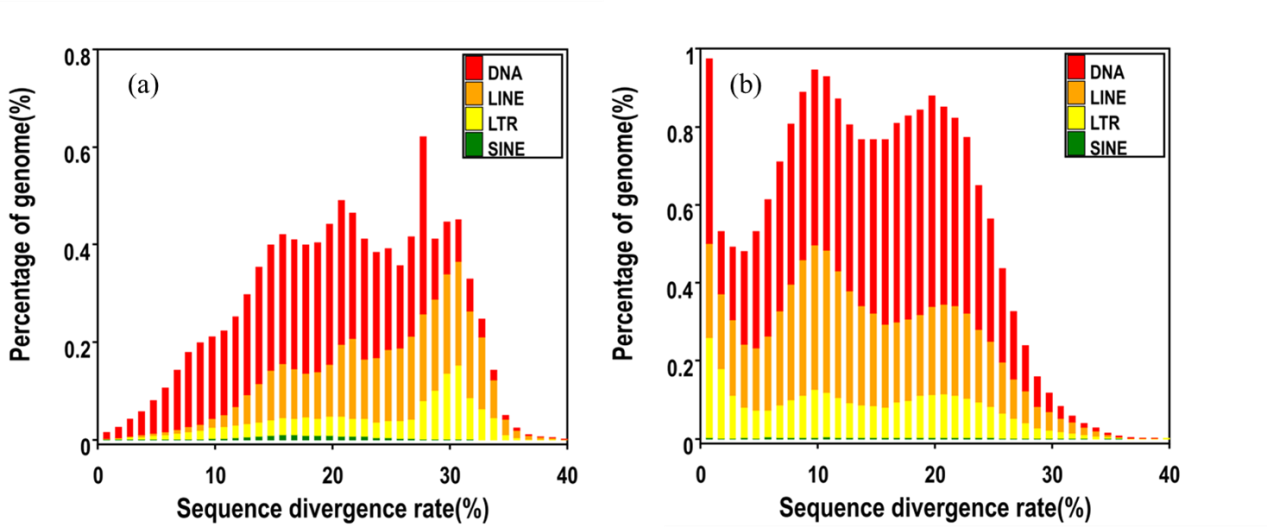


S Fig. 3 The gene number, gene length distribution, CDS length distribution, exon length distribution and intron length distribution were all comparable with those in other teleost fish species.


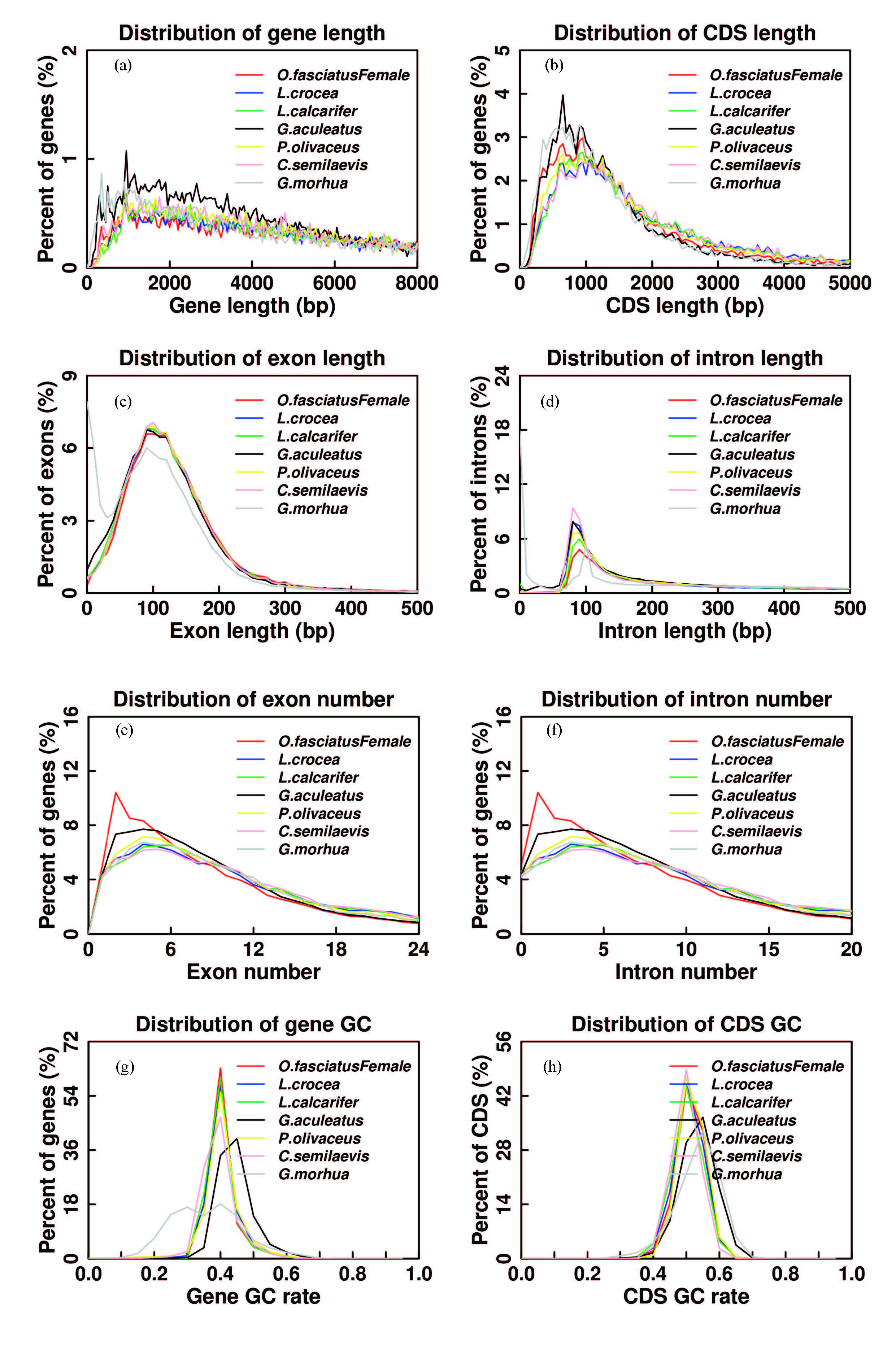


S Fig. 4 Comparing genome assemblies between *O. fasciatus* and other fish species.


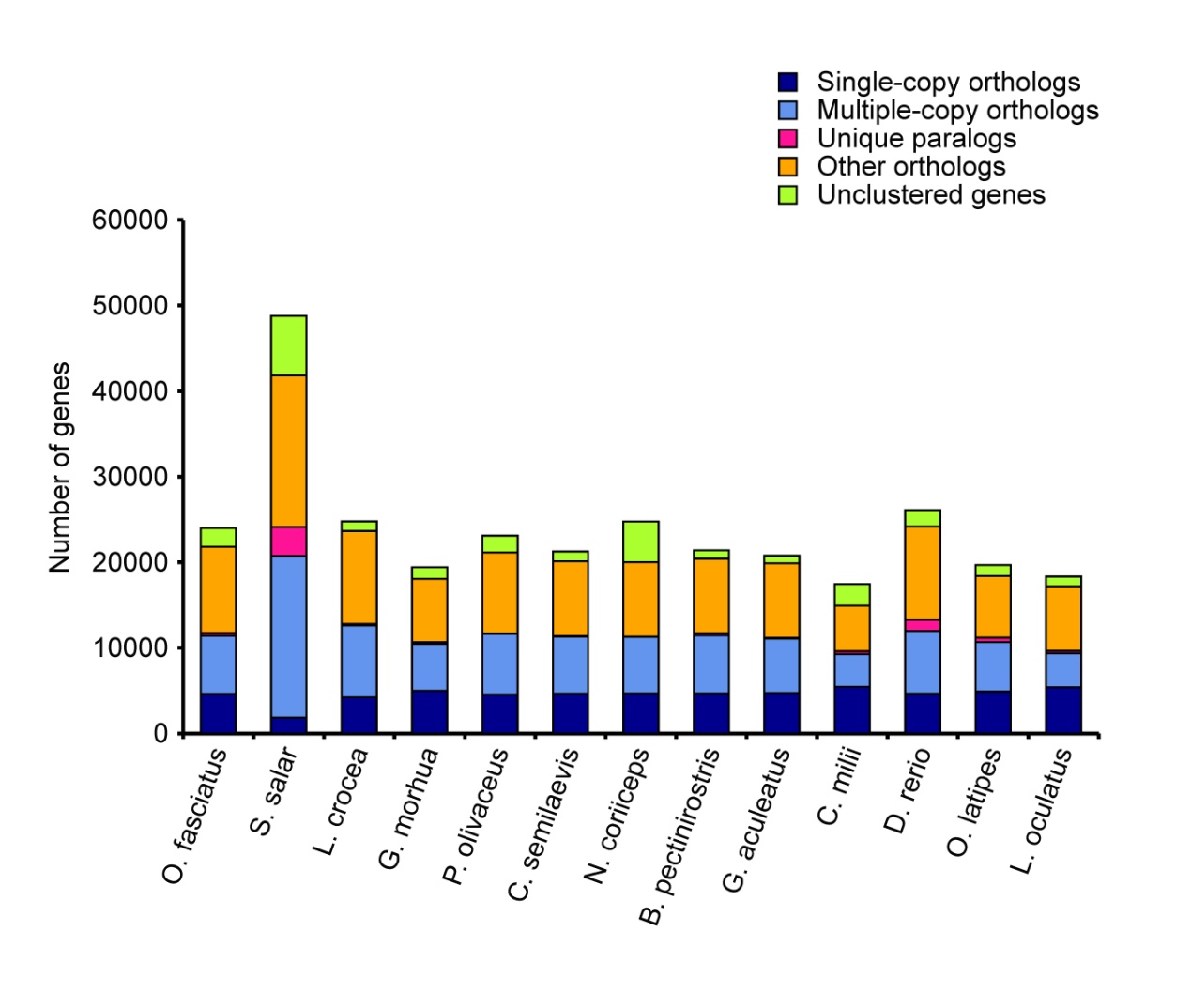


S Fig. 5 Orthologous gene families across four fish genomes (*Oplegnathus fasciatus*, *Larimichthys crocea*, *Gadus morhua* and *Salmo salar*)


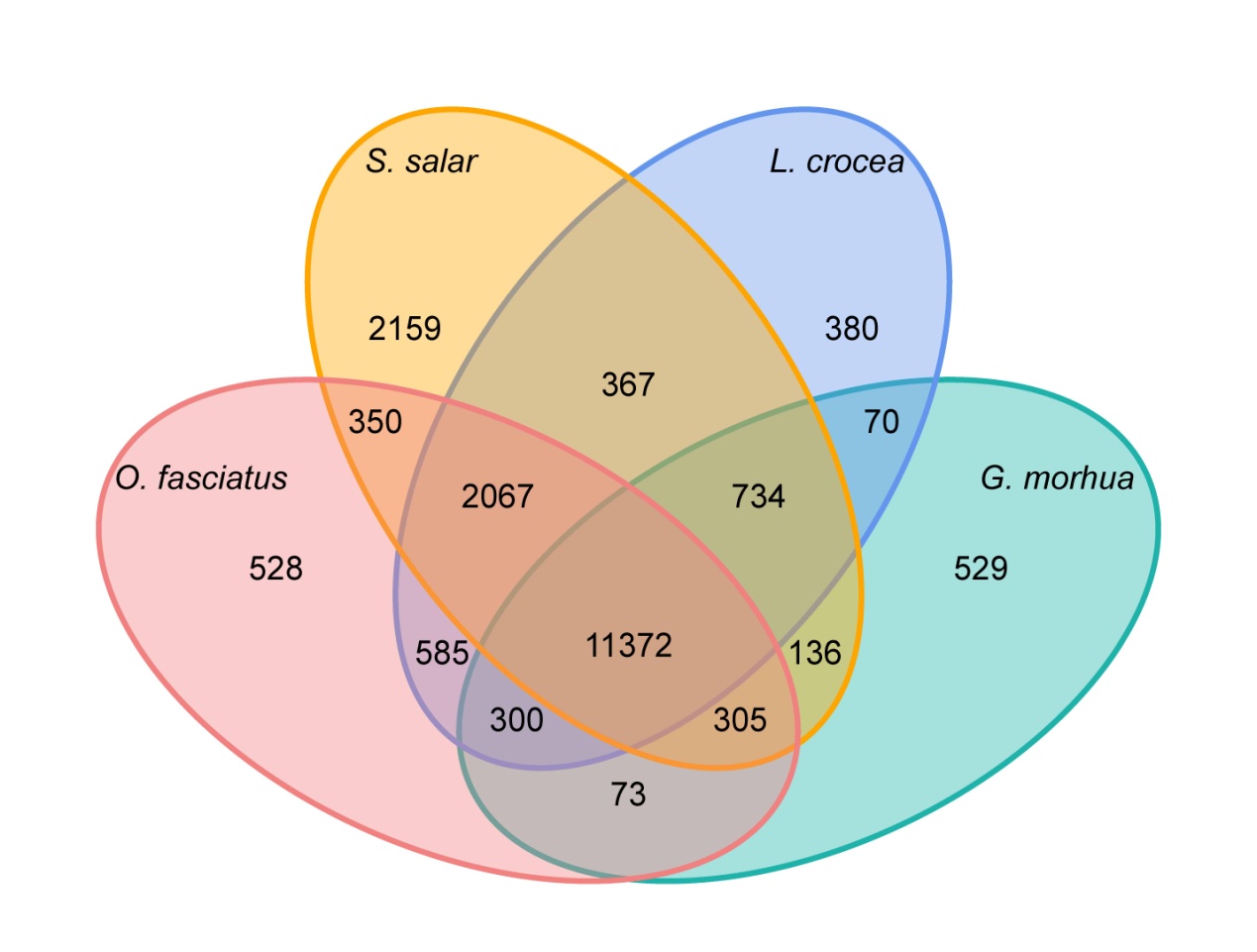


S Table 1 Summary of sequence data from *O. fasciatus*

| Type | Method | Library size (bp) | Data size (Gb) | Read N50 (bp) |
| --- | --- | --- | --- | --- |
| DNA | HiSeq 2000 | 300~350 | 90.7 | 150 |
| DNA | PacBio Sequel | 22 000 | 62.8 | 14,555 |
| DNA (Hi-C) | HiSeq 2000 | 300~350 | 91.5 | 150 |
| RNA | HiSeq 2000 | 150 | 42.2 | 150 |

S Table 2 Genome assembly statistics for *O. fasciatus*

| Method | Type | Genome size (Mb) | Longest sequence (Mb) | Sequences number | Sequence N50 (Mb) |
| --- | --- | --- | --- | --- | --- |
| Platanus | Contig | 875.4 | 0.109 | 1,053,100 | 0.0072 |
|  | Scaffold | 744.5 | 0.939 | 21,737 | 0.0841 |
| Canu | Contig | 875.9 | 8.881 | 4,186 | 1.7836 |
| Redundans | Contig | 778.0 | 8.881 | 1,692 | 2.1467 |
| Smrtlink | Contig | 778.8 | 8.893 | 1,692 | 2.1490 |
| Pilon | Contig | 778.7 | 8.891 | 1,692 | 2.1490 |
| Final | Contig | 778.7 | 8.891 | 1,692 | 2.149 |

S Table 3 Comparing genome assemblies between *O. fasciatus* and other fish species

Some data were cited from the reference (Gaorui Gong, Cheng Dan, Shijun Xiao, Wenjie Guo, Peipei Huang, Yang Xiong, Junjie Wu, Yan He, Jicheng Zhang, Xiaohui Li, Nansheng Chen, Jian-Fang Gui, Jie Mei; Chromosomal-level assembly of yellow catfish genome using third-generation DNA sequencing and Hi-C analysis, GigaScience, Volume 7, Issue 11, 1 November 2018, giy120, doi.org/10.1093)

| Species | Estimated Genome Size (Mb) | Contig N50 (Mb) | Scaffold N50 (Mb) |
| --- | --- | --- | --- |
| *Oplegnathus fasciatus* | 768 | 2.13 | 33.55 |
| *Larimichthys crocea* | 679 | 0.06 | 1.03 |
| *Ictalurus punctatus* | 845 | 0.05 | 7.20 |
| *Paralichthys olivaceus* | 546 | 0.03 | 3.90 |
| *Hippocampus comes* | 501 | 0.04 | 1.80 |
| *Gadus morhua* | 643 | 0.12 | 1.15 |
| *Channa argus* | 615 | 0.08 | 4.50 |
| *Anguilla rostrata* | 1,410 | 0.01 | 0.086 |
| *Anguilla anguilla* | 860 | 0.001 | 0.077 |
| *Lates calcarifer* | 700 | 1.06 | 25.80 |
| *Clupea harengus* | 850 | 0.02 | 1.84 |
| *Scophthalmus maximus* | 568 | 0.03 | 4.30 |
| *Miichthys miiuy* | 636 | 0.07 | 1.15 |
| ***Lepisosteus oculatus*** | **945** | **0.07** | **6.90** |
| ***Sillago sinica*** | **534** | **2.60** | **-** |
| ***Lates calcarifer*** | **586** | **1.07** | **25.85** |
| ***Oreochromis niloticus*** | **868** | **3.30** | **37.00** |

S Table 4 Aligment of clean reads for Hi-C data and Hi-C libraries for chromosome-scale assembly

| Data | | 628,779,292 | |
| --- | --- | --- | --- |
| Paired | | 593,676,716 (94.42%) | |
| With mate mapped to a different contig (or scaffold) | | 240,530,625 (40.52%) | |
| With mate mapped to a different contig (or scaffold) (mapQ>≥5) | | 205,091,925 (34.55%) | |
| Chromosome | Number of contigs | Length of contigs | Length of chromosome |
| Chr1 | 36 | 19,852,463 | 19,869,963 |
| Chr2 | 51 | 34,905,999 | 34,930,999 |
| Chr3 | 43 | 33,654,321 | 33,675,321 |
| Chr4 | 74 | 35,290,762 | 35,327,262 |
| Chr5 | 54 | 38,592,956 | 38,619,456 |
| Chr6 | 72 | 38,156,734 | 38,192,234 |
| Chr7 | 60 | 35,029,969 | 35,059,469 |
| Chr8 | 64 | 37,546,719 | 37,578,219 |
| Chr9 | 45 | 31,457,603 | 31,479,603 |
| Chr10 | 52 | 35,302,682 | 35,328,182 |
| Chr11 | 80 | 31,971,344 | 32,010,844 |
| Chr12 | 46 | 30,287,574 | 30,310,074 |
| Chr13 | 52 | 33,665,353 | 33,690,853 |
| Chr14 | 101 | 31,190,130 | 31,240,130 |
| Chr15 | 48 | 30,038,946 | 30,062,446 |
| Chr16 | 59 | 28,825,591 | 28,854,591 |
| Chr17 | 33 | 28,220,078 | 28,236,078 |
| Chr18 | 50 | 26,754,155 | 26,778,655 |
| Chr19 | 52 | 34,380,882 | 34,406,382 |
| Chr20 | 52 | 25,675,509 | 25,701,009 |
| Chr21 | 64 | 31,397,692 | 31,429,192 |
| Chr22 | 63 | 30,492,179 | 30,523,179 |
| Chr23 | 70 | 33,514,462 | 33,548,962 |
| Chr24 | 51 | 31,930,140 | 31,955,140 |
| Unanchored information | 384 | 10,596,846 | - |
| Total | 1,372 | 768,134,243 | 768,808,243 |

S Table 5 Genome quality of *Oplegnathus fasciatus* based on the BUSCO assessment

| Type | Assembly | | Annotation | |
| --- | --- | --- | --- | --- |
|  | Proteins | Percentage (%) | Proteins | Percentage (%) |
| Complete BUSCOs | 4,430 | 96.6 | 4,391 | 95.8 |
| Complete and single-copy BUSCOs | 4,259 | 92.9 | 4,187 | 91.3 |
| Complete and duplicated BUSCOs | 171 | 3.7 | 204 | 4.5 |
| Fragmented BUSCOs | 69 | 1.5 | 108 | 2.4 |
| Missing BUSCOs | 85 | 1.9 | 85 | 1.8 |
| Total BUSCOs groups searched | 4,584 | 100.0 | 4,584 | 100.0 |

S Table 6 The estimation of the completeness for *O. fasciatus* genome assembly based on CLR (Continuous Long Reads) subreads mapping

| Mapping rate (%) | Average sequencing depth | Coverage (%) | Coverage (≥5X, %) | Coverage (≥10X, %) | Coverage(≥20X, %) |
| --- | --- | --- | --- | --- | --- |
| 90.17 | 80.62 | 99.92 | 99.68 | 98.31 | 98.45 |

S Table 7 The estimation of the accuracy for *O. fasciatus* genome assembly based on SNP calling

| SNP | Number | Percentage of SNP (%) | Percentage of genome (%) |
| --- | --- | --- | --- |
| All SNP | 1,562,433 | 100.00 | 0.20 |
| Heterozygosis SNP | 1,558,878 | 99.77 | 0.20 |
| Homology SNP | 3,555 | 0.23 | 0.00 |
| All indel | 664,590 | 100.00 | 0.09 |
| Heterozygosis indel | 649,846 | 97.78 | 0.08 |
| Homology indel | 14,744 | 2.22 | 0.00 |

S Table 8 Transcriptome data from RNA-seq for *O. fasciatus*

| Sample | Before filter | | | After filter | | | Gene No. | Read N50 | Max length | Average length |
| --- | --- | --- | --- | --- | --- | --- | --- | --- | --- | --- |
|  | Before filter data (bp) | Q20 (%) | Q30 (%) | After filter data (bp) | Q20 (%) | Q30 (%) | 62,467 | 2,450 bp | 59,651 bp | 1,214 bp |
| CK-1 | 4 193 837 760 | 96.13 | 91.49 | 3,946,007,277 | 98.13 | 94.56 |  |  |  |  |
| CK-2 | 4 542 887 548 | 96.23 | 91.66 | 4,287,840,962 | 98.16 | 91.61 |  |  |  |  |
| CK-3 | 3 707 145 734 | 97.34 | 93.32 | 3,557,038,640 | 98.45 | 95.12 |  |  |  |  |
| VC-1 | 3 855 448 572 | 97.42 | 93.49 | 3,703,645,388 | 98.49 | 95.23 |  |  |  |  |
| VC-2 | 4 565 074 582 | 97.40 | 93.44 | 4,386,344,462 | 98.47 | 95.19 |  |  |  |  |
| VC-3 | 4 180 130 282 | 97.34 | 93.30 | 4,012,959,725 | 98.45 | 95.10 |  |  |  |  |
| TP-1 | 3 379 514 994 | 97.35 | 93.32 | 3,242,985,938 | 98.45 | 95.13 |  |  |  |  |
| TP-2 | 3 281 341 740 | 97.43 | 93.47 | 3,157,433,918 | 98.47 | 95.16 |  |  |  |  |
| TP-3 | 3 647 614 890 | 97.45 | 93.55 | 3,509,897,398 | 98.50 | 95.25 |  |  |  |  |
| NC-1 | 3 398 536 162 | 97.44 | 93.52 | 3,268,777,185 | 98.49 | 95.22 |  |  |  |  |
| NC-2 | 3 404 172 992 | 97.44 | 93.53 | 3,276,779,782 | 98.48 | 95.24 |  |  |  |  |
| NC-3 | 3 488 720 308 | 97.51 | 93.66 | 3,359,820,359 | 98.52 | 95.31 |  |  |  |  |

S Table 9 Gene annotation of the *O. fasciatus* genome

| Gene set | | Number | Average gene length (bp) | Average CDS length (bp) | Average exon per gene | Average exon length (bp) | Average intron length (bp) |
| --- | --- | --- | --- | --- | --- | --- | --- |
| *De novo* | AUGUSTUS | 28,841 | 13 713.56 | 1 446.83 | 8.34 | 173.38 | 1 670.14 |
|  | Genscan | 33,608 | 16 625.47 | 1 526.63 | 8.66 | 176.19 | 1 969.90 |
| Homolog | *Larimichthys crocea* | 45,678 | 9 344.73 | 1 110.43 | 5.87 | 189.21 | 1 691.21 |
|  | *Lates calcarifer* | 46,847 | 9 465.65 | 1 129.32 | 5.96 | 189.40 | 1 679.82 |
|  | *Gasterosteus aculeatus* | 38,452 | 8 843.19 | 1 060.11 | 6.06 | 174.88 | 1 537.58 |
|  | *Paralichthys olivaceus* | 40,607 | 8 951.38 | 1 090.20 | 5.95 | 183.20 | 1 587.80 |
|  | *Cynoglossus semilaevis* | 39,942 | 9 784.28 | 1 115.84 | 6.09 | 183.26 | 1 703.42 |
|  | *Gadus morhua* | 12,246 | 8 823.65 | 954.00 | 5.96 | 160.11 | 1 587.18 |
| RNAseq | | 18,383 | 15 810.61 | 1 351.12 | 9.04 | 335.5. | 1 589.31 |
| BUSCO | | 4,686 | 13 536.46 | 1 947.96 | 12.64 | 154.05 | 995.17 |
| MAKER | | 23,494 | 17 526.92 | 1 705.37 | 10.35 | 249.41 | 1 598.91 |
| HICESAP | | 24,003 | 16 099.57 | 1 697.17 | 10.10 | 217.71 | 1527.37 |

S Table 10 Functional annotation of the protein-coding genes in *O. fasciatus* genome

| Type | | Number | Percent (%) |
| --- | --- | --- | --- |
| Total | | 24,003 |  |
| Annotated | InterPro | 20,945 | 87.26 |
|  | GO | 15,977 | 66.56 |
|  | KEGG_ALL | 23,185 | 96.59 |
|  | KEGG_KO | 14,838 | 61.82 |
|  | Swissprot | 21,738 | 90.56 |
|  | TrEMBL | 23,227 | 96.77 |
|  | NR | 23,332 | 97.20 |
| Annotated | | 23,364 | 97.34 |
| Unannotated | | 639 | 2.66 |

S Table 11 The annotation of non-coding RNAs of *O. fasciatus* genome

| Type | | Copy | Average length (bp) | Total length (bp) | % of genome |
| --- | --- | --- | --- | --- | --- |
| miRNA | | 602 | 86.25 | 51,921 | 0.007 |
| tRNA | | 1,484 | 75.64 | 112,250 | 0.014 |
| rRNA | rRNA | 207 | 180.04 | 37,269 | 0.005 |
|  | 18S | 9 | 1 657.89 | 14,921 | 0.002 |
|  | 28S | 0 | 0.00 | 0 | 0.000 |
|  | 5.8S | 7 | 156.00 | 1,092 | 0.000 |
|  | 5S | 191 | 111.29 | 21,256 | 0.003 |
|  | 8S | 0 | 0.00 | 0 | 0.000 |
| snRNA | snRNA | 474 | 135.51 | 64,234 | 0.008 |
|  | CD-box | 123 | 108.18 | 13,306 | 0.002 |
|  | HACA-box | 76 | 144.25 | 10,963 | 0.001 |
|  | Splicing | 266 | 143.38 | 38,140 | 0.005 |
|  | scaRNA | 9 | 202.78 | 1,825 | 0.000 |
