# Supplementary material for: Genome sequence of the barred knifejaw Oplegnathus fasciatus (Temminck & Schlegel, 1844): the first chromosome-level draft genome in the family Oplegnathidae
Source: Gigascience. 2019 Feb 1;8(3):giz013. doi: 10.1093/gigascience/giz013 (PMC6423371; doi:10.1093/gigascience/giz013)
Supplement: Supplemental Files [file giz013_supplemental_files.zip › New manuscript-tracked changes.docx]

Genome sequence of the barred knifejaw Oplegnathus fasciatus (Temminck & Schlegel, 1844): the first chromosome-level draft genome in the family Oplegnathidae

Yongshuang Xiao^1,2,3*^ ^†^, Zhizhong Xiao^1,2,3, †^, Daoyuan Ma^1,2,3^, Jing Liu^2,3*^, Jun Li^1,2,3*^

^1^Institute of Oceanology, Chinese Academy of Sciences, 7 Nanhai Road, Qingdao, 266071, China, ^2^ Laboratory for Marine Biology and Biotechnology, Qingdao National Laboratory for Marine Science and Technology, 7 Nanhai Road, Qingdao, 266071, China, ^3^Center for Ocean Mega-Science, Chinese Academy of Sciences, 7 Nanhai Road, Qingdao, 266071, China

∗Correspondence address: Yongshuang Xiao, Mega-Science, Chinese Academy of Sciences, 7 Nanhai Road, Qingdao, 266071, China; Tel: +86-053282896729; E-mail: dahaishuang1982@163.com; Jing Liu, Institute of Oceanology, Chinese Academy of Sciences, 7 Nanhai Road, Qingdao, 266071, China; Tel: +86-053282898790; E-mail: jliu@qdio.ac.cn; Jun Li, Institute of Oceanology, Chinese Academy of Sciences, 7 Nanhai Road, Qingdao, 266071, China; Tel: +86-053282898718; E-mail: junli@qdio.ac.cn.

†Contributed equally to this work.

**Abstract**

**Background**

The barred knifejaw (*Oplegnathus fasciatus*), a member of the Oplegnathidae family of the Centrarchiformes, is a commercially important rocky reef fish native to East Asia. *O. fasciatus* has become an important fishery resource for offshore cage aquaculture and fish stocking of marine ranching in China, Japan and Korea. Recently, sexual dimorphism in growth with neo-sex chromosome and widespread biotic diseases in *O. fasciatus* has been received increasing concern. However, adequate genome resources for gaining insight into sex-determining mechanisms and establishing genetically resistant breeding systems for *O. fasciatus* are lacking. Here, we analysed the entire genome of a female *O. fasciatus* fish using long-read sequencing and Hi-C data to generate chromosome-length scaffolds and a highly contiguous genome assembly.

**Findings**

We assembled the *O. fasciatus* genome with a total of 245.0 Gb of raw reads that were generated using both of PacBio Sequel and Illumina HiSeq 2000 platforms. The final draft genome assembly was approximately 778.7 Mb, which reached a high level of continuity with a contig N50 of 2.1 Mb. The genome size was consistent with the estimated genome size (777.5 Mb) based on *k*-mer analysis. We combined Hi-C data with a draft genome assembly to generate chromosome-length scaffolds. Twenty-four scaffolds corresponding to the twenty-four chromosomes were assembled to a final size of 768.8 Mb with a contig N50 of 2.1 Mb and a scaffold N50 of 33.5 Mb using 1,372 contigs. The identified repeat sequences accounted for 33.9% of the entire genome, and 24,003 protein-coding genes with an average of 10.1 exons per gene were annotated using *de novo* methods, with RNA-seq data and homologies to other teleosts. According to phylogenetic analysis using protein-coding genes, *O. fasciatus* is closely related to *Larimichthys crocea*, with *O. fasciatus* diverging from their common ancestor approximately 70.5-88.5 million years ago.

**Conclusions**

We generated a high-quality draft genome with chromosome assembly for *O. fasciatus* using long reads by using the PacBio sequencing technologies, which represents the first chromosome-level reference genome for Oplegnathidae species. Assembly of this genome will provide insight into sex-determining mechanisms and serve as a resource for accelerating genome-assisted improvements in resistant breeding systems.

*Keywords*: *Oplegnathus fasciatus*; chromosome-level genome assembly; Hi-C assembly; sex-determining mechanism

**Data description**

**Introduction of *O. fasciatus***

The Oplegnathidae family belongs to the order Centrarchiformes, including only one genus *Oplegnathus*, which is comprised of seven species (*O. conwayi*, *O. fasciatus*, *O. insignis*, *O. peaolopesi*, *O. punctatus*, *O. robinsoni*, *O. woodwardi*), two of which (*O. fasciatus* and *O. punctatus*) are commercially valuable in East Asia. The barred knifejaw *O. fasciatus* (Temminck & Schlegel, 1844) is one of these two species in the *Oplegnathus*, which is commonly found at the depth of one to ten metres in association with rocky reefs^1, 2^, and distributed across a wide range of shallow waters around Korea, Japan, China and Hawaii^1, 3, 4^ (Fig. 1). *O. fasciatus* has become an important fishery resource for offshore cage aquaculture and fish stocking of marine ranching in China, Japan and Korea^5^. It has been reported that the male of *Oplegnathus* possesses a neo-sex chromosome, possibly a sex chromosome Y. The sex chromosome system for *Oplegnathus* is considered to be X_1_ X_1_ X_2_ X_2_ / X_1_ X_2_ Y based on karyotype analyses^6, 7^. Furthermore, sexual dimorphism in growth has been detected in the *O. fasciatus*, with male fish exhibiting faster growth than females, possibly be due to the sex chromosome system in *Oplegnathus*^8^. *O. fasciatus* is vulnerable to viruses (e.g., Iridovirus) and genetic degradation caused by inbreeding has led to higher susceptibility to diseases^9, 10^. It is vital to develop genomic resources to gain insight into sex-determining mechanisms and to accelerate the genome-assisted improvements in resistant breeding systems.

So far, a genome sequence with the chromosomal assembly of *O. fasciatus* has not been reported. Here, we constructed a high-quality chromosome-level reference genome assembly for *O. fasciatus* using long reads by using the PacBio DNA sequencing platform and used a genome assembly strategy by taking advantage of genome assembly program Canu^11^. This genome assembly of *O. fasciatus* is the first chromosome-level reference genome constructed for the Oplegnathidae family. The completeness and continuity of the genome will provide high quality genomic resources for studies on sex-determining mechanisms and for accelerating the genome-assisted improvements in resistant breeding systems.

**Genomic DNA extraction****, genome size estimation**

High-quality genomic DNA for sequencing using the Illumina platform (Illumina Inc., San Diego, CA, USA) and PacBio Sequel sequencing (Pacific Biosciences of California, Menlo Park, CA, USA) was extracted from fresh muscle tissue and blood samples from a single female *O. fasciatus*. The fish was collected from the near-shore area of Qingdao city (Yellow Sea), Shandong province. The whole-genome size of *O. fasciatus* was estimated based on Illumina DNA sequencing technology. A short-insert library (300~350 bp) was constructed and generated a total of ~90.7 Gb of raw reads using the standard protocol provided by the Illumina HiSeq X Ten platform (Illumina Inc., San Diego, CA, USA). After the removal of low-quality and redundant reads, we obtained approximately ~80.8 Gb of clean data for *de novo* assembly to estimate the whole-genome size (S Table 1, Fig. 2). All cleaned reads were subjected to 17-mer frequency distribution analysis^12^. As the total number of *k*-mers were approximately 8.09 x 10^10^ and the peak of *k*-mers was at a depth of 100, the genome size of *O. fasciatus* was calculated to be 777.5 Mb using the following formula with amendment: G = (N*_k_*_-mer_ – N_error__*_k_*_-mer_) / D, where G is genome size, N_k-mer_ is the number of *k*-mers, N_error__*_k_*_-mer_ is the number of *k*-mers with the depth of 1, and D is the *k*-mer depth (Fig. 2). Meanwhile, an estimated heterozygosity of 0.29% and a repeat content of 38.46% were detected for *O. fasciatus* in this work. A pilot genome assembly was approximately 744.5 Mb with a contig N50 of 7.2 kb and a scaffold N50 of 84.1kb using the Illumina data and the assembly program Platanus package^13^ (S Table 2). The GC content was 41% (S Fig. 1). This genome assembly was of low-quality, partly due to its high genomic repeat content.

**Genome assembly using PacBio long reads**

Two 20 kb genomic DNA libraries were constructed and sequenced using the PacBio Sequel platform, generating 62.9 Gb raw DNA reads. We obtained 4.8 million subreads (62.8 Gb in total) with an N50 read length of ~22 kb after removing adaptor (S Table 1).

Canu v1.4 was firstly used to assemble the genome with the Corrected-Error-Rate parameter set at 0.040^11^. As a result, a genome assembly with a total length of 875.9 Mb was constructed for *O. fasciatus*, slightly higher than the genome size estimated by 17-mer analysis based on the Illumina data (S Table 2). The genome complexity, such as structural variants and heterozygosity might be possible reasons to explain the relative large genome size in the assembly. We therefore applied Redundans v0.13c^14^ to remove the sequence redundancy to obtain a genome assembly size of 778.0 Mb. We then used the Arrow tool in SMRT Link 5.0 software with the minCoverage parameter set at 15 to implement error correction based on the PacBio long reads data (Table 1). The resulting genome assembly was further polished using Illumina NGS data, which were used in the genome survey analysis above. The final draft genome assembly was 778.7 Mb, which reached a high level of continuity with a contig N50 length of 2.1 Mb (Table 1). The contig N50 of *O. fasciatus* was much higher than those of previous fish genome assemblies constructed using NGS DNA sequencing technologies and is comparable to those of recently reported model fish species (S table 3). Previous studies illuminated the relationship between read length and genome assembly; therefore, we attributed the continuity of the genome primarily to the application of long reads in the assembly.

**Hi-C library construction and chromosome assembly**

Hi-C is a sequencing-based approach for determining chromosome interactions by calculating the contact frequency between pairs of loci, which are strongly dependent upon the one-dimensional distance, in base pairs, between a pair of loci^15, 16^. In this work, we used Hi-C to construct the genome assembly of *O. fasciatus*.

Genomic DNA was extracted for the Hi-C library from a whole-blood sample of *O. fasciatus* as described^17^. Cells were fixed with formaldehyde and lysed, and the cross-linked DNA was digested with MboI. Sticky ends were biotin-labelled and proximity ligated to form chimeric junctions and then physically sheared to a size of 300–500 bp^17^. Chimeric fragments representing the original cross-linked, long-distance physical interactions were then processed into paired-end sequencing libraries, and 629 million 150-bp paired-end Illumina reads (91.5 Gb) were produced with Q20 and Q30 of ~94.0% (S Table 1, S Table 4). By mapping the Hi-C data to the PacBio-based assembly using BWA software, we found that sequencing data with mates mapped to a different contig (or scaffold) and data mapped to a different contig (or scaffold) (map Q5≥ 5) were 593.7 Mb (94.4%), 240.5 Mb (40.5%) and 205.1 Mb (34.6%), respectively (S Table 4). We then further employed BWA and Lachesis software to align paired-end reads to filter all base sequences than 500bp from each restriction site^18^. According to the conduct of clustering, ordering, and orienting to the assembly contigs (1,692), these sequences were grouped into 24 chromosome clusters and scaffolded using Lachesis software with tuned parameters^19^ (S Table 4, Fig. 3). Finally, we constructed the chromosome interactions map using Juicer software and employed the JucieBox to complete the visual correction of the interaction map. We obtained polished 1,756 polished contigs by interrupting misassembly from 1,692 contigs. Twenty-four scaffolds were assembled corresponding to the 24 chromosomes of *O. fasciatus* based on the karyotype analyses ^6, 7^ (S Table 4, Fig. 3).

A final size of 768.8 Mb accounting for the 98.7% draft genome was assembled, which showed a high level of continuity with a contig N50 of 2.1 Mb and a scaffold N50 of 33.5 Mb using 1,372 contigs. The anchor rate of contigs (> 100 kb) to chromosomes was attained up to the 99.7% based on the Hi-C assembly (Table 1). The contig N50 and scaffold N50 of *O. fasciatus* were much higher than those of previous fish genome assemblies constructed using NGS DNA sequencing technologies based on the genome assembly using PacBio long reads and Hi-C assembly (S table 3).

**Genome quality evaluation**

To assess the completeness of the assembled *O. fasciatus* genome, we subjected the assembled sequences to BUSCO version 3 evaluation (BUSCO, actinopterygii_odb9) ^20^. Overall, 96.6% and 1.5% of the 4,584 expected actinopterygii genes were identified in the assembled genome as complete and partial BUSCO profiles, respectively. Approximately 85 genes could be considered missing in our assembly (S table 5). Among the expected complete actinopterygii genes, 4,259 and 171 were identified as single copy and duplicated BUSCOs, respectively (S table 5). We then used Minimap2 to estimate the completeness and homogeneity of genome assembly based on CLR (Continuous Long Reads) subreads. A high quality of completeness and homogeneity was assessed in the genome assembly, and the mapping rate, coverage rate and average sequencing depth reached 90.2%, 99.9% and 80.6, respectively (S table 6). Note that the mapping ratio might be related to the repetitive content of the *O. fasciatus* genome, especially for the high repeat content in the sex chromosomes^6^. However, how the repetitive elements in the genome influence the karyotypes of this species needs further investigation.

To further evaluate the accuracy of the *O. fasciatus* genome assembly, we aligned the NGS-based short reads from the whole-genome sequencing data against the reference genome using BWA^21^. We then used GATK to implement SNP calling and filter work, and the results showed that 99.8% and 0.2% of the 1.6 x 10^6^ expected SNP reads were identified in the assembled genome as heterozygous and homologous SNPs, respectively. SNP calling on the final assembly also yielded a heterozygosity rate of 0.20%, supporting the *k*-mer estimate analysis (0.29%) (S table 7).

**Repeat sequences within th**e ***O. fasciatus*** g**enome assembly**

To identify tandem repeats, we utilized Tandem Repeat Finder to annotate repetitive elements in the *O. fasciatus* genome. RepeatModeler (version 1.04) and LTR_FINDER^22^ were used to construct a *de novo* repeat library with default parameters. Subsequently, we used RepeatMasker^23^ (version 3.2.9) to map our assembled sequences on the Repbase TE (version 14.04) ^24^ and the *de novo* repeat library to identify known and novel transposable elements (TEs). In addition, TE-related proteins were annotated by using RepeatProteinMask software (version 3.2.2) ^23^.

The identified repeat sequences accounted for 33.9% of the *O. fasciatus* genome including repeat sequences with 23.6% of the genome based on the *de novo* repeat library (Table 2). Approximately 23.4% of the *O. fasciatus* genome was identified as interspersed repeats (most often TEs). Among them, DNA transposable elements were the most abundant type of repeat sequences, which occupied 11.5% of the whole genome. Long interspersed nuclear elements (LINEs) and long terminal repeats (LTRs) comprised 7.3% and 4.0% of the whole genome, respectively (Table 2, S Fig. 2).

**RNA preparation and sequencing**

We sequenced cDNA libraries prepared from the eggs of *O. fasciatus* that were used for genome annotation using Illumina sequencing technologies. RNA quality was determined based on the estimation of the ratio of absorbance at 260nm/280nm (OD = 2.0) and the RIN (value = 9.2) by using a Nanodrop ND-1000 spectrophotometer (LabTech, USA) and a 2100 Bioanalyzer (Agilent Technologies, USA), respectively. We used the Clontech SMARTer cDNA synthesis kit to complete reverse transcription. A paired-end library was prepared following the Paired-End Sample Preparation Kit manual (Illumina Inc., San Diego, CA, USA). Finally, a library with an insert length of 300 bp was sequenced by Illumina HiSeq X Ten in 150PE mode (Illumina Inc., San Diego, CA, USA). As a result, we obtained ~42.2 Gb high-quality transcriptome data from RNA-seq (S Table 1, S table 8).

**Gene annotation**

Gene annotation of the *O. fasciatus* genome was performed using *de novo*, homology-based and transcriptome sequencing-based predictions. We employed Augustus (version 2.5.5)^25^ and GenScan (version 1.0)^26^ software to predict protein-coding genes in the *O. fasciatus* genome assembly. Protein sequences of closely related fish species including *Larimichthys crocea*，*Lates calcarifer*，*Gasterosteus aculeatus*，*Paralichthys olivaceus*，*Cynoglossus semilaevis* and *Gadus morhua* were downloaded from Ensembl^27^ and aligned against the *O. fasciatus* genome using TBLASTN software^28^. Subsequently, Genewise2.2.0 software^29^ was employed to predict potential gene structures on all alignments.

We also mapped these NGS transcriptome short reads onto our genome assembly using TopHat1.2 software^30^, and then we employed Cufflinks^31^ to predict gene structures (S table 9). All gene models were then integrated using MAKER to obtain a consensus gene set^32^. The final total gene set was composed of 24,003 genes with an average of 10.1 exons per gene in the *O. fasciatus* genome (Table 1). The gene number, gene length distribution, CDS length distribution, exon length distribution and intron length distribution were all comparable with those of other teleost fish species (S table 9, S Fig. 3).

To obtain further functional annotation of the protein-coding genes in the *O. fasciatus* genome, we employed the local BLASTX and BLASTN programs and the Swiss-prot database with an e-value ≤ 1e-5^33^ to align the non-redundant nucleotide (NT) and non-redundant protein (NR), respectively. We also used Blast2GO software to search the Gene ontology (GO), and Kyoto Encyclopaedia of Genes and Genomes (KEGG) pathway databases^34, 35, 36^. Ultimately, 97.3% (23,364 genes) of the 24,003 genes were annotated by at least one database (S Table 10). Four types of non-coding RNAs (microRNAs, transfer RNAs, ribosomal RNAs, and small nuclear RNAs) were also annotated using the tRNAscan-SE and the Rfam database in this study^37, 38^ (S Table 11).

**Gene family identification and phylogenetic tree construction**

We employed the BLASTP program^39^ with an e-value threshold of 1e-5 to identify gene families based on the transcript alignments of each gene from *O. fasciatus* and other fish species, which included *Larimichthys crocea*, *Gadus morhua*, *Paralichthys olivaceus*, *Cynoglossus semilaevis*, *Notothenia coriiceps*, *Boleophthalmus pectinirostris*, *Lepisosteus oculatus*, *Gasterosteus aculeatus*, *Callorhinchus milii*, *Danio rerio*, *Salmo salar* and *Oryzias latipes*. 21,528 gene families were identified by clustering the homologous gene sequences based on H-scores calculated from Bit-score using Hcluster_sg software (S Fig. 4). Subsequently, we selected 1,236 single-copy orthogroups from the above-mentioned species to construct the phylogenetic relationship between *O. fasciatus* and other fish species. We used the ClustalW program^40^ to extract and align coding sequences of single-copy genes from the 1,158 orthogroups with a length filter (S Fig. 5). All the alignments were concatenated as a single data set for each species. Nondegenerated sites extracted from the data set were then joined into new sequences for each species to construct a phylogenetic tree based on the maximum-likelihood method implemented in the PhyML package^41^ (with the -m PROTGAMMAAUTO model). We used the MCMCtree program to estimate divergence times among species based on the approximate likelihood method^42^ and a molecular clock data from the divergence time between medaka from the TimeTree database^43^. According to the phylogenetic analysis, *O. fasciatus* (Eupercaria: Centrarchiformes) clustered with *Larimichthys crocea* in the order Perciformes (Eupercaria), which was consistent with the new fish species taxonomy^44^ (Fig. 4). The divergence time between *O. fasciatus* and the common ancestor with *Larimichthys crocea* was at approximately 70.5-88.5 Ma.

**Conclusions**

We successfully assembled the genome of *O. fasciatus* and reported the first chromosome-level genome sequencing, assembly and annotation based on long reads from the third-generation PacBio Sequel sequencing platform. The final draft genome assembly is approximately 778.7 Mb, which was slightly higher than the estimated genome size (777.5 Mb) based on *k*-mer analysis. Those contigs were scaffolded to chromosomes using Hi-C data, resulting a genome with a high level of continuity with a contig N50 of 2.1 Mb and a scaffold N50 of 33.5 Mb. The chromosome-level genome assembly of *O. fasciatus* was the first high-quality genome in the Oplegnathidae family. We also predicated 24,003 protein-coding genes from the generated assembly, and 97.3% (23,364 genes) of all protein-coding genes were annotated. We found that the divergence time between *O. fasciatus* and its the common ancestor with *Larimichthys crocea* was approximately 70.5-88.5 Ma. As far as we known, the Y chromosomes has always exhibited many specific sequence characteristics compared to X1 and X2, such as repeat content, and those differences might increase the difficulty of the sequence assembly of chromosomes X1 and X2. The chromosome-level genome assembly together with gene annotation data generated for the female fish in this work will provide a valuable resource for further research on sex-determining mechanisms, especially for obtaining an accurate assembly of the Y chromosome in male fish. These results will also accelerate genome-wide association studies in resistant breeding systems.

**Ethics Statement**

This research was approved by the Animal Care and Use committee of Chinese Academic Science. All participates consent the study under the 'Ethics, consent and permissions' heading. All participants consent to publish the work under the 'Consent to publish' heading.

**Availability of supporting data**

Supporting data and materials are available in the GigaScience GigaDB database, with the raw sequences deposited in the SRA under the accession number SRP158313 and SRP160016^45^.

**Competing interests**

The authors declare that they have no competing interests.

**Funding**

This study was supported by a grant from the National Natural Science Foundation of China (No. 41506170, No. 31672672, and No. 31872195), Shandong Province Key Research and Invention Program (2017GHY15102, 2017GHY15106), Qingdao Source Innovation Program (17-1-1-57-jch), Marine Fishery Institute of Zhejiang Province, Key Laboratory of Mariculture and Enhancement of Zhejiang Province (2016KF002), National Key Research and Development Program (2018YFD0901204), STS project (KFZD-SW-106, ZSSD-019), Qingdao National Laboratory for Marine Science and Technology (2015ASKJ02), China Agriculture Research System (CARS-47).

**Author Contributions**

YSX conceived the project. ZZX, DYM collected the samples and extracted the genomic DNA. YSX, JL and JL performed the genome assembly and data analysis. YSX, ZZX, JL, DYM and JL wrote the paper.

**Reference**

1. Schembri PJ, Bodilis P, Francour P, et al. Occurrence of barred kinfejaw, *Oplegnathuf fasciatus* (Actinopterygii: Perciformes: Oplegnathidae), in Malta (Central Mediterranean) with a discussion on possible modes of entry. *Acta Ichthyol Piscat*. 2010; 40:101-104.
2. Mundy BC. Checklist of the fishes of the Hawaiian Archipelago. *Bishop Mus Bull Zool*. 2005; 6: 1-704.
3. An HS, Hong SW. Genetic diversity of rock bream *Oplegnathus fasciatus* in Southern Korea. *Genes Genom*. 2008; 30: 451-459.
4. Xiao YS, Li J, Ren GJ, et al*.* Pronounced population genetic differentiation in the rock bream *Oplegnathus fasciatus* inferred from mitochondrial DNA sequences. *Mitochondrial DNA A*. 2016; 27: 2045-2052 ().
5. Park HS, Kim CG, Par YJ, et al. Population Genetic Structure of Rock Bream (*Oplegnathus fasciatus* Temminck & Schlegel, 1844) Revealed by mtDNA COI Sequence in Korea and China. *Ocean Sci J*. 2018; 53: 261-274.
6. Xu DD, Lou B, Bertollo LAC, et al. Chromosomal mapping of microsatellite repeats in the rock bream fish *Oplegnathus fasciatus*, with emphasis of their distribution in the neo-Y chromosome. *Mol Cytogenet*. 2013; 6: 12.
7. Xue R, An H, Liu QH, et al*.* Karyotype and Ag-Nors In Male And Female Of *Oplegnathus Punctatus*. *Oceanol Limnol Sin*. 2016; 47: 626-632.
8. Xiao ZZ. Study on population genetics and culture biology of *Oplegnathus fasciatus*. Doctor thesis. 2015. P. 162-176.
9. Zhang BC, Zhang J, Xiao ZZ, et al. Rock bream (*Oplegnathus fasciatus*) viperin is a virus-responsive protein that modulates innate immunity and promotes resistance against megalocytivirus infection. *Dev Comp Immunol*. 2014; 45: 35-42.
10. Li H, Sun ZP, Jiang YL, et al. Characterization of an Iridovirus Detected in Rock Bream (*Oplegnathus fasciatus*; Temminck and Schlegel). *Chin J Virol*. 2011; 27: 158-164.
11. Koren S, Walenz BP, Berlin K, et al*.* Canu: scalable and accurate long-read assembly via adaptive k-mer weighting and repeat separation. *Genome Res*. 2017; 27: 722.
12. Marcais G, Kingsford C. A fast, lock-free approach for efficient parallel counting of occurrences of k-mers. *Bioinformatics*. 2011; 27: 764-70.
13. Kajitani R, Toshimoto K, Noguchi H, et al*.* Efficient de novo assembly of highly heterozygous genomes from whole-genome shotgun short reads. *Genome Res*. 2014; 24: 1384-1395.
14. Pryszcz LP, Gabaldón T. Redundans: an assembly pipeline for highly heterozygous genomes. *Nucleic Acids Res*. 2016; 44: e113-e113.
15. Simão FA, Waterhouse RM, Ioannidis P, et al. BUSCO: assessing genome assembly and annotation completeness with single-copy orthologs. *Bioinformatics*. 2015; 31: 3210.
16. Li H, Durbin R. Fast and accurate short read alignment with Burrows–Wheeler transform. *Bioinformatics*. 2009; 25: 1754-1760.
17. Benson G. Tandem repeats finder: a program to analyze DNA sequences. *Nucleic Acids Res* 1999; 27: 573-580.
18. Tarailo-Graovac M, Chen NS. Using RepeatMasker to identify repetitive elements in genomic sequences. In: Editoral board, Baxevanis Andreas D et al. (eds.), Current Protocols in Bioinformatics, 2009. p. 5: 4.10.1-4.10.14.
19. Jurka, J, Kapitonov VV, Pavlicek A, et al*.* Repbase Update, a database of eukaryotic repetitive elements. *Cytogenetic Genome Res*. 2005; 110: 462-467.
20. Stanke M, Steinkamp R, Waack S, et al. AUGUSTUS: a web server for gene finding in eukaryotes. *Nucleic Acids Res*. 2004; 32: 309-312.
21. Cai Y, González JV, Liu Z, et al*.* Computational systems biology methods in molecular biology, chemistry biology, molecular biomedicine, and biopharmacy. *BioMed Res Int.* 2014; 746814.
22. Flicek P, Amode MR, Barrell D, et al. Ensembl 2014. *Nucleic Acids Res*. 2014; 42: D749-D755.
23. Gertz EM, Yu YK, Agarwala R, et al*.* Composition-based statistics and translated nucleotide searches: Improving the TBLASTN module of BLAST. *MBC Biology*. 2006; 4: 41.
24. Birney E, Clamp M, Durbin R, et al. GeneWise and Genomewise. *Genome Res*. 2004; 14: 988-995.
25. Trapnell C, Pachter L, Salzberg SL, et al*.* TopHat: discovering splice junctions with RNA-Seq. *Bioinformatics.* 2009; 25: 1105-1111.
26. Ghosh S, Chan CKK. Analysis of RNA-Seq data using TopHat and Cufflinks. *Methods Mole Biol*. 2016; 1374.
27. Campbell MS, Holt C, Moore B, et al. Genome Annotation and Curation Using MAKER and MAKER-P. *Current Protocols in Bioinformatics*. 2014; 48: 4.11.11.
28. Lobo I. Basic Local Alignment Search Tool (BLAST). *J Mol Biol.* 2008; 215: 403-410.
29. Harris MA, Clark J, Ireland A, et al*.* The Gene Ontology (GO) database and informatics resource. *Nucleic Acids Res*. 2004; 32: D258-D261.
30. Ogata H, Goto S, Sato K, et al*.* KEGG: Kyoto Encyclopedia of Genes and Genomes. *Nucleic Acids Res*. 1999; 27: 29-34.
31. Conesa A, Götz S, García-Gómez JM, et al*.* Blast2GO: a universal tool for annotation, visualization and analysis in functional genomics research. *Bioinformatics*. 2005; 21: 3674.
32. Lowe TM, Eddy SR. tRNAscan-SE: a program for improved detection of transfer RNA genes in genomic sequence. *Nucleic Acids Res*. 1997; 25: 955-964.
33. Griffiths-jones S, Bateman A, Marshall M, et al*.* Rfam: an RNA family database. *Nucleic Acids Res*. 2003; 31: 439.
34. Lieberman-Aiden E, Van Berkum NL, Wiiliams L, et al. Comprehensive mapping of long-range interactions reveals folding principles of the human genome. *Science*. 2009; 326: 289-293.
35. Rao SSP, Huntley MH, Durand NC, et al*.* A 3D map of the human genome at kilobase resolution reveals principles of chromatin looping. *Cell*. 2014; 159: 1665-1680.
36. Sandborn AL, Rao SSP, Huang SC, et al. Chromatin extrusion explains key features of loop and domain formation in wild-type and engineered genomes. *Proc Natl Acad Sci USA*. 2015; 112: E6456.
37. Flot JF, Marie-Nelly H, Koszul R, et al. Contact genomics: scaffolding and phasing (meta) genomes using chromosome be 3D physical signatures. *FEBs Letters*. 2015; 589: 2966-2974.
38. Burton JN, Adey A, Patwardhan RP, et al. Chromosome-scale scaffolding of de novo genome assemblies based on chromatin interactions. *Nat Biotechnol*. 2013; 31: 1119-1125.
39. Lobo I. Basic Local Alignment Search Tool (BLAST). *J Mol Biol*. 2008; 215: 403-410.
40. Thompson JD, Gibson TJ, Higgins DG, et al*.* Multiple sequence alignment using ClustalW and ClustalX. *Curr Protoc Bioinformatics*. 2002; 2.3. 1-2.3. 22.
41. Guindon S, Dufayard JF, Hordijk W, et al*.* PhyML: Fast and Accurate Phylogeny Reconstruction by Maximum Likelihood. *Infect Genet Evol*. 2009; 9: 384-385.
42. Yang Z, Rannala B. Bayesian estimation of species divergence times under a molecular clock using multiple fossil calibrations with soft bounds. *Mol Biol Evol*. 2006; 23: 212-226.
43. Hedges SB, Marin J, Suleski M, et al*.* Tree of life reveals clock-like speciation and diversification. *Mol Biol Evol.* 2015; 32: 835-845.
44. Betancur-R R, Wiley E, Bailly N, et al. Phylogenetic classification of bony fishes. *BMC Evol Biol*. 2017; 17: 162.
45. Xiao YS, Xiao ZZ, Ma DY, et al. Supporting data for "Genome sequence of the barred knifejaw Oplegnathus fasciatus (Temminck & Schlegel, 1844): the first chromosome-level draft genome in the family Oplegnathidae". GigaScience Database. 2019. <http://dx.doi.org/10.5524/100556>.
