# Supplementary material for: Genome sequence of the barred knifejaw Oplegnathus fasciatus (Temminck & Schlegel, 1844): the first chromosome-level draft genome in the family Oplegnathidae
Source: Gigascience. 2019 Feb 1;8(3):giz013. doi: 10.1093/gigascience/giz013 (PMC6423371; doi:10.1093/gigascience/giz013)
Supplement: Response_to_Reviewer_Comments_Original_Submission.pdf [file giz013_response_to_reviewer_comments_original_submission.pdf]

Author's Response To Reviewer Comments

Close

GIGA-D-18-00300
Genome sequence of rock bream, Oplegnathus fasciatus (Temminck & Schlegel, 1884): the first draft genome in family Oplegnathidae
Yongshuang Xiao; Zhizhong Xiao; Jing Liu; Daoyuan Ma; Jun Li
GigaScience

Dear Dr. Xiao,

Your manuscript "Genome sequence of rock bream, Oplegnathus fasciatus (Temminck & Schlegel, 1884): the first draft genome in family Oplegnathidae" (GIGA-D-18-00300) has been assessed by our reviewers. Although it is of interest, we are unable to consider it for publication in its current form. The reviewers have raised a number of points which we believe would improve the manuscript and may allow a revised version to be published in GigaScience. In particular it needs significant editing by a native English speaker as the language needs a lot of work. Please also include details on common names of the species and NCBI taxon/Fishbase IDs, and other identifiers in the paper.
Reply:
We would like to give sincere thanks to the editor's suggestions. In order to check the accurate species information, we have checked the taxonomy information from the WORMS (World Register of Marine Species) http://www.marinespecies.org/aphia.php?p=search, Wikipedia https://www.wikipedia.org/, NCBI https://www.ncbi.nlm.nih.gov/Taxonomy/Browser/wwwtax.cgi?id=163133 and Fishes of the World (Fifth Editon) (Joseph S. Nelson, Terry C. Grande and Mark V. Wilson), and all of them supported the taxonomy of the Oplegnathidae. The Oplegnathidae occupied one genus composed of seven species Oplegnathus conwayi (Richardson, 1840), Oplegnathus fasciatus (Temminck & Schlegel, 1844), Oplegnathus insignis (Kner, 1867), Oplegnathus peaolopesi (Smith, 1947), Oplegnathus punctatus (Temminck & Schlegel, 1844), Oplegnathus robinsoni (Regan, 1916), Oplegnathus woodwardi (Waite, 1900).

Their reports, together with any other comments, are below. Please also take a moment to check our website at https://giga.editorialmanager.com/ for any additional comments that were saved as attachments.

If you are able to fully address these points, we would encourage you to submit a revised manuscript to GigaScience. Once you have made the necessary corrections, please submit online at:

https://giga.editorialmanager.com/

If you have forgotten your username or password please use the "Send Login Details" link to get your login information. For security reasons, your password will be reset.

Please include a point-by-point within the 'Response to Reviewers' box in the submission system. Please ensure you describe additional experiments that were carried out and include a detailed rebuttal of any criticisms or requested revisions that you disagreed with. Please also ensure that your revised manuscript conforms to the journal style, which can be found in the Instructions for Authors on the journal homepage.

The due date for submitting the revised version of your article is 08 Jan 2019.

I look forward to receiving your revised manuscript soon.

Best wishes,

Hongling Zhou
GigaScience
www.gigasciencejournal.com

Reviewer reports:

Reviewer #1: This manuscript describes the genome assembly and annotation of O. fasciatus, with little else by way of analysis. The methods used are mostly appropriate, and the assembly appears to be of high quality.

Some issues and suggestions:

1. The assembly contiguity is repeatedly referred to as 'remarkable', this is perhaps an exaggeration. These values are not extraordinary in the age of long-read sequencing. S Table 4 lists other fish assemblies, but includes almost no current-generation ones, flattering the assembly statistics obtained in this study.
Reply:
We would like to give sincere thanks to the reviewer’s suggestions. We have thoroughly revised the manuscript for the description of the quality of the genome assembly. And we have deleted the degree word of “remarkable” as follows:
1) We revised the “which reached a remarkable high level of continuity with contig N50 of 2.1 Mb” as “which reached a high level of continuity with a contig N50 of 2.1 Mb” .
2) We revised the “which reached a remarkable high level of continuity with contig N50 length of 2.1 Mb” as “which reached a high level of continuity and a contig N50 of 2.1 Mb” .
3) We revised the “which showed a remarkable high level of continuity with contig” as “which showed a high level of continuity with a contig”.
4) We revised the “which reached a remarkable high level of continuity with contig” as “which reached a high level of continuity with a contig”.
5) We revised the “The contig N50 was remarkable longer than those of most fish” as “Contig N50 was longer than those of most fish”.

Line 336-338: Meanwhile, we have highlighted that the important role of long reads in the contig continuity of genome assembly in the test as follows: “Previous studies illuminated the relationship between read length and genome assembly; therefore, we attributed the continuity

of the genome primarily to the application of long reads in the assembly”.
Table 4: According to the reviewer’s comments, we also added the current-generation of other fish assemblies in the Table 4, which included Lepisosteus oculatus (Genome Size: 945 Mb, Contig N50: 0.07Mb, Scaffold N50: 6.9Mb), Sillago sinica (Genome Size: 534 Mb, Contig N50: 2.6Mb), Lates calcarifer (Genome Size: 586 Mb, Contig N50: 1.07Mb, Scaffold N50: 25.85Mb), Oreochromis niloticus (Genome Size: 868 Mb, Contig N50: 3.3Mb, Scaffold N50: 37Mb).

2. I will admit I am not an expert on Oplegnathidae. However, according to Wikipedia, the genus Oplegnathus contains seven species, and the common name for O. fasciatus is 'striped beakfish' or 'barred knifejaw'. The manuscript claims two species (line 68), and the common name 'rock bream'.
Reply:
We agreed with the reviewer’s comment on the taxonomy of the Oplegnathidae. The Oplegnathidae occupied one genus composed of seven species Oplegnathus conwayi (Richardson, 1840), Oplegnathus fasciatus (Temminck & Schlegel, 1844), Oplegnathus insignis (Kner, 1867), Oplegnathus peaolopesi (Smith, 1947), Oplegnathus punctatus (Temminck & Schlegel, 1844), Oplegnathus robinsoni (Regan, 1916), Oplegnathus woodwardi (Waite, 1900). We have checked the taxonomy information from the WORMS (World Register of Marine Species) http://www.marinespecies.org/aphia.php?p=search, Wikipedia https://www.wikipedia.org/, NCBI https://www.ncbi.nlm.nih.gov/Taxonomy/Browser/wwwtax.cgi?id=163133 and Fishes of the World (Fifth Editon) (Joseph S. Nelson, Terry C. Grande and Mark V. Wilson), and all of them supported the taxonomy of the Oplegnathidae.
It’s our mistake in the text for the verification of species numbers. We know two (Oplegnathus fasciatus and O. punctatus) of seven species existed in the coastal waters of East Asia.
We also checked the common name of O. fasciatus, the common name of rock bream is incorrect and we revised it as “barred knifejaw” based on the reviewer’s comments, NCBI and Wikipedia. We also revised it in the text.
We used the common name “barred knifejaw” instead of “rock bream” in the text.

3. Line 109: 'a repeat content of 38.46%', how was this calculated? It does not follow from figure 2.
Reply: The K-mer distribution from the sequencing data could be used for the genome size, heterozygosity and repeat content ratio estimation, mainly from the relative numbers of homozygous, heterozygous and repeated Kmers, using the statistical model described in the previous study (Liu, B. et al. Estimation of genomic characteristics by analyzing k-mer frequency in de novo genome projects. Quantitative Biology 35, 62-67 (2013)). We have illuminated the peaks raised by homozygous, heterozygous and repeated K-mers in Figure 2.

4. Line 107/111: The k-mer estimate and the intial assembly yield exactly the same genome size (808.9 Mbp). This is highly unlikely, especially if the genome is highly repetitive, as claimed here.
Reply:
We would like to give sincere thanks to reviewer’s suggestions. We have carefully checked our

sequencing results and found there was a clerical error in the text.
Line 286: According to the estimation of K-mer, the genome size is 786.46Mb, and after eliminating the influence of K-mer error, we get the genome size is 777.5Mb.
Line 289: According to the assembly of platanus, the contig N50 is 7.19kb with total length of 875.4Mb. And then reached to the level of scaffold N50 is 84.126kb with total length of 744.53Mb.
So, it is a clerical error in the text, and we have revised them in the text.

5. Line 123: I assume the contigs and scaffold listed here, to which the HiC data map, are those of the final (PacBio-based) assembly. However, the only assembly that has been described at this point is the highly fragmented initial one. Perhaps you could restructure this so that the HiC sequencing is described after PacBio sequencing.
Reply:
The reviewer is correct. The genome used here for the Hi-C data evaluation is the genome assembled from the PacBio sequencing data. We moved this part after the PacBio sequencing data assembly.

6. Figure 2 shows a clear bump corresponding to duplicated k-mers (at 200). Is this duplication level still relevant for the final assembly? For example, a lot of sequence is removed (line 136) based on redundancy, and a large fraction of PacBio reads do no map to the final assembly (line 158). Is there a relation with the sex chromosome configuration (X1X1X2X2, line 76)?
Reply: We agree with the reviewer's comment on the repeat content of the final assembly. We noticed that the repeat content of final genome were about 33.9%, which was lower than that from the genome survey estimation (38.5%). The high repetitive elements in repeated regions of chromosomes, such as those in the sex chromosome, might result into fragmented assembly. Those repeated sequences might be removed in the redundancy elimination process. We have added the discussion into our revised manuscript as follows:
Line 437-440: "Note that the mapping ratio might be related to the repetitive content of the O. fasciatus genome, especially for the high repeat content in the sex chromosomes6. However, how the repetitive elements in the genome influence the karyotypes of this species needs further investigation."

7. Line 140: That the polishing is performed using Pilon should be mentioned here (it is mentioned in S table 2). Also, 'using NGS data' is ambiguous, as PacBio also qualifies as NGS. This probably refers to Illumina only.
Reply: Thanks a lot for the reviewer's suggestion. We have added the description of Pilon for the sequence polishing in the manuscript. "Using NGS data" referred to Illumina data only here, we therefore clarify the sentence in the manuscript as follows: "The resulting genome assembly was further polished using Illumnina NGS data"

8. Figure 4 and S Figure 4 analyze O. fasciatus in the context of 'fish species'. While this is technically correct, it is biologically not always the most relevant comparison. Fish species such as ghost shark and lancelet are included, but for example tetrapods (which are more closely related to O. fasciatus than the aforementioned fish) are not. In figure 4, these make for less appropriate outgroups (because of their very distant relationship to the other, teleost, fish

species). I would suggest including at least e.g. spotted gar to the analysis to fill this gap (and perhaps omit B. floridae).
Reply: We agree with the reviewer. We have added the spotted gar and deleted B. floridae in our phylogenetic analysis, and re-preformed the gene family construction and phylogenetic analysis. The result of phylogeny including the spotted gar was consistent with reviewer's prospection, which filled the gap from the fish evolution process in our study. We would like to give sincere thanks to reviewer's suggestions. The revised phylogenetic results were illuminated in the Figure 4.

8. Figure 4 needs more information in the legend. What do the numbers mean exactly, and how were they calculated? The conclusion drawn from this figure (line 266) is not appropriate, as the phylogenetic position of Notothenioidei is not relevant to the narrative of this manuscript, and reclassification needs more evidence than this sparse phylogenetic tree.
Reply: Thanks a lot for the reviewer's suggestion. We have added more information in the legends for the Figure 4. The descriptions of the phylogenetic analysis were revised in the manuscript. And we agree with the reviewer's suggestion that the phylogenetic position of Notothenioidei is not relevant to the narrative of our manuscript and we deleted it.

9. One of the motivations for sequencing this genome is understanding the fish' sex determining system. This aim is not revisited in the results or Conclusion. How does the choice of a female individual for genome sequencing affect this goal?
Reply:
Thanks a lot for the reviewer's suggestion. We have added the sentence for the importance of genome in our following genetic studies to understand the sex-determining system of the fish species. The reason we chose a female one for the genome assembly because the female ones do not have heterotropic chromosome, which might facilitate the chromosome assembly of X1 and X2. The quality of X1 and X2 could lay a solid foundation for the chromosome analysis in our following studies.
We have added the discussion in the conclusion as follows: "As far as we known, the Y chromosomes has always exhibited many specific sequence characteristics compared to X1 and X2, such as repeat content, and those differences might increase the difficulty of the sequence assembly of chromosomes X1 and X2. The chromosome-level genome assembly together with gene annotation data generated for the female fish in this work will provide a valuable resource for further research on sex-determining mechanisms, especially for obtaining an accurate assembly of the Y chromosome in male fish. These results will also accelerate genome-wide association studies in resistant breeding systems."

Typos:
L 102 Hieq -> HiSeq
L 172 RepreatMasker -> RepeatMasker
Reply: Thanks for the reminding from the reviewer. We have revised it in the text.

Reviewer #2: In this manuscript Xiao et al. reports the genome assembly of the rock bream (O. fasciatus) a species of increasing economic importance in Asia. This species exhibits sex

dimorphism in growth and also a sex determination system based on multiple sex chromosomes X1X1X2X2/X1X2Y, which makes it interesting species to study. The draft genome of the rock bream will be a valuable resource to facilitate future research aimed at improving relevant traits and understanding of determination systems.
The authors used an adequate amount of sequence data from three different sources (Illumina short reads, PacBio and Hi-C), which allowed them to generate a robust genome assembly. Furthermore, the authors annotated the genome using multiple strategies. Finally, they carried out some phylogenetic analyses including other fish species. The methods followed to obtain the assembly are good in general, and well described.

L33-L35 Please re-phrase, maybe say "sexual dimorphism in growth"
Reply: Thanks a lot for the reviewer's suggestion
Line 37-38 According to the reviewer's comments, we revised the "growth of sexual dimorphism with neo-sex chromosome and widespread biotic" as "sexual dimorphism in growth with neo-sex chromosome and widespread biotic".
L37 ...basing -> based
Reply: Thanks a lot for the reviewer's suggestion, we have revised the manuscript in the text. We revised the "basing" as "based".

L43 "…We assembled the O.fasciatus"
Reply: Thanks a lot for the reviewer's suggestion, we have revised the manuscript in the text. We added the "genome" after the "O.fasciatus"

L77 Again please re-phrase "the growth sexual dimorphism"
Reply: Thanks a lot for the reviewer's suggestion, we have revised the manuscript in the text. We revised the "the growth sexual dimorphism" as "sexual dimorphism in growth".
L99-L100 "A whole genome using Illumina DNA seq…" re-phrase
Reply: According to the reviewer's comments, we have revised the manuscript i in the text. We revised the "A whole-genome using Illumina DNA sequencing technology was applied to estimate O. fasciatus genome size." as "The whole-genome size of O. fasciatus was estimated based on the Illumina DNA sequencing technology".

L115 Was the blood extracted from the same fish used for pacBio and Illumina?
Reply: Thanks a lot for the reviewer's question. In order to avoid the genetic-background influence of individual difference, expecially for the HI-C result, the blood was extracted from the same female fish of O. fasciatus used for pacBio and Illumina.

L162 "the results showed 99.8%.."
Reply: Thanks a lot for the reviewer's suggestion, we have revised the manuscript in the text. We added the "that" after the "showed" as "the result showed that 99.8%......"

L172 Typo Repreatmasker
Reply: We have revised the manuscript in the text. We revised the "Repreatmasker" as "RepeatMasker".

L252 Is not clear how you came up to those 812 orhtogroups, and the same for L256

Reply: 21,528 gene families were constructed from the gene family clustering. However, most of the gene families contained more than one gene for species in our studies. To eliminate uncertain effects for the phylogenetic analysis from duplicated genes, we only selected gene families that contain one and only one genes for each species. In our case we obtained 1236 gene families (1236 genes) for the phylogenetic analysis. After removing short gene (length shorter than 100 amino acid (about 300bp)), we obtained 1158 genes for the final anlaysis.

L266 I don't think the authors should claim that the Notothenioidei should be elevated to the order level, but I would accept that their results suggest or show evidence of this.
Reply: Thanks a lot for the reviewer's suggestion. We have revised our conclusion from the phylogenetic analysis. Indeed, we cannot claim the phylogenetic position of Notothenioidei from our data, but our result could provide useful knowledge for the related studies. We think that the phylogenetic position of Notothenioidei is not relevant to the narrative of our manuscript and we deleted it.

General Comments:
There are many issues with the English throughout the manuscript and these must be addressed before considering for publication. I strongly encourage the authors to proof-read the manuscript before re-submitting.
Reply: Thanks for the editor's suggestion. We have revised the English throughout the manuscript with the service of AJE (American Journal Experts). We hoped that the English now could meet the standard for the GigaScience. The revision places as follows:
Line 1 we revised "Genome sequence of barred knifejaw,…" as "Genome sequence of the barred knifejaw,…".
Line 3 we revised "the first draft genome in family Oplegnathidae" as "the first chromosome-level draft genome in the family Oplegnathidae".
Line 33 we revised "The barred knifejaw (Oplegnathus fasciatus),…" as "The barred knifejaw Oplegnathus fasciatus,…".
Line 34 we revised "commerically" as "commercially".
Line 38 we revised "has received" as "has been received".
Line 39-40 we revised the sentence "However, the adequate genome resources to make insight into sex-determining mechanism and to establish genetically based resistant breeding systems for O. fasciatus have been lacking. " as "However, adequate genome resources for gaining insight into sex-determining mechanisms and establishing genetically based resistant breeding systems for O. fasciatus are lacking.".
Line 41-43 we revised the sentence "we performed whole genome of female fish for O. fasciatus using long-read sequencing and Hi-C data to generate chromosome-length scaffolds with highly contiguous genome assembly." as "we analysed the entire genome of a female O. fasciatus fish using long-read sequencing and Hi-C data to generate chromosome-length scaffolds and a highly contiguous genome assembly."
Line 45 we revised ", which" as "that".
Line 46 we revised "both of" as "both the". And we also revised the "Hiseq" as "HiSeq".
Line 48 we added "a" in front of "contig N50".
Line 49-53 we revised the sentence as "We combined Hi-C data with a draft genome assembly to generate chromosome-length scaffolds. Twenty-four scaffolds corresponding to the twenty-four chromosomes were assembled to a final size of 768.8 Mb with a contig N50 of 2.1 Mb and

a scaffold N50 of 33.5 Mb using 1,372 contigs." .
Line 53 we revised "account" as "accounted".
Line 55 we revised "annotated using de novo method and" as "annotated using de novo methods, ".
Line 55 we revised "homologies" as "homology". We also revised "with draft" as "with a draft".
Line 56 we deleted both of "the" and "the".
Line 57-58 we revised the sentence "was close related to Larimichthys crocea and O. fasciatus diverged from their ancestor was at about 70.3-87.3 million years ago." as "is closely related to Larimichthys crocea, with O. fasciatus diverging from their common ancestor approximately 70.3-87.3 million years ago".
Line 60 we revised the sentence "We generated high-quality draft genome and chromosomes assembly" as "We generated a high-quality draft genome with chromosome assembly".
Line 146we revised "is" as "represents".
Line 147-149 we revised the sentence "The genome assembly will provide insight into sex-determining mechanism and serve as a resource for accelerating the genome-assisted improvement of resistant breeding systems." as "Assembly of this genome will provide insight into sex-determining mechanisms and serve as a resource for accelerating genome-assisted improvements in resistant breeding systems.".
Line 154 we revised "The family Oplegnathidae belongs" as "The Oplegnathidae family".
Line 155 we revised "including only one genus Oplegnathus comprised of" as "including only one genus Oplegnathus, which is comprised of".
Line 156 we revised "two (O. fasciatus and O. punctatus) of which" as "two of which (O. fasciatus and O. punctatus)".
Line 157 we revised "commercial values in East Aisa" as "commercially valuable in East Asia".
Line 158 we deleted "," in both sides of "O. fasciatus (Temminck & Schlegel, 1844)".
Line 158 we revised "the two" as "these two".
Line 159 we revised "meters" as "metres".
Line 160 we revised "being distributed in" as "and distributed across".
Line 163-164 we revised "It was reported that the male of Oplegnathus has a neo-sex chromosome" as "It has been reported that the male Oplegnathus possesses a neo-sex chromosome".
Line 164 we revised ", and the" as ". The".
Line 165 we revised "was" as "is".
Line 166 we deleted "the" in front of "karyotype analyses".
Line 166 we revised the "was" as "has been".
Line 167 we revised "and the male fish showed a faster growth advantage than the female" as ", with male fish exhibiting faster growth than females". We also revised "may" as " possibly".
Line 168 we revised "of" as "in".
Line 171 we revised "for making" as "to gain". We also revised "accelerating" as "to accelerate".
Line 172 we revised "improvement of" as "improvements in".
Line 173 we revised "So far, the genome sequence with the chromosomes assembly" as "So far, a genome sequence with the chromosomal assembly".
Line 263 we revised "Here we performed" as "Here, we constructed".

Line 264 we deleted “constructed”.
Line 265 we revised “using” as “used”.
Line 266 we revised “assemblyer Canu” as “assembly program Canu”. We also revised “the” as “this”.
Line 267 we revised “the family Oplegnathidae” as “the Oplegnathidae family”.
Line 270 we revised “improvement of” as “improvements in”.
Line273 we added “sequencing using” in front of “the Illumina platform”.
Line 276 we revised “sample of” as “samples from”.
Line 277 we deleted “the”.
Line 280 we added “the” in front of “Illumina HiSeq X Ten platform”.
Line 281 we added “the” before “removal of low-quality and redundant reads”.
Line 282 we revised “about” as “approximately”.
Line 283 we deleted “the” in front of “cleaned reads”.
Line 284 we revised “about” as “approximately”.
Line 285 we added “was” in front of “at a depth of 100”.
Line 287 we revised “the” as “an”.
Line 290 we revised “contig N50 7.2 kb and scaffold N50 84.1kb” as “contig N50 of 7.2 kb and a scaffold N50 of 84.1kb”.
Line 292 we added “,” in front of “partly due to”. We also revised “genomics” as “genomic”.
Line 317 we added “the” in front of “PacBio”.
Line 318 we revised “obtain” as “obtained”.
Line 319 we revised “totally 62.8 Gb” as “62.8 Gb in total”. We also revised “a read N50” as “an N50 read”.
Line 321 we revised “The Canu” as “Canu”.
Line 322-323 we revised “As a result, a total length of 875.9 Mb genome assembly was achieved for O. fasciatus” as “As a result, a genome assembly with a total length of 875.9 Mb was constructed for O. fasciatus”.
Line 323 we deleted “which was”.
Line 324 we revised “the estimated genome size in 17-mer analysis” as “the genome size estimated by 17-mer analysis”.
Line 325 we revised “relative” as “relatively”.
Line 325-326 we revised “the complexity of genome such as heterozygosity” as “the complexity of this genome to factors such as heterozygosity”.
Line 327 we revised “and obtain genome” as “to obtain a”.
Line 328 we revised “the Arrow of Smrtlink 5.0” as “the Arrow tool in SMRT Link 5.0 software”.
Line 329 we deleted “the” in front of “the error correction”.
Line 335 we revised “technologies, and is comparable with” as “technologies and is comparable to”.
Line 341-342 we revised “depended strongly on” as “are strongly dependent upon”.
Line 395-396 we revised the sentence “The genomic DNA for Hi-C library was extracted from the whole-blood cell of O. fasciatus as described” as “Genomic DNA was extracted for the Hi-C library from a whole-blood sample of O. fasciatus as described”.
Line 397 we revised “digested” as “was digested”.
Line 397 we revised “biotin-labeled” as “biotin-labelled”.
Line 401 we added “were produced” in front of “with Q20 and”.

Line 402 we added “the” in front of “Hi-C data”.
Line 407 we revised “other” as “more”.
Line 409 we revised “those” as “these sequences”.
Line 411 we revised “interactions map” as “the interaction map”.
Line 413 we revised “contigs” as “polished contigs”.
Line 414 we added “were assembled” in front of “corresponding to”.
Line 419 we revised “reached” as “attained”.
Line 427 we added “assembled” in front of “sequences”.
Line 431 we deleted “both of”.
Line 433 we deleted “the” in front of “Minimap2”.
Line 434 we deleted “the” in front of “CLR”.
Line 435 we revised “checked for” as “assessed in the”.
Line 436-437 we revised “sequencing depth were reached to 90.2%, 99.9% and 80.6” as “sequencing depth reached 90.2%, 99.9% and 80.6”.
Line 441-442 we added “the” in front of “O. fasciatus” and “whole-genome” respectively.
Line 443 we deleted “the” in the front of “GATK”. We also deleted “the” in front of “SNP”.
Line 444 we revised “the result” as “and the results”.
Line 445 we revised “heterozygosis and homology” as “heterozygous and homologous”.
Line 446 we revised “yield” as “yielded”.
Line 447 we revised “the estimate from k-mer” as “the k-mer estimate analysis”.
Line 449 we revised “Repeat sequence” as “Repeat sequences”.
Line 502 we deleted “the” in front of “TE-related proteins”.
Line 504 we revised “account” as “accounted”.
Line 505 we revised “included” as “including”.
Line 509 we revised “The long interspersed nuclear elements (LINE) and long terminal repeat (LTR)” as “Long interspersed nuclear elements (LINEs) and long terminal repeats (LTRs)”.
Line 510 we revised “took up 7.3% and 4.0% of the whole genome” as “comprised 7.3% and 4.0% of the whole genome, respectively”.
Line 512 we added “that were” in front of “used for”.
Line 513-514 we revised “High quality of RNA were detected” as “RNA quality was determined”.
Line 514 we added “ratio of ” in front of “absorbance”.
Line 515 we added “using a” in front of “Nanodrop ND-1000”.
Line 516 we added “a” in front of “2100 Bioanalyzer”.
Line 517 we deleted “the process of” in front of “reverse transcription”.
Line 518 we revised “The” as “A”. We also deleted “the manual of” in front of “the Paired-End Sample”.
Line 519 we revised “the library” as “a library”.
Line 525 we revised “prediction” as “predictions”.
Line 527 we revised “of” as “in the”.
Line 530 we revised “to” as “the”.
Line 583 we deleted “the”.
Line 586 we revised “then we” as “we then”. We also deleted “the” in front of “gene”.
Line 589 we added “the” in front of “O. fasciatus genome”.
Line 591 we revised “in” as “of”.
Line 593-596 we revised the sentence “In order to further obtain functional annotation of the

protein-coding genes in O. fasciatus genome, we employed local BLASTX and BLASTN programs to align upon the non-redundant protein (NR), non-redundant nucleotide (NT) and Swissprot database with an e-value ≤ 1e-5" as "To obtain further functional annotation of the protein-coding genes in the O. fasciatus genome, we employed the local BLASTX and BLASTN programs and the Swiss-Prot database with an e-value ≤ 1e-5 to align the non-redundant nucleotides (NT) and the non-redundant proteins (NR), respectively".
Line 597 we revised "Kyoto Encyclopedia of Genes" as "and Kyoto Encyclopaedia of Genes".
Line 598 we revised "Finally" as "Ultimately".
Line 601 we added "the" in front of "tRNAscan-SE".
Line 609 we revised "gene family" as "gene families".
Line 610 we revised "of" as "the".
Line 611 we revised "in" as "using".
Line 774 we revised "relationship" as "relationships".
Line 775 we revised "single-copy gene" as "single-copy genes".
Line 776 we revised "length filter" as "a length filter". We also deleted ", respectively".
Line 778 we revised "sequence of each species" as "sequences for each species".
Line 782 we deleted "a" in front of "molecular clock".
Line 784-785 we revised "were clustered together with Larimichthys crocea belonged to" as "clustered with Larimichthys crocea in".
Line 787 we revised "about" as "approximately".
Line 788 we revised "Conclusion" as "Conclusions".
Line 791-793 we revised the sentence as "The final draft genome assembly is approximately 778.7 Mb, which was slightly higher than the estimated genome size (777.5 Mb) based on k-mer analysis".
Line 793-795 we revised the sentence as "Those contigs were scaffolded to chromosomes using Hi-C data, resulting a genome with a high level of continuity with a contig N50 of 2.1 Mb and a scaffold N50 of 33.5 Mb.".
Line 799-800 We revised the sentence "We found the divergence time between O. fasciatus and the common ancestor with Larimichthys crocea was at about 70.3-87.3 Ma" as "We found that the divergence time between O. fasciatus and its the common ancestor with Larimichthys crocea was approximately 70.3-87.3 Ma".

I wonder why the authors chose to sequence a female fish, while the male fish could have had provided the full sequence of the Y chromosome which could've brought insights into sex determination, the identification of sex specific regions, etc. I mention this because you stress that the genome assembly is useful for the understanding of these mechanisms this but then there's no mention of this important topic in the discussion.
Reply:
Thanks for the editor's concerns. We indeed have a plan for the genome assembly for a male one, after this female genome work. The reason we choose a female one because of the heterotropic chromosome in males. As far as we known, Y chromosomes exhibited lots of specific sequence characters, such as repeat content, comparing to X1 and X2, and those differences might increase the difficulty for the sequence assembly of chromosome X1 and X2. Based on this genome, the male genome assembly will be carried out in the following work, with the aim to get the accurate assembly of Y chromosome.
We have added the discussion in the conclusion in line 364-386 as follows: "As far as we

known, the Y chromosomes has always exhibited many specific sequence characteristics compared to X1 and X2, such as repeat content, and those differences might increase the difficulty of the sequence assembly of chromosomes X1 and X2. The chromosome-level genome assembly together with gene annotation data generated for the female fish in this work will provide a valuable resource for further research on sex-determining mechanisms, especially for obtaining an accurate assembly of the Y chromosome in male fish. These results will also accelerate genome-wide association studies in resistant breeding systems."

Reviewer3:
Further to my previous email, another referee noted that Oplegnathidae is no longer a part of the Perciformes, according to the Betancur-R et al. 2017 phylogenetic classification of fishes, who placed it in the Centrarchiformes. Please also include this detail in the introduction.
Reply:
Thanks for the editor's suggestions. We have carefully checked the two papers (Betancur-R. R, Broughton RE, Wiley EO, Carpenter K, López JA, Li C, Holcroft NI, Arcila D, Sanciangco M, Cureton II JC, Zhang F, Buser T, Campbell MA, Ballesteros JA, Roa-Varon A, Willis S, Borden WC, Rowley T, Reneau PC, Hough DJ, Lu G, Grande T, Arratia G, Ortí G. The Tree of Life and a New Classification of Bony Fishes. PLOS Currents Tree of Life. 2013 and Ricardo Betancur-R, Edward O. Wiley, Gloria Arratia, Arturo Acero, Nicolas Bailly, Masaki Miya,Guillaume Lecointre and Guillermo Ortí. Phylogenetic classification of bony fishes. 2017) and the book (Fishes of the World (Fifth Editon) (Joseph S. Nelson, Terry C. Grande and Mark V. Wilson)).
We agreed with the reviewer's suggestion and we also agreed with the molecular taxonomy results. We have revised the information in the abstract, introduction and Gene family identification and phylogenetic tree construction sections of the text. We have referenced the paper in the discussion section,

Close
