# Supplementary material for: Genome sequence of the barred knifejaw Oplegnathus fasciatus (Temminck & Schlegel, 1844): the first chromosome-level draft genome in the family Oplegnathidae
Source: Gigascience. 2019 Feb 1;8(3):giz013. doi: 10.1093/gigascience/giz013 (PMC6423371; doi:10.1093/gigascience/giz013)
Supplement: Response_to_Reviewer_Comments_Revision_1.pdf [file giz013_response_to_reviewer_comments_revision_1.pdf]

Author's Response To Reviewer Comments

Close

Editor reports:
Your manuscript "Genome sequence of the barred knifejaw Oplegnathus fasciatus (Temminck & Schlegel, 1844): the first chromosome-level draft genome in the family Oplegnathidae" (GIGA-D-18-00300R1) has been re-reviewed by our reviewers. Based on these reports, and my own assessment as Editor, I am pleased to inform you that it is potentially acceptable for publication in GigaScience, once you have carried out some final essential revisions suggested by our reviewers. Please also add the citation details for the GigaDB in the paper.
Reply:
Thanks a lot for the editor's suggestion. We have add the citation details for the GigaDB in the paper.
We also have revised the time of our subject as "Oplegnathus fasciatus (Temminck & Schlegel, 1844)".

Reviewer reports:
Reviewer #1: The authors have restructured and considerably improved the manuscript, accommodating most of my suggestions. I have some final comments, which are mostly cosmetic:

My previous comments 3/4, on the k-mer distribution - now at lines 112: this is still not very clear. I understand that the repeat content is based on fitting a model to the distribution. I do not fully agree that the peak labeled as repeated k-mers should be identified with generic repeat content, I think these are very clearly duplications (which are, of course, technically repeat content).
I would suggest to clarify the genome size calculation itself, which is now incorrect (line 112): 8.09 x10^10 / 100 = 777.5 Mb.
Reply:
We agreed with the reviewer's comment on that the peak labeled as repeated k-mers should be identified as generic repeat content. Strictly speaking, the majority of k-mers after the 1.8 times larger than the main depth (100 in our case) were most likely from the repeated regions, including the duplications that mentioned in the comment. That is also the way we estimated the repeat ratio of the genome.
We are sorry that the method for the genome size estimation was not clear enough. To clarify the method, the following formula were used : genome size = (Nk-mer – Nerror_k-mer) / D, where G is genome size, Nk-mer is the number of k-mers, Nerror_k-mer is the number of k-mers with the depth of 1, and D is the k-mer depth. The number of k-mers with depth of 1 were eliminated since k-mers with low depth were likely from the sequencing errors. As a result, the genome size was estimated as 777.5Mb. We have revised the description of genome size estimation method in the manuscript.

Line 132, 'complexity ... such as heterozygosity': This does not fit the very low heterozygosity levels just identified from the k-mer profile. Possibly structural variants instead of SNPs? I don't think the high duplication levels can explain this?

Reply:
We agreed with the reviewer’s comment on that genome complexity derived from the structural variants might also increase size of the genome assembly. So we revised the sentence as “The genome complexity, such as structural variants and heterozygosity might be possible reasons to explain the relative large genome size in the assembly.”
Line 162: 'filter all base sequences than 500 bp': more than 500 bp? Less than 500 bp?
Reply:
We would like to give sincere thanks to reviewer’s suggestions. We revised “filter all base sequences than 500 bp” as “filter all base sequences more than 500 bp”
There is a lot of redundancy between tables 1 & 3, I would suggest either merging these or moving the finer details of the assembly to table 3 (and keep table 1 as an overview of the final results, just N50/genome size/coverage).
Reply: Thanks a lot for the reviewer’s suggestion. We have merged the Table 3 to Table 1 to eliminate the information redundancy.
Table 2 would be more appropriate in the supplementary information.
Reply: Thanks a lot for the reviewer’s comment. The Table 2 was moved into the supplementary data according to the suggestion.

Close
