# Supplementary material for: Genome sequence of the barred knifejaw Oplegnathus fasciatus (Temminck & Schlegel, 1844): the first chromosome-level draft genome in the family Oplegnathidae
Source: Gigascience. 2019 Feb 1;8(3):giz013. doi: 10.1093/gigascience/giz013 (PMC6423371; doi:10.1093/gigascience/giz013)
Supplement: Reviewer_1_Report_Original_Submission -- Christiaan Henkel — 10/1/2018 Reviewed [file giz013_reviewer_1_report_original_submission.pdf]

Reviewer Report

Title: Genome sequence of the barred knifejaw Oplegnathus fasciatus (Temminck & Schlegel, 1844): the first chromosome-level draft genome in the family Oplegnathidae

Version: Original Submission **Date:** 10/1/2018

Reviewer name: Christiaan Henkel

Reviewer Comments to Author:

This manuscript describes the genome assembly and annotation of O. fasciatus, with little else by way of analysis. The methods used are mostly appropriate, and the assembly appears to be of high quality.
Some issues and suggestions:
1. The assembly contiguity is repeatedly referred to as 'remarkable', this is perhaps an exaggeration. These values are not extraordinary in the age of long-read sequencing. S Table 4 lists other fish assemblies, but includes almost no current-generation ones, flattering the assembly statistics obtained in this study.
2. I will admit I am not an expert on Oplegnathidae. However, according to Wikipedia, the genus Oplegnathus contains seven species, and the common name for O. fasciatus is 'striped beakfish' or 'barred knifejaw'. The manuscript claims two species (line 68), and the common name 'rock bream'.
3. Line 109: 'a repeat content of 38.46%', how was this calculated? It does not follow from figure 2.
4. Line 107/111: The k-mer estimate and the intial assembly yield exactly the same genome size (808.9 Mbp). This is highly unlikely, especially if the genome is highly repetitive, as claimed here.
5. Line 123: I assume the contigs and scaffold listed here, to which the HiC data map, are those of the final (PacBio-based) assembly. However, the only assembly that has been described at this point is the highly fragmented initial one. Perhaps you could restructure this so that the HiC sequencing is described after PacBio sequencing.
6. Figure 2 shows a clear bump corresponding to duplicated k-mers (at 200). Is this duplication level still relevant for the final assembly? For example, a lot of sequence is removed (line 136) based on redundancy, and a large fraction of PacBio reads do no map to the final assembly (line 158). Is there a relation with the sex chromosome configuration (X1X1X2X2, line 76)?
7. Line 140: That the polishing is performed using Pilon should be mentioned here (it is mentioned in S table 2). Also, 'using NGS data' is ambiguous, as PacBio also qualifies as NGS. This probably refers to Illumina only.
8. Figure 4 and S Figure 4 analyze O. fasciatus in the context of 'fish species'. While this is technically correct, it is biologically not always the most relevant comparison. Fish species such as ghost shark and lancelet are included, but for example tetrapods (which are more closely related to O. fasciatus than the aforementioned fish) are not. In figure 4, these make for less appropriate outgroups (because of their very distant relationship to the other, teleost, fish species). I would suggest including at least e.g. spotted gar to the analysis to fill this gap (and perhaps omit B. floridae).
8. Figure 4 needs more information in the legend. What do the numbers mean exactly, and how were they calculated? The conclusion drawn from this figure (line 266) is not appropriate, as the phylogenetic

position of Notothenioidei is not relevant to the narrative of this manuscript, and reclassification needs more evidence than this sparse phylogenetic tree.
9. One of the motivations for sequencing this genome is understanding the fish' sex determining system. This aim is not revisited in the results or Conclusion. How does the choice of a female individual for genome sequencing affect this goal?
Typos:
L 102 Hieq -> HiSeq
L 172 RepreatMasker -> RepeatMasker

Level of Interest

Please indicate how interesting you found the manuscript: Choose an item.

Quality of Written English

Please indicate the quality of language in the manuscript: Choose an item.

Declaration of Competing Interests

Please complete a declaration of competing interests, considering the following questions:

- Have you in the past five years received reimbursements, fees, funding, or salary from an organisation that may in any way gain or lose financially from the publication of this manuscript, either now or in the future?
- Do you hold any stocks or shares in an organisation that may in any way gain or lose financially from the publication of this manuscript, either now or in the future?
- Do you hold or are you currently applying for any patents relating to the content of the manuscript?
- Have you received reimbursements, fees, funding, or salary from an organization that holds or has applied for patents relating to the content of the manuscript?
- Do you have any other financial competing interests?
- Do you have any non-financial competing interests in relation to this paper?

If you can answer no to all of the above, write 'I declare that I have no competing interests' below. If your reply is yes to any, please give details below.

I declare I have no competing interests

I agree to the open peer review policy of the journal. I understand that my name will be included on my report to the authors and, if the manuscript is accepted for publication, my named report including any attachments I upload will be posted on the website along with the authors' responses. I agree for my report to be made available under an Open Access Creative Commons CC-BY license

(http://creativecommons.org/licenses/by/4.0/). I understand that any comments which I do not wish to be included in my named report can be included as confidential comments to the editors, which will not be published.

I agree to the open peer review policy of the journal

To further support our reviewers, we have joined with Publons, where you can gain additional credit to further highlight your hard work (see: https://publons.com/journal/530/gigascience). On publication of this paper, your review will be automatically added to Publons, you can then choose whether or not to claim your Publons credit. I understand this statement.

Yes Choose an item.
