# Supplementary material for: Genome sequence of the barred knifejaw Oplegnathus fasciatus (Temminck & Schlegel, 1844): the first chromosome-level draft genome in the family Oplegnathidae
Source: Gigascience. 2019 Feb 1;8(3):giz013. doi: 10.1093/gigascience/giz013 (PMC6423371; doi:10.1093/gigascience/giz013)
Supplement: Reviewer_1_Report_Revision_1 -- Christiaan Henkel — 1/7/2019 Reviewed [file giz013_reviewer_1_report_revision_1.pdf]

Reviewer Report

Title: Genome sequence of the barred knifejaw Oplegnathus fasciatus (Temminck & Schlegel, 1844): the first chromosome-level draft genome in the family Oplegnathidae

Version: Revision 1 **Date:** 1/7/2019

Reviewer name: Christiaan Henkel

Reviewer Comments to Author:

The authors have restructured and considerably improved the manuscript, accommodating most of my suggestions. I have some final comments, which are mostly cosmetic:
My previous comments 3/4, on the k-mer distribution - now at lines 112: this is still not very clear. I understand that the repeat content is based on fitting a model to the distribution. I do not fully agree that the peak labeled as repeated k-mers should be identified with generic repeat content, I think these are very clearly duplications (which are, of course, technically repeat content).
I would suggest to clarify the genome size calculation itself, which is now incorrect (line 112): 8.09 x10^10 / 100 = 777.5 Mb.
Line 132, 'complexity ... such as heterozygosity': This does not fit the very low heterozygosity levels just identified from the k-mer profile. Possibly structural variants instead of SNPs? I don't think the high duplication levels can explain this?
Line 162: 'filter all base sequences than 500 bp': more than 500 bp? Less than 500 bp?
There is a lot of redundancy between tables 1 & 3, I would suggest either merging these or moving the finer details of the assembly to table 3 (and keep table 1 as an overview of the final results, just N50/genome size/coverage).
Table 2 would be more appropriate in the supplementary information.

Level of Interest

Please indicate how interesting you found the manuscript: Choose an item.

Quality of Written English

Please indicate the quality of language in the manuscript: Choose an item.

Declaration of Competing Interests

Please complete a declaration of competing interests, considering the following questions:

- Have you in the past five years received reimbursements, fees, funding, or salary from an organisation that may in any way gain or lose financially from the publication of this manuscript, either now or in the future?

- Do you hold any stocks or shares in an organisation that may in any way gain or lose financially from the publication of this manuscript, either now or in the future?
- Do you hold or are you currently applying for any patents relating to the content of the manuscript?
- Have you received reimbursements, fees, funding, or salary from an organization that holds or has applied for patents relating to the content of the manuscript?
- Do you have any other financial competing interests?
- Do you have any non-financial competing interests in relation to this paper?

If you can answer no to all of the above, write 'I declare that I have no competing interests' below. If your reply is yes to any, please give details below.

I declare that I have no competing interests

I agree to the open peer review policy of the journal. I understand that my name will be included on my report to the authors and, if the manuscript is accepted for publication, my named report including any attachments I upload will be posted on the website along with the authors' responses. I agree for my report to be made available under an Open Access Creative Commons CC-BY license (http://creativecommons.org/licenses/by/4.0/). I understand that any comments which I do not wish to be included in my named report can be included as confidential comments to the editors, which will not be published.

I agree to the open peer review policy of the journal

To further support our reviewers, we have joined with Publons, where you can gain additional credit to further highlight your hard work (see: https://publons.com/journal/530/gigascience). On publication of this paper, your review will be automatically added to Publons, you can then choose whether or not to claim your Publons credit. I understand this statement.

Yes Choose an item.
