# Supplementary material for: Genome sequence of the barred knifejaw Oplegnathus fasciatus (Temminck & Schlegel, 1844): the first chromosome-level draft genome in the family Oplegnathidae
Source: Gigascience. 2019 Feb 1;8(3):giz013. doi: 10.1093/gigascience/giz013 (PMC6423371; doi:10.1093/gigascience/giz013)
Supplement: Reviewer_2_Report_Original_Submission -- Alejandro Gutierrez — 10/3/2018 Reviewed [file giz013_reviewer_2_report_original_submission.pdf]

Reviewer Report

Title: Genome sequence of the barred knifejaw Oplegnathus fasciatus (Temminck & Schlegel, 1844): the first chromosome-level draft genome in the family Oplegnathidae

Version: Original Submission **Date:** 10/3/2018

Reviewer name: Alejandro Gutierrez

Reviewer Comments to Author:

In this manuscript Xiao et al. reports the genome assembly of the rock bream (O. fasciatus) a species of increasing economic importance in Asia. This species exhibits sex dimorphism in growth and also a sex determination system based on multiple sex chromosomes X1X1X2X2/X1X2Y, which makes it interesting species to study. The draft genome of the rock bream will be a valuable resource to facilitate future research aimed at improving relevant traits and understanding of determination systems.
The authors used an adequate amount of sequence data from three different sources (Illumina short reads, PacBio and Hi-C), which allowed them to generate a robust genome assembly. Furthermore, the authors annotated the genome using multiple strategies. Finally, they carried out some phylogenetic analyses including other fish species. The methods followed to obtain the assembly are good in general, and well described.
L33-L35 Please re-phrase, maybe say "sexual dimorphism in growth"
L37 ...basing -> based
L43 "…We assembled the O.fasciatus" <genome?>
L77 Again please re-phrase "the growth sexual dimorphism"
L99-L100 "A whole genome using Illumina DNA seq…" re-phrase
L115 Was the blood extracted from the same fish used for pacBio and Illumina?
L162 "the results showed <that> 99.8%.."
L172 Typo Repreatmasker
L252 Is not clear how you came up to those 812 orhtogroups, and the same for L256
L266 I don't think the authors should claim that the Notothenioidei should be elevated to the order level, but I would accept that their results suggest or show evidence of this.
General Comments:
There are many issues with the English throughout the manuscript and these must be addressed before considering for publication. I strongly encourage the authors to proof-read the manuscript before re-submitting.
I wonder why the authors chose to sequence a female fish, while the male fish could have had provided the full sequence of the Y chromosome which could've brought insights into sex determination, the identification of sex specific regions, etc. I mention this because you stress that the genome assembly is useful for the understanding of these mechanisms this but then there's no mention of this important topic in the discussion.

Level of Interest

Please indicate how interesting you found the manuscript: Choose an item.

Quality of Written English

Please indicate the quality of language in the manuscript: Choose an item.

Declaration of Competing Interests

Please complete a declaration of competing interests, considering the following questions:

- Have you in the past five years received reimbursements, fees, funding, or salary from an organisation that may in any way gain or lose financially from the publication of this manuscript, either now or in the future?
- Do you hold any stocks or shares in an organisation that may in any way gain or lose financially from the publication of this manuscript, either now or in the future?
- Do you hold or are you currently applying for any patents relating to the content of the manuscript?
- Have you received reimbursements, fees, funding, or salary from an organization that holds or has applied for patents relating to the content of the manuscript?
- Do you have any other financial competing interests?
- Do you have any non-financial competing interests in relation to this paper?

If you can answer no to all of the above, write 'I declare that I have no competing interests' below. If your reply is yes to any, please give details below.

I declare that I have no competing interests

I agree to the open peer review policy of the journal. I understand that my name will be included on my report to the authors and, if the manuscript is accepted for publication, my named report including any attachments I upload will be posted on the website along with the authors' responses. I agree for my report to be made available under an Open Access Creative Commons CC-BY license (http://creativecommons.org/licenses/by/4.0/). I understand that any comments which I do not wish to be included in my named report can be included as confidential comments to the editors, which will not be published.

I agree to the open peer review policy of the journal

To further support our reviewers, we have joined with Publons, where you can gain additional credit to further highlight your hard work (see: https://publons.com/journal/530/gigascience). On publication of this paper, your review will be automatically added to Publons, you can then choose whether or not to claim your Publons credit. I understand this statement.

Yes Choose an item.
